# Synthesis and Transformation of (-)-Isopulegol-Based Chiral *β*-Aminolactones and *β*-Aminoamides

**DOI:** 10.3390/ijms19113522

**Published:** 2018-11-08

**Authors:** Tam Minh Le, Péter Bérdi, István Zupkó, Ferenc Fülöp, Zsolt Szakonyi

**Affiliations:** 1 Institute of Pharmaceutical Chemistry, University of Szeged, H-6720 Szeged, Eötvös utca 6, Hungary; leminhtam@pharm.u-szeged.hu (T.M.L.); fulop@pharm.u-szeged.hu (F.F.); 2 Stereochemistry Research Group of the Hungarian Academy of Sciences, H-6720 Szeged, Eötvös utca 6, Hungary; 3 Department of Pharmacodynamics and Biopharmacy, University of Szeged, H-6720 Szeged, Eötvös utca 6, Hungary; berdi.peter@pharm.u-szeged.hu (P.B.); zupko@pharm.u-szeged.hu (I.Z.); 4 Interdisciplinary Centre of Natural Products, University of Szeged, H-6720 Szeged, Eötvös utca 6, Hungary

**Keywords:** terpenoid, *β*-aminolactones, *β*-aminoamides, dipeptide, antiproliferative activity

## Abstract

A library of isopulegol-based *β*-amino acid derivatives has been developed from commercially-available (-)-isopulegol. Michael addition of primary and secondary amines towards *α*,*β*-unsaturated *γ*-lactones was accomplished resulting in *β*-aminolactones in highly-stereoselective reactions. Ring-opening of *β*-aminolactones with different amines furnished excellent yields of *β*-aminoamides. Moreover, the applicability of aminolactones in peptide synthesis was examined by opening the lactone ring with *α*- and *β*-aminoesters, providing dipeptides as promising chiral substrates for the synthesis of foldamers. The antiproliferative activities of *β*-aminolactones and *β*-aminoamides were explored, and the structure-activity relationships were studied from the aspects of the stereochemistry of the monoterpene ring and the substituent effects on the *β*-aminoamide ring system. The *N*-unsubstituted (-)-isopulegol-based *β*-aminoamides exhibited considerable antiproliferative activity against a panel of human adherent cancer cell lines (HeLa, MCF7 and MDA-MB-231).

## 1. Introduction

Sesquiterpene lactones containing the *α*-methylene-*γ*-lactone moiety are natural products occurring in many plant families. These compounds are known for their various biological activities, including cytotoxicity to tumor cells, anti-bacterial, antifungal, and anti-protozoan activities, as well as activity against human and animal parasites or inhibition of plant growth [1,2,3]. 

Conjugate addition of nucleophiles to *α*-methylene-*γ*-lactones provides *β*-aminolactones, which increase the proportion of cells in the G2/M and S phase [3] and serve as water-soluble derivatives that might retain cytotoxicity through a prodrug mechanism [4]. Additionally, the transformation of *β*-aminolactones, formally *β*-amino esters, to their derivatives such as 1,3-aminalcohols, proved to use those chiral auxiliaries in the enantioselective synthesis of secondary alcohols or other pharmacons, e.g., esomeprazole [5,6,7,8,9]. Besides their value in enantioselective catalysis, 1,3-aminoalcohols are also excellent building blocks for the synthesis of various heterocyclic ring systems, such as 1,3-oxazines, 1,3-thiazines or 1,4-oxazepams [10,11]. 2-Imino-1,3-thiazines and 2-iminothiazolidines can be found as structural units in biologically-relevant compounds, including antifungal and antimicrobial agents [12], BACE1 inhibitors [13], or cannabinoid receptor agonists [14,15,16].

In addition, ring-opening of *β*-aminolactones with different amines may provide *β*-aminoamides, which are well-known subunits of biologically-important compounds such as bestatin, a potent aminopeptidase B. Its usefulness in the treatment of cancer through its ability to enhance the cytotoxic activity of known antitumor agents is well-known [17,18]. *β*-Aminoamides exhibit other biological activities as well, such as antidiabetic [19], HIV-protease, or renin inhibitor effects [20]. Besides interest in the synthesis of *β*-aminoamides, the opening of *β*-aminolactones with *β*-aminoesters is a useful method for the synthesis of dipeptides containing *β*-alanine moiety. *β*-Alanine is a precursor of the antioxidant dipeptide carnosine (*β*-alanine-l-histidine), which is thought to increase cell viability via an anti-senescence mechanism [21]. *β*-Ala-Gln has been applied in medical fields as a component of patient infusions [22]. Furthermore, *β*-alanine transporters were found to be highly upregulated in antibody-producing cell lines, indicating the cell’s requirement for this amino acid [21].

Herein, our aim was to develop a library of monoterpene-based *β*-aminolactones and *β*-aminoamides by applying commercially-available natural (-)-isopulegol as an inexpensive chiral source, and to study their antiproliferative activity on multiple cancer cell lines. Moreover, we also report the synthesis of (-)-isopulegol-based dipeptides, which might serve as promising chiral substrates for the synthesis of chiral foldamers.

## 2. Results

### 2.1. Synthesis of α-Methylene-γ-Butyrolactones

**Figure 1:**
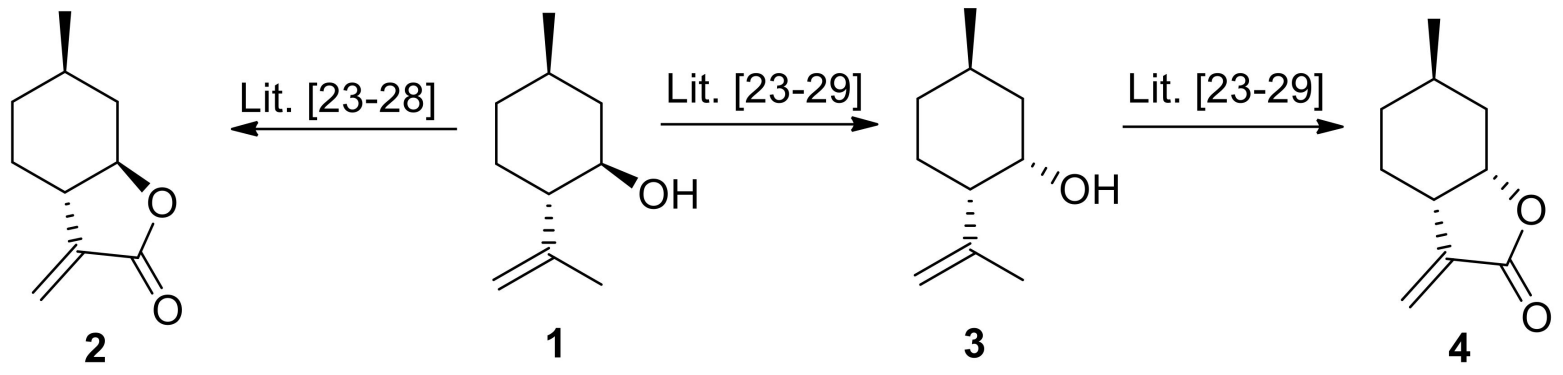
Synthesis of (-)-isopulegol-based *α*-methylene-*γ*-butyrolactones **2** and **4**.

The key intermediate (+)-*α*-methylene-*γ*-butyrolactone **2** was prepared from commercially-available (-)-isopulegol **1** with regioselective hydroxylation, followed by two-step oxidation and ring closure of the obtained *γ*-hydroxy-substituted *α*,*β*-unsaturated carboxylic acid by applying literature methods [23,24,25,26,27,28] (Figure 1). The diastereoisomeric (-)-*α*-methylene-*γ*-butyrolactone **4** was prepared by starting similarly from (-)-isopulegol **1**. In the first step, the hydroxy group of **1** was oxidized, followed by stereoselective reduction of the resulting carbonyl group providing (+)-neoisopulegol **3** [23,24,25,26,27,28,29] (Figure 1). 

### 2.2. Synthesis of β-Aminolactones

**Scheme 1:**
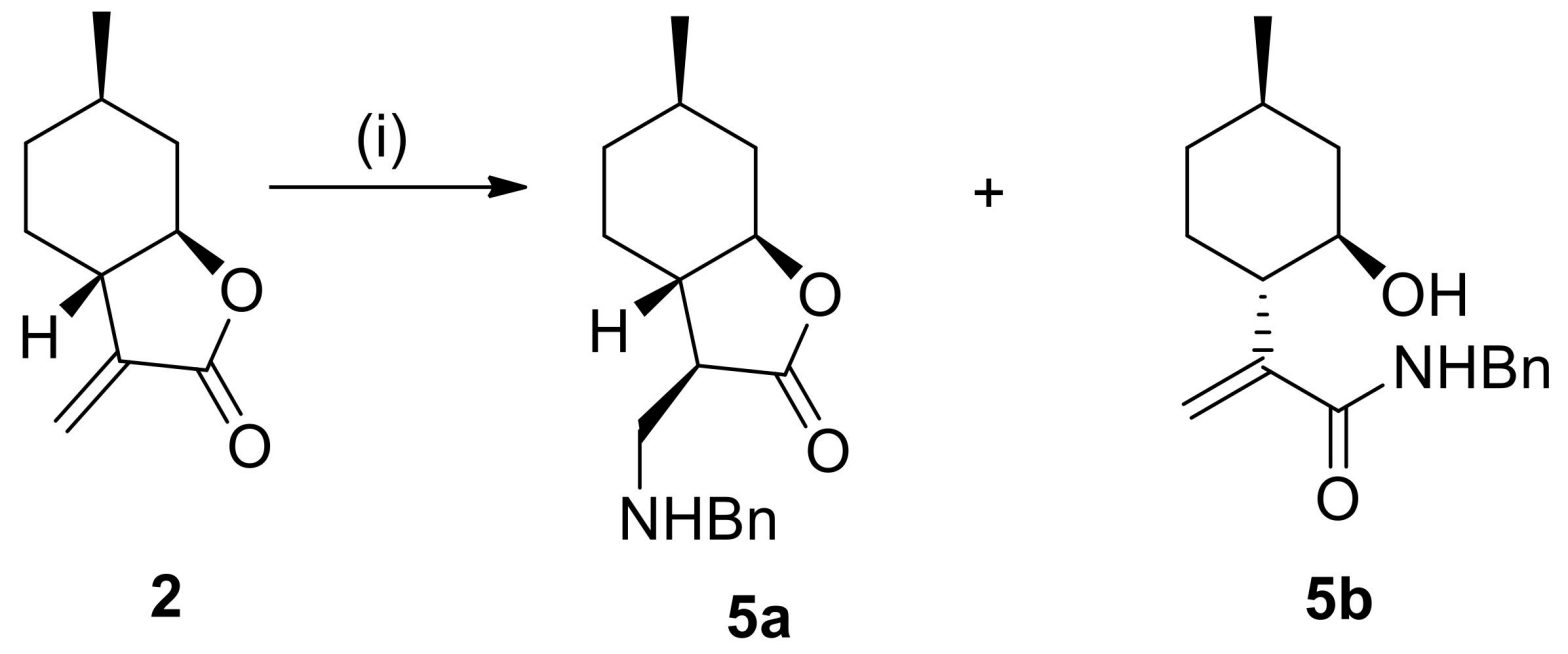
(**i**) Benzylamine (1 equiv.), dry EtOH, 25 °C, 20 h.

Nucleophilic addition of primary and secondary amines to *α*-methylene-*γ*-butyrolactones **2** and **4** has proven to be an efficient method for the preparation of a highly-diversified library of *β*-aminolactones [3,30]. When the addition of one equivalent of benzylamine to **2** was performed as a model reaction, the formation of *N*-benzyl aminolactone **5a** and *N*-benzyl methylene amide **5b** (the latter could not be isolated in pure form) was observed. The effect of the solvent was also studied, and it was found that the applied solvent strongly affected the yield of **5a** and the ratio of the two products (Scheme 1, Table 1).

When alcohols as protic solvents were used, formation of **5a** was observed as the main product. Among of three protic solvents applied, EtOH gave target **5a** with the best chemoselectivity (entry 7). The ratio of **5a** and **5b** also depended on temperature. In alcohols, in turn, product ratios were similar at low (0 °C) and high (25 °C) temperature (compare entries 6 and 7). Furthermore, the yield of **5a** increased with temperature. At higher temperatures, however, the yield of **5a** dropped, and the products were formed in a ratio of 4:1, even with decreasing reaction time (Table 1).

**Scheme 2:**
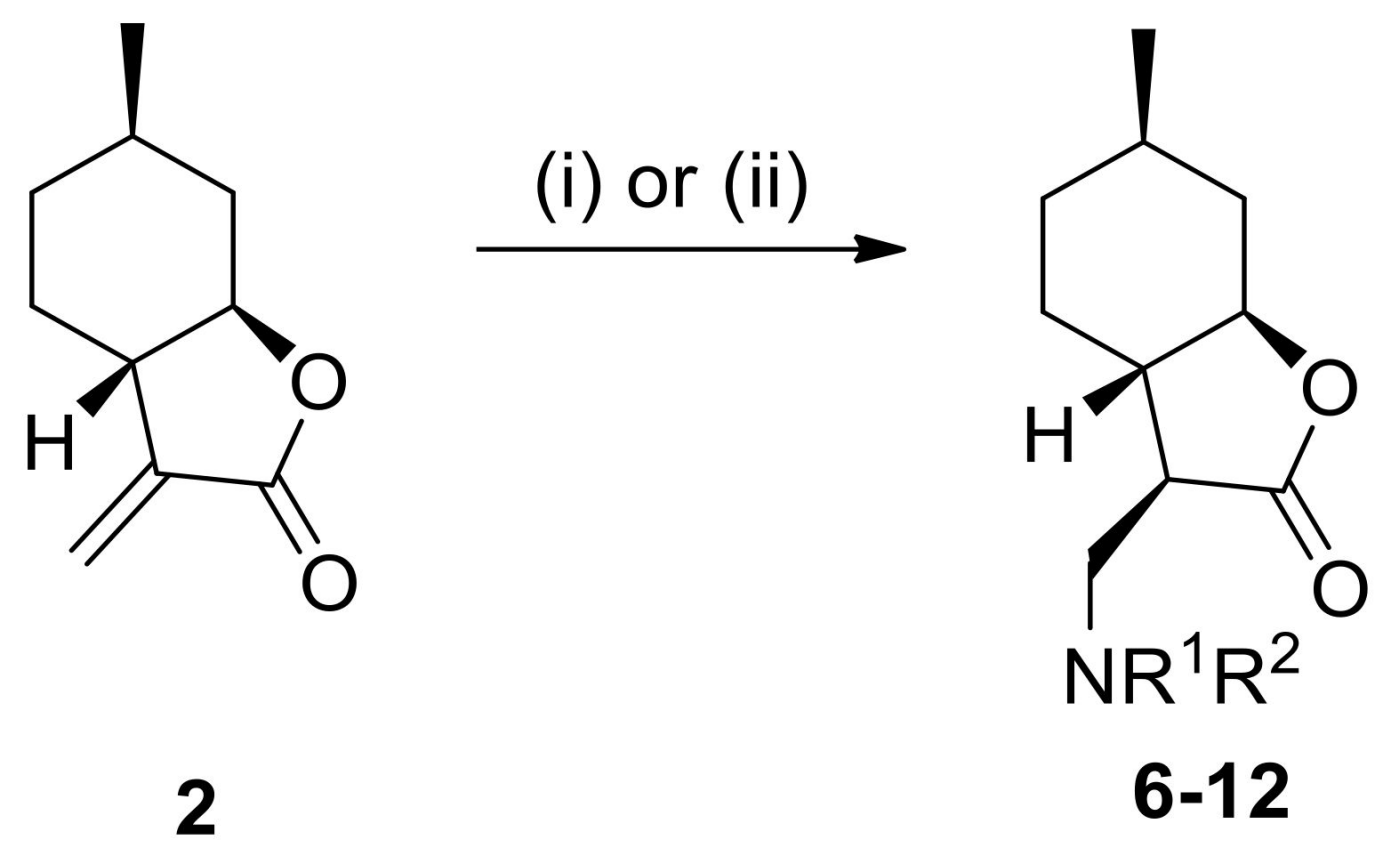
(**i**) R^1^R^2^NH (1 equiv.), dry EtOH, 25 °C, 20 h, for **6**–**10**; (**ii**) aminoester. HCl (2 equiv.), Et_3_N (2 equiv.), dry EtOH, 25 °C, 20 h, for **11** and **12**.

After optimizing the condition for nucleophilic addition with benzylamine, amine adducts **6**–**10** were synthesized from **2** under these conditions (one equivalent of appropriate amine, EtOH, 25 °C) (Scheme 2). Surprisingly, when (*R*)-and (*S*)-*α*-methylbenzylamine and secondary amines were applied, only the formation of aminolactones was observed (Table 2). This is probably due to the steric hindrance of these amines. Besides amines, the best conditions were also successful for the addition of L- or *β*-aminoesters as amine sources to prepare some *β*-aminolactones containing aminoester moiety **11**–**12** (Table 2).

**Scheme 3:**
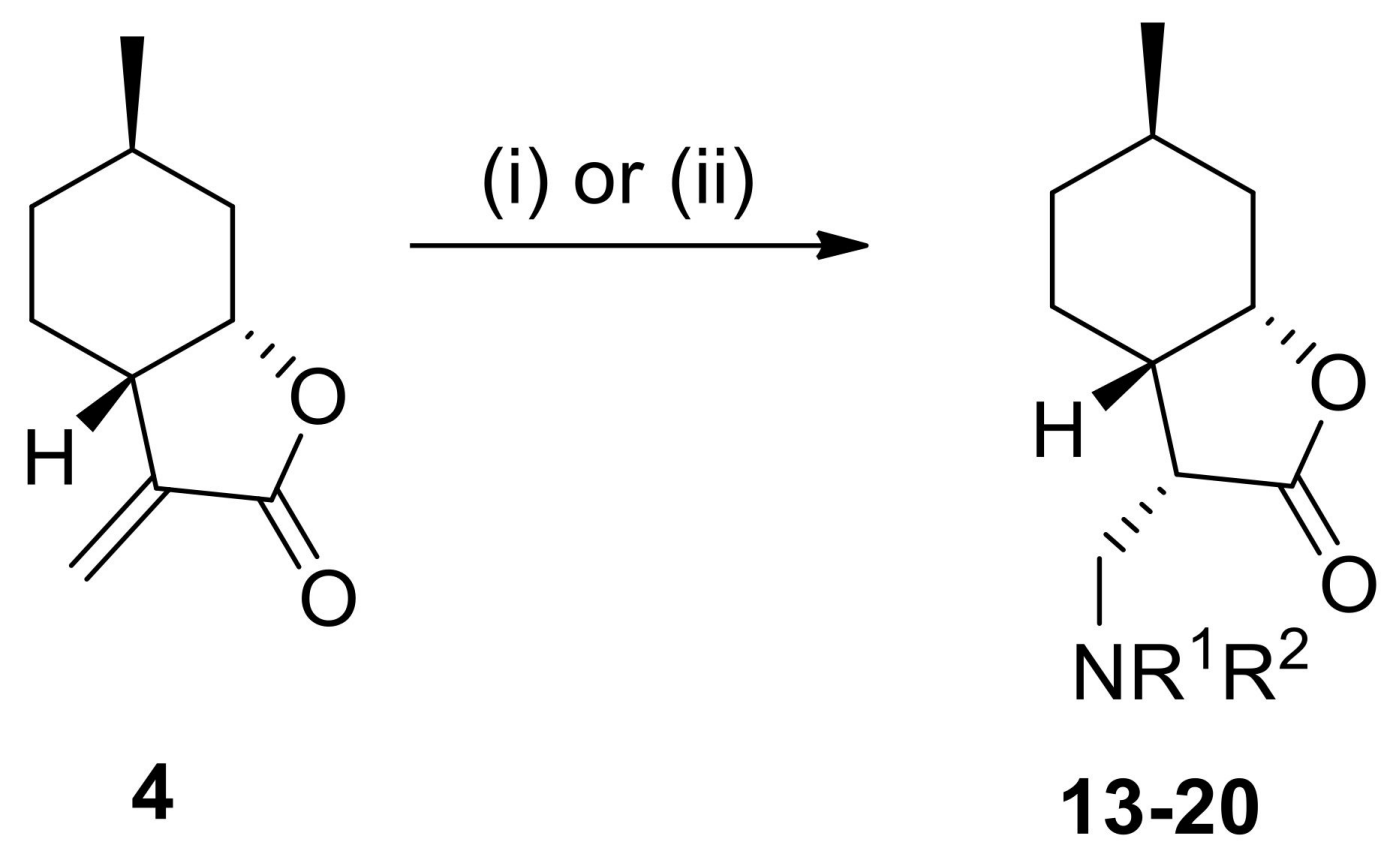
(**i**) R^1^R^2^NH (1 equiv.), dry EtOH, 25 °C, 20 h for **13**–**18**; (**ii**) aminoester. HCl (2 equiv.), Et_3_N (2 equiv.), dry EtOH, 70 °C, 20 h, for **19** and **20.**

The optimized conditions were also applied for the preparation of (+)-neoisopulegol-based *β*-aminolactones **13**–**18** starting from **4** (Scheme 3). Interestingly, under the applied conditions, exclusive formation of the amine adducts was observed. This may be due to the *cis* configuration of **4**, which makes the lactone more hindered for nucleophilc attack (Table 3). The reaction of **4** with some aminoesters was effective at an elevated temperature to achieve aminoester-based *β*-aminolactone derivatives **19**–**20** (Table 3).

**Figure 2:**
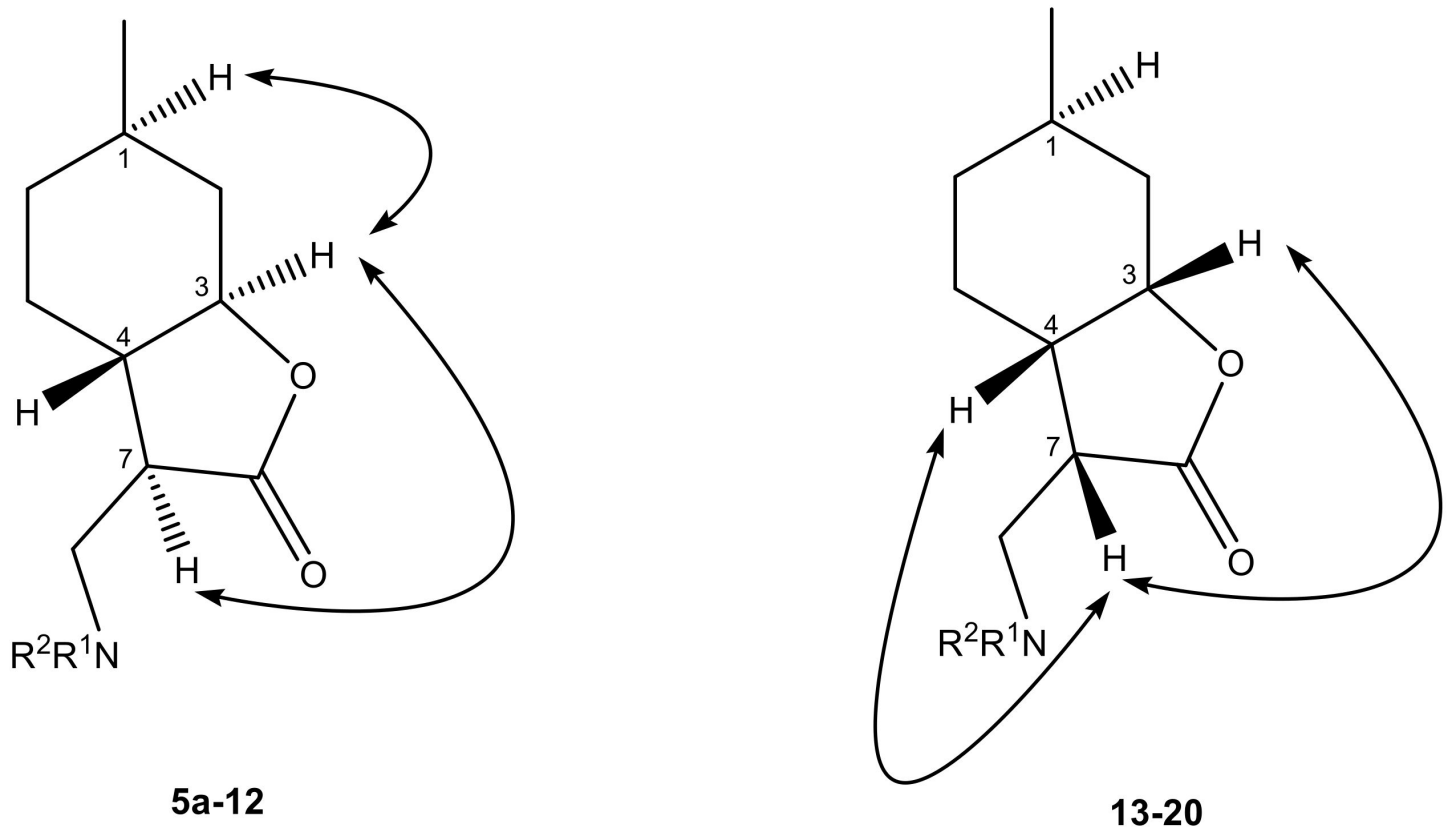
Determination of the relative configuration of *β*-aminolactones by NOESY.

The relative configuration of compounds **5a**–**12** and **13**–**20** was determined by means of NOESY experiments. Clear NOE signals were observed between the H-1 and H-3, as well as the H-3 and H-7 protons in the case of **5a**–**12**, while significant NOE signals were shown between the H-3 and H-7, as well as the H-4 and H-7 protons in the case of **13**–**20** (Figure 2).

### 2.3. Synthesis of β-Aminoamides and Dipeptides

**Scheme 4:**
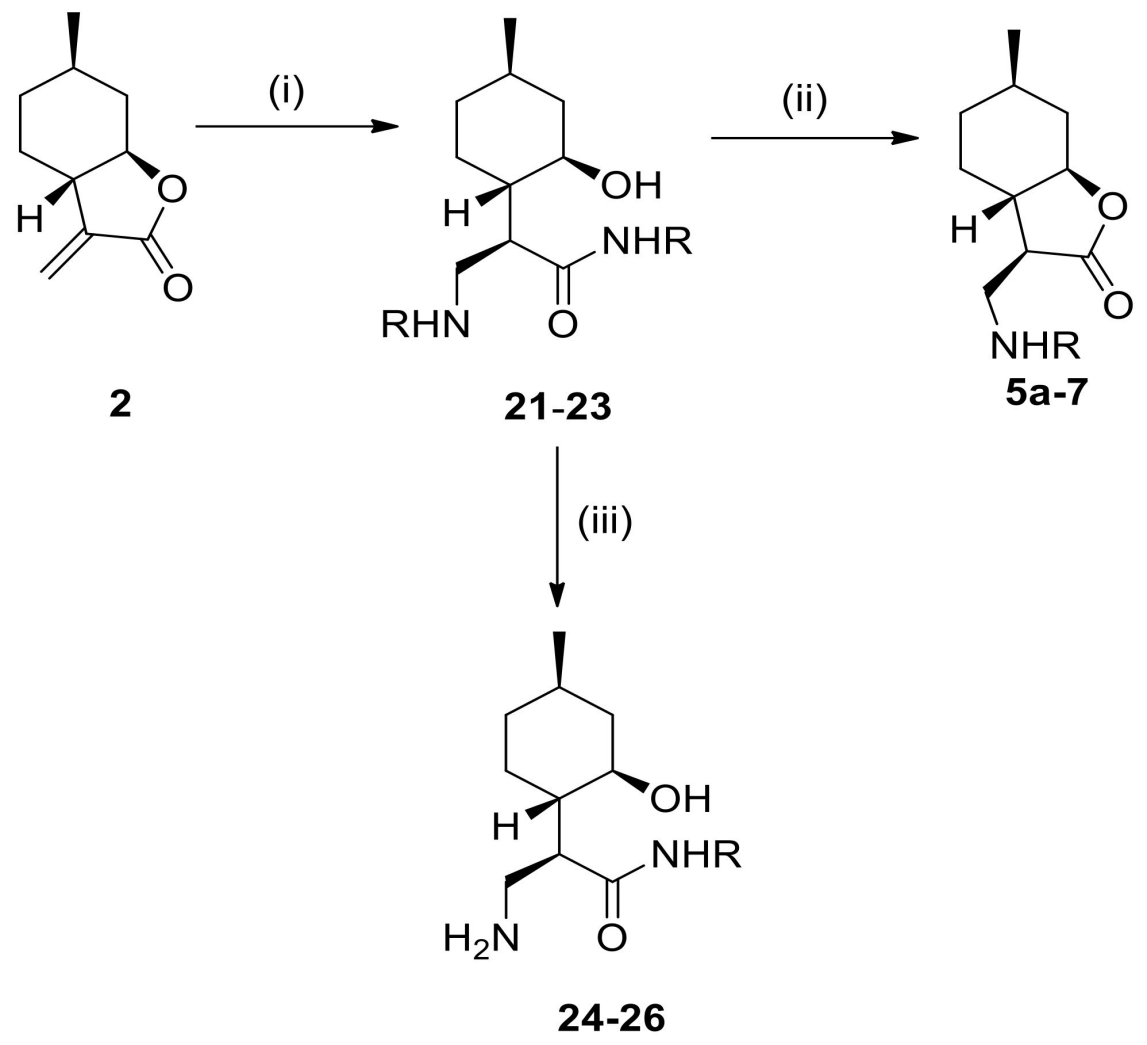
(**i**) RNH_2_ (4 equiv.), dry EtOH; (**ii**) 10% aqueous HCl solution, 25 °C, 24 h, **5a**: 70%, **6**, **7**: 65%; (**iii**) catalyst, H_2_ (1 atm.), MeOH, 25 °C, 62–80%.

Nucleophilic addition and ring-opening of lactones were simultaneously performed from **2** using excess amines to form *β*-aminoamides **21**–**23** in one step (Scheme 4). It is interesting that benzylamine reacted at room temperature, while (*R*)- and (*S*)-*α*-methylbenzylamine required a higher temperature and longer reactions (Table 4). This is probably due to steric hindrance exerted by the *α*-methyl group. Our efforts in the opening of lactones with secondary amines failed. Hydrolysis of *β*-aminoamides under acidic conditions resulted in the original starting material *β*-aminolactones **5a**–**7** (Scheme 4).

Debenzylation via hydrogenolysis of compounds **21**–**23** over appropriate catalysts in MeOH gave primary aminoamides **24**–**26** in moderate yields (Table 5).

**Scheme 5:**
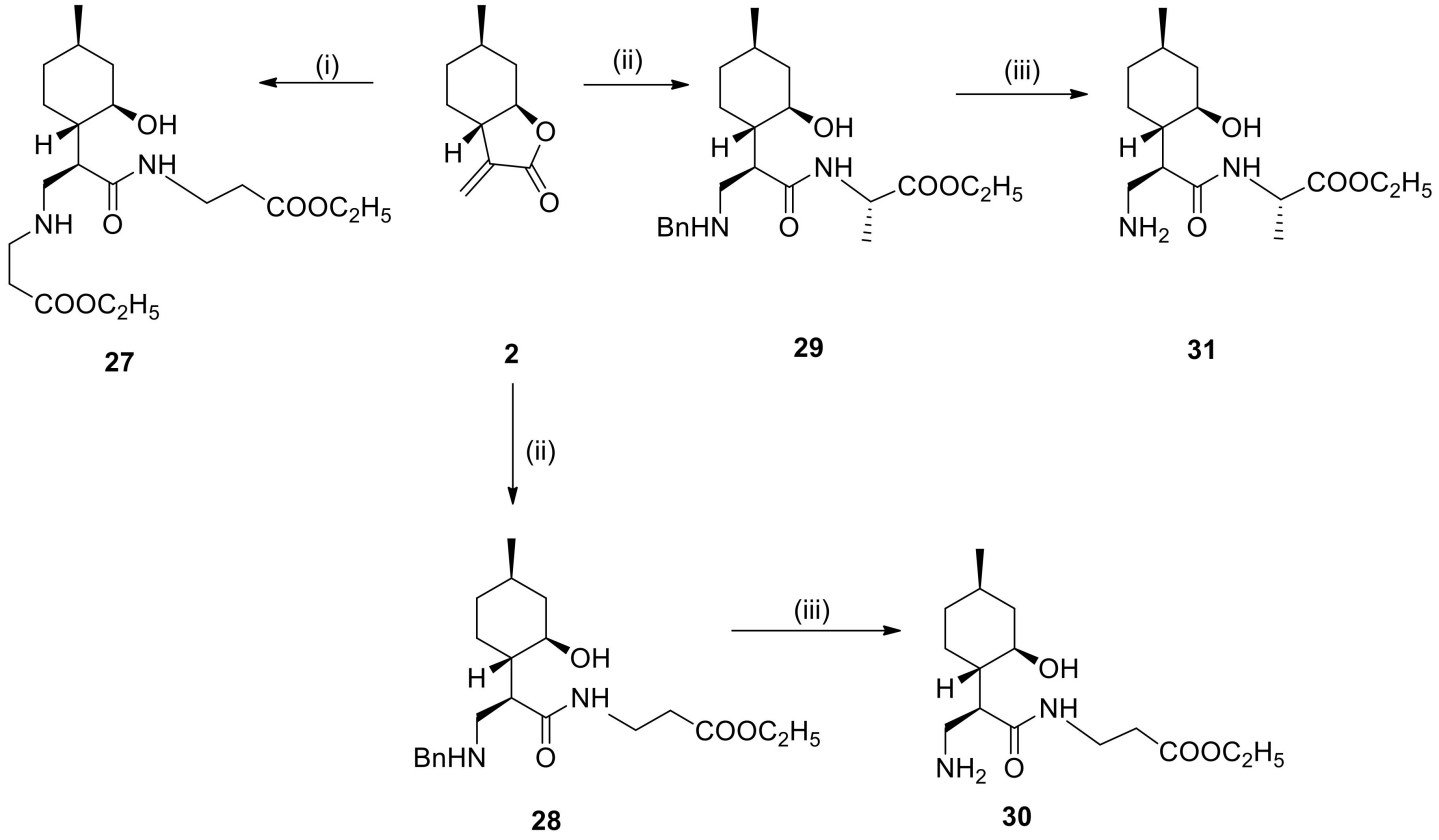
(**i**) NH_2_C_2_H_5_COOC_2_H_5_ (3 equiv.), dry EtOH, 25 °C, 48 h, 63%; (**ii**) Benzylamine (1 equiv.), dry EtOH, 25 °C, 20 h, 65%; then amino esters (3 equiv.), dry EtOH, 70 °C, 48 h, 40–45%; (**iii**) 5% Pd/C, dry EtOH, 25 °C, 24 h, 50–55%.

In further studies starting from **2**, the addition and ring-opening reaction with *β*-aminoester successfully gave dipeptide **27**. The application of *α*-aminoesters failed despite using long reaction times and elevated temperatures. The probable reason is steric hindrance exerted by the *α*-methyl group of the aminoesters. In addition, the opening of *N*-benzyl aminolactone **5a** with both the *α*- and *β*-aminoester proceeded smoothly to give *N*-benzyl dipeptides **28**–**29**. Debenzylation through hydrogenolysis over Pd/C and purification of the crude products gave dipeptides **30**–**31**, i.e., suitable starting compounds in peptide synthesis (Scheme 5).

**Scheme 6:**
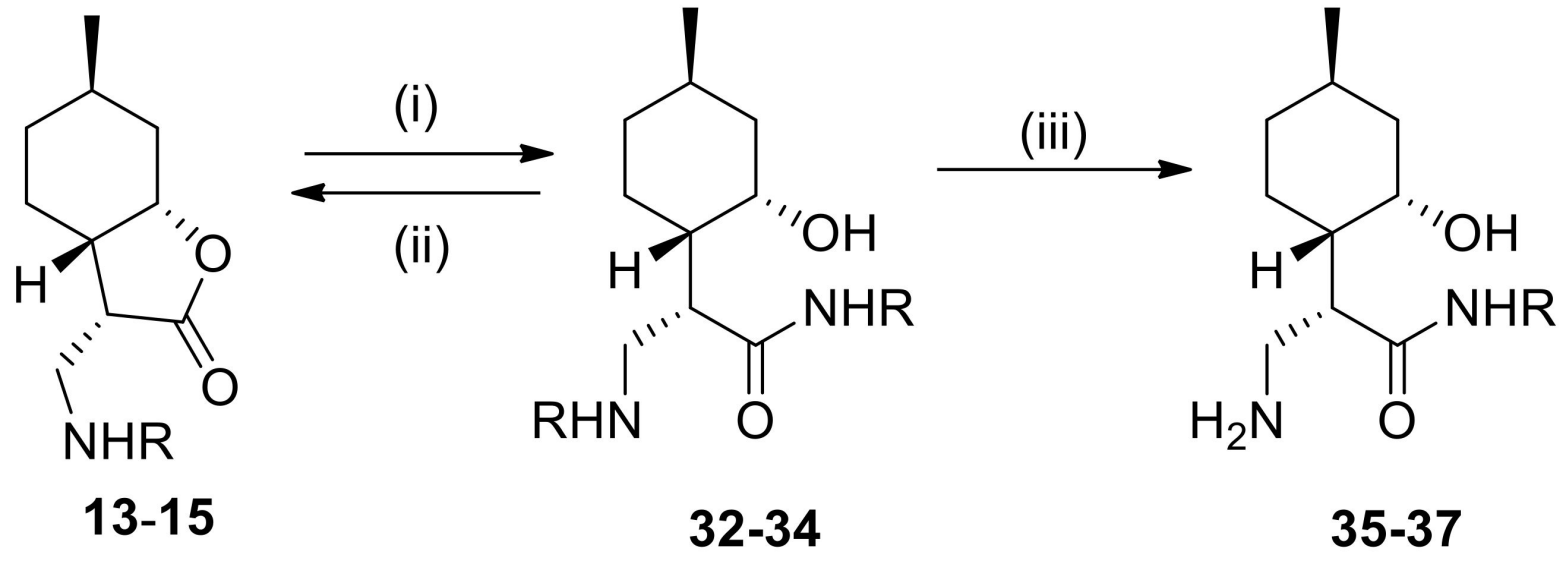
(**i**) RNH_2_ (4 equiv.), dry THF; 70 °C; (**ii**) 10% aqueous HCl solution, 25 °C, 24 h, **13**: 60%, **14**, **15**: 70%; (**iii**) catalyst, H_2_ (1 atm), MeOH, 25 °C, 52–70%.

Our effort to prepare *β*-aminoamides **32**–**34** starting from **4** failed. Fortunately, the synthesis was achieved by reacting *β*-aminolactones **13**–**15** with primary amines under reflux conditions in anhydrous THF [31] (Table 4). Again, opening the lactone ring with secondary amines was unsuccessful. Acidic hydrolysis of *β*-aminoamides **26**–**28** led to the original starting material *β*-aminolactones **13**–**15** instead of the expected *β*-aminoacids (Scheme 6). Debenzylation with appropriate catalysts gave primary *β*-aminoamides **35**–**37** in moderate yields (Table 5). The attempted nucleophilic addition and ring-opening of **6** with *α*- or *β*-aminoesters failed.

### 2.4. Antiproliferative Activities

Since several sesquiterpene-based *α*-methylene-*γ*-lactones, as well as their derivatives containing *β*-aminolactone moiety, exerted an antiproliferative action on adherent human cancer cell lines [3,30], antiproliferative activities of the prepared *β*-aminolactone and *β*-aminoamide analogues were also tested against a panel of human malignant cell lines isolated from cervical (HeLa) and breast (MCF7 and MDA-MB-231) cancers (Table 6). While the *β*-aminolactone-typed monoterpene derivatives proved to be ineffective against the utilized cell lines, the *N*-(*S*)-*α*-methylbenzyl-substituted *β*-aminoamide analogues (**23**, **34**) exhibited modest growth inhibitory activities. The most potent newly-prepared monoterpene analogue was compound **23**, exerting antiproliferative activity comparable to those of reference agent cisplatin.

*α*-Methylene-*γ*-lactone is generally believed to be a pharmacophore acting as an alkylating agent on DNA and proteins [32]. In the present set of (-)-isopulegol analogs, the *γ*-lactone-type derivatives (**2**, **4**, **5** and **7**) exerted weak antiproliferative activities, while the most active member of the presented library (**23**) is not a typical sesquiterpene lactone, but a *β*-aminoamide. Based on our results, the stereochemistry of the *N*-substituent on the amide function ((*S*)-*α*-methylbenzyl substituent), as well as the *trans* position of the bulky *β*-aminoamide substituent and the hydroxy group on the cyclohexane ring, are proposed as crucial conditions accounting for the activity. The antiproliferative activity of dipeptides **27**, **28,** and **29** was also tested on adherent human cancer cell lines. While in case of **29** a week antiproliferative activity was observed, on MCF7 and MDA-MB-231, **27** and **28** were uneffective. 

## 3. Discussion

Starting from commercially-available (-)-isopugeol, a new family of isopulegol- and neoisopulegol-based chiral *β*-aminolactone and *β*-aminoamide libraries has been prepared through chiral *α*-methylene-*γ*-lactones as key intermediates. Moreover, isopulegol-based chiral dipeptides, promising chiral substrates for the synthesis of chiral foldamers, were synthesized. The resulting *β*-aminoamides exert marked antiproliferative action on a panel of human cancer cell lines. In vitro pharmacological studies have clearly shown that the *N*-(*S*)-*α*-methylbenzyl substituent on the *β*-aminoamide function is essential. The stereochemistry of the *β*-aminoamides has no influence on the antiproliferative effect.

## 4. Materials and Methods

### 4.1. General Methods

Commercially-available compounds were used as obtained from suppliers (Molar Chemicals Ltd., Halásztelek, Hungary; Merck Ltd., Budapest, Hungary and VWR International Ltd., Debrecen, Hungary), while applied solvents were dried according to standard procedures. Optical rotations were measured in MeOH at 20 °C, with a Perkin-Elmer 341 polarimeter (PerkinElmer Inc., Shelton, CT, USA). Chromatographic separations and monitoring of reactions were carried out on Merck Kieselgel 60 (Merck Ltd., Budapest, Hungary). Elemental analyses for all prepared compounds were performed on a Perkin-Elmer 2400 Elemental Analyzer (PerkinElmer Inc., Waltham, MA, USA). GC measurements for direct separation of commercially-available enantiomers of isopulegol to determine the enantiomeric purity of starting material **1** were performed on a Chirasil-DEX CB column (2500 × 0.25 mm I.D.) on a Perkin-Elmer Autosystem XL GC equipped with a Flame Ionization Detector (Perkin-Elmer Corporation, Norwalk, CT, USA) and a Turbochrom Workstation data system (Perkin-Elmer Corp., Norwalk, CT, USA). Melting points were determined on a Kofler apparatus (Nagema, Dresden, Germany) and are uncorrected. ^1^H- and ^13^C-NMR spectra were recorded on BruckerAvance DRX 500 spectrometer [500 MHz (^1^H) and 125 MHz (^13^C), δ = 0 (TMS)]. Chemical shifts are expressed in ppm (δ) relative to TMS as the internal reference. *J* values are given by Hz. The structures were confirmed by 1H-NMR, 13C-NMR and 2D-NMR technics (see Supplementary Materials).

### 4.2. Starting Materials

(*-*)-Isopulegol (**1**) is available commercially from Merck Co (Darmstadt, Germany) with *ee* = 95%. (+)-*α*-Methylene-*γ*-butyrolactone (**2**), (+)-neoisopulegol (**3**) and (-)-*α*-methylene-*γ*-butyrolactone (**4**) were prepared according to literature procedures. All spectroscopic data were similar to those described therein [25,27].

### 4.3. General Procedure for Nucleophilic Addition of α-Methylene-γ-Butyrolactone to Amines

Amines (1.2 mmol) were added to the solution of *α*-methylene-*γ*-butyrolactone **2** or **4** (1.2 mmol) in dry EtOH (2.0 mL). The reaction mixture was stirred at appropriate temperatures for 20–72 h. When the reaction was complete (indicated by TLC), EtOH was removed under reduced pressure. The crude residue was purified by column chromatography on silica gel with an appropriate solvent mixture. The crude products after solvent evaporation were purified as HCl salts by recrystallization in diethyl ether resulting in compounds **5a**–**10** and **13**–**18**.

#### 4.3.1. (3R,3aS,6R,7aR)-3-((Benzylamino)methyl)-6-methylhexahydrobenzofuran-2(3*H*)-one hydrochloride (**5a**)

Prepared from **2** with benzylamine at 25 °C for 20 h. Compound **5a** was purified by column chromatography on silica gel (CHCl_3_/MeOH = 19:1). Yield: 65%, white crystals, m.p.: 190–198 °C. ${\left[\alpha \right]}_{D}^{20}$ = −8.0 (c 0.23, MeOH). ^1^H NMR (500 MHz, DMSO-*d*_6_): δ = 0.94–1.00 (1H, m), 0.95 (3H, d, *J* = 8.1 Hz), 1.09–1.18 (1H, m), 1.24–1.32 (1H, m), 1.59–1.69 (2H, m), 1.78–1.86 (1H, m), 2.06–2.13 (2H, m), 3.05–3.19 (3H, m), 3.93 (1H, td, *J* = 4.4, 14.1 Hz), 7.41–7.63 (5H, m), 9.67 (2H, s). ^13^C NMR (125 MHz, DMSO-*d*_6_): δ = 21.9, 26.1, 30.5, 33.4, 37.6, 42.7, 44.6, 47.0, 50.5, 81.9, 128.5, 128.8, 130.2, 131.7, 176.1. Anal. Calcd for C_17_H_24_ClNO_2_: C, 65.90; H, 7.81; N, 4.52. Found: C, 65.85; H, 7.85; N, 4.52. 

#### 4.3.2. (3R,3aS,6R,7aR)-6-Methyl-3-((((R)-1-phenylethyl)amino)methyl)hexahydrobenzofuran-2(3*H*)-one hydrochloride (**6**)

Prepared from **2** with (*R*)-*α*-methylbenzylamine at 25 °C for 20 h. Compound **6** was purified by column chromatography on silica gel (CHCl_3_/MeOH = 19:1). Yield: 75%, white crystals, m.p.: 170–180 °C. ${\left[\alpha \right]}_{D}^{20}$ = +21.0 (c 0.23, MeOH). ^1^H NMR (500 MHz, DMSO-*d*_6_): δ = 0.91–1.00 (1H, m), 0.95 (3H, d, *J* = 6.5 Hz), 1.08–1.15 (1H, m), 1.23–1.28 (1H, m), 1.53–1.62 (1H, m), 1.61 (3H, d, *J* = 6.4 Hz), 1.68 (1H, d, *J =* 13.2 Hz), 1.75–1.82 (1H, m), 2.03–2.12 (2H, m), 2.78–2.86 (1H, m), 2.94 (1H, t, *J* = 6.3 Hz), 3.05–3.15 (1H, m), 3.92 (1H, td, *J* = 3.4, 11.3 Hz), 4.38 (1H, s), 7.39–7.61 (5H, m), 9.17 (1H, br s), 9.83 (1H, br s). ^13^C NMR (125 MHz, DMSO-*d*_6_): δ = 19.4, 21.9, 26.0, 30.5, 33.4, 37.5, 46.4, 58.2, 81.9, 128.0, 128.8, 176.0. Anal. Calcd for C_18_H_26_ClNO_2_: C, 66.76; H, 8.09; N, 4.32. Found: C, 66.75; H, 8.04; N, 4.30.

#### 4.3.3. (3R,3aS,6R,7aR)-6-Methyl-3-((((S)-1-phenylethyl)amino)methyl)hexahydrobenzofuran-2(3*H*)-one hydrochloride (**7**)

Prepared from **2** with (*S*)-*α*-methylbenzylamine at 25 °C for 20 h. Compound **7** was purified by column chromatography on silica gel (CHCl_3_/MeOH = 19:1). Yield: 71%, white crystals, m.p.: 170–180 °C. ${\left[\alpha \right]}_{D}^{20}$ = −26.0 (c 0.24, MeOH). ^1^H NMR (500 MHz, DMSO-*d*_6_): δ = 0.91–0.97 (1H, m), 0.94 (1H, d, *J* = 6.6 Hz), 1.08–1.15 (1H, m), 1.19–1.28 (1H, m), 1.57–1.67 (2H, m), 1.62 (3H, d, *J* = 6.7 Hz), 1.74–1.81 (1H, m), 1.90–1.93 (1H, m), 2.09–2.11 (1H, m), 2.85–2.94 (1H, m), 2.95–3.02 (1H, m), 3.93 (1H, td, *J =* 3.5, 11.3 Hz), 4.36 (1H, br s), 7.39–7.64 (5H, m), 9.25 (1H, br s), 9.82 (1H, br s). ^13^C NMR (125 MHz, DMSO-*d*_6_): δ = 19.4, 21.9, 26.0, 30.5, 33.4, 37.5, 42.8, 43.5, 46.8, 58.3, 82.0, 127.9, 128.9, 137.1, 176.3. Anal. Calcd for C_14_H_26_ClNO_2_: C, 66.76; H, 8.09; N, 4.32. Found: C, 66.78; H, 8.10; N, 4.35.

#### 4.3.4. (3R,3aS,6R,7aR)-3-((Diethylamino)methyl)-6-methylhexahydrobenzofuran-2(3*H*)-one hydrochloride (**8**)

Prepared from **2** with diethylamine at 25 °C for 20 h. Compound **8** was purified by column chromatography on silica gel (CHCl_3_/MeOH = 19:1). Yield: 50%, colorless oil. ${\left[\alpha \right]}_{D}^{20}$ = −7.0 (c 0.27, MeOH). ^1^H NMR (500 MHz, DMSO-*d*_6_): δ = 0.91–0.99 (1H, m), 0.96 (3H, d, *J* = 6.6 Hz), 1.05 (1H, t, *J* = 7.0 Hz), 1.14 (1H, q, *J* = 11.5 Hz), 1.24 (6H, td, *J* = 2.3, 7.1 Hz), 1.31–1.39 (1H, m), 1.58–1.63 (1H, m), 1.71–1.82 (2H, m), 1.97–2.01 (1H, m), 2.12–2.15 (1H, m), 3.12–3.23 (6H, m), 3.38–3.44 (2H, m), 3.97 (1H, td, *J* = 3.6, 11.4 Hz), 9.94 (1H, br s). ^13^C NMR (125 MHz, DMSO-*d*_6_): δ = 8.4, 8.5, 21.8, 25.6, 30.6, 33.4, 37.5, 41.3, 46.8, 47.2, 47.5, 49.1, 81.9, 176.8. Anal. Calcd for C_14_H_26_ClNO_2_: C, 60.96; H, 9.50; N, 5.08. Found: C, 60.70; H, 9.45; N, 5.10.

#### 4.3.5. (3R,3aS,6R,7aR)-6-Methyl-3-(piperidin-1-ylmethyl)hexahydrobenzofuran-2(3*H*)-one hydrochloride (**9**)

Prepared from **2** with pyridine at 25 °C for 20 h. Compound **9** was purified by column chromatography on silica gel (CHCl_3_/MeOH = 19:1). Yield: 47%, white crystals, m.p.: 180–190 °C. ${\left[\alpha \right]}_{D}^{20}$ = −3.3 (c 0.31, MeOH). ^1^H NMR (500 MHz, DMSO-*d*_6_): δ = 0.92–1.00 (1H, m), 0.96 (1H, d, *J* = 6.6 Hz), 1.14 (1H, q, *J* = 11.5 Hz), 1.30–1.40 (1H, m), 1.58–1.64 (1H, m), 1.67–1.83 (6H, m), 2.02–2.05 (1H, m), 2.11–2.13 (1H, m), 2.88–2.96 (2H, m), 3.16–3.21 (2H, m), 3.26–3.29 (1H, m), 3.42 (1H, d, *J* = 12.0 Hz), 3.94 (1H, td, *J* = 3.6, 11.3 Hz), 10.5 (1H, br s). ^13^C NMR (125 MHz, DMSO-*d*_6_): δ = 21.1, 21.8, 22.2, 25.8, 30.6, 33.4, 37.5, 41.4, 47.7, 51.7, 52.8, 53.8, 81.8, 176.7. Anal. Calcd for C_15_H_26_ClNO_2_: C, 62.59; H, 9.10; N, 4.87. Found: C, 62.60; H, 9.15; N, 4.90.

#### 4.3.6. (3R,3aS,6R,7aR)-3-((Dibenzylamino)methyl)-6-methylhexahydrobenzofuran-2(3*H*)-one hydrochloride (**10**)

Prepared from **2** with dibenzylamine at 70 °C for 72 h. Compound **10** was purified by column chromatography on silica gel (*n*-hexane/ethyl acetate = 9:1). Yield: 59%, white crystals, m.p.: 120–125 °C. ${\left[\alpha \right]}_{D}^{20}$ = −27.0 (c 0.27, MeOH). ^1^H NMR (500 MHz, DMSO-*d*_6_): δ = 0.80–0.87 (1H, m), 0.93 (3H, d, *J* = 6.5 Hz), 1.10 (1H, q, *J* = 11.4 Hz), 1.23–1.31 (1H, m), 1.55–1.72 (4H, m), 2.09 (1H, d, *J* = 11.0 Hz), 3.09 (1H, d, *J* = 12.9 Hz), 3.33–3.40 (1H, m), 3.92 (1H, t, *J* = 9.6 Hz), 4.30–4.33 (2H, m), 4.44 (1H, d, *J* = 9.3 Hz), 7.75–7.70 (10H, m), 11.1 (1H, br s). ^13^C NMR (125 MHz, DMSO-*d*_6_): δ = 21.8, 25.6, 30.5, 33.4, 37.4, 41.6, 47.4, 49.8, 56.7, 56.8, 82.0, 128.8, 129.6, 131.3, 131.4, 131.6, 176.9 Anal. Calcd for C_24_H_30_ClNO_2_: C, 72.07; H, 7.56; N, 3.50. Found: C, 72.07; H, 7.53; N, 3.55.

#### 4.3.7. (3S,3aS,6R,7aS)-3-((Benzylamino)methyl)-6-methylhexahydrobenzofuran-2(3*H*)-one hydrochloride (**13**)

Prepared from **4** with benzylamine at 25 °C for 20 h. Compound **13** was purified by column chromatography on silica gel (CHCl_3_/MeOH = 19:1). Yield: 60%, white crystals, m.p.: 165–167 °C. ${\left[\alpha \right]}_{D}^{20}$ = −34.0 (c 0.24, MeOH). ^1^H NMR (500 MHz, DMSO-*d*_6_): δ = 0.73–0.88 (2H, m), 0.88 (1H, d, *J* = 6.2 Hz), 1.24–1.30 (1H, m), 1.34–1.43 (1H, m), 1.58 (1H, d, *J* = 12.4 Hz), 1.81 (1H, t, *J* = 6.2 Hz), 2.06 (1H, d, *J* = 14.3 Hz), 2.56–2.59 (1H, m), 3.00 (1H, t, *J* = 10.7 Hz), 3.16 (1H, d, *J =* 12.0 Hz), 2.42–2.43 (1H, m), 4.20 (1H, q, *J =* 13.0 Hz), 4.58 (1H, s), 7.42–7.60 (5H, m), 9.54 (2H, s). ^13^C NMR (125 MHz, DMSO-*d*_6_): δ = 21.7, 22.4, 25.8, 31.1, 35.0, 36.2, 41.6, 44.6, 50.2, 78.1, 128.6, 129.0, 130.2, 131.8, 175.6. Anal. Calcd for C_17_H_24_ClNO_2_: C, 65.90; H, 7.81; N, 4.52. Found: C, 65.95; H, 7.80; N, 4.55.

#### 4.3.8. (3S,3aS,6R,7aS)-6-Methyl-3-((((R)-1-phenylethyl)amino)methyl)hexahydrobenzofuran-2(3H)-one hydrochloride (**14**)

Prepared from **4** with (*R*)-*α*-methylbenzylamine at 25 °C for 20 h. Compound **14** was purified by column chromatography on silica gel (CHCl_3_/MeOH = 19:1). Yield: 65%, white crystals, m.p.: 250–252 °C. ${\left[\alpha \right]}_{D}^{20}$ = −3.0 (c 0.24, MeOH). ^1^H NMR (500 MHz, DMSO-*d*_6_): δ = 0.72–0.78 (1H, m), 0.83–0.90 (1H, m), 0.87 (3H, d, *J* = 6.4 Hz), 1.24–1.30 (1H, m), 1.33–1.37 (1H, m), 1.57 (1H, d, *J* = 12.7 Hz), 1.63 (1H, d, *J* = 6.7 Hz), 1.82–1.85 (1H, m), 2.04 (H, d, *J* = 14.1 Hz), 2.61–2.66 (1H, m), 2.79–2.84 (1H, m), 2.91–2.97 (1H, m), 3.29–3.31 (1H, m), 4.44 (1H, q, *J* = 4.8 Hz), 4.55 (1H, d, *J* = 2.3 Hz), 7.40–7.64 (5H, m), 9.49 (1H, br s), 9.94 (1H, br s). ^13^C NMR (125 MHz, DMSO-*d*_6_): δ = 19.3, 21.7, 22.4, 25.8, 31.1, 35.0, 36.3, 40.4, 44.8, 57.7, 78.1, 127.9, 128.9, 137.0, 175.6. Anal. Calcd for C_18_H_26_ClNO_2_: C, 66.76; H, 8.09; N, 4.32. Found: C, 66.74; H, 8.13; N, 4.35.

#### 4.3.9. (3S,3aS,6R,7aS)-6-Methyl-3-((((S)-1-phenylethyl)amino)methyl)hexahydrobenzofuran-2(3*H*)-one hydrochloride (**15**)

Prepared from **4** with (*S*)-*α*-methylbenzylamine at 25 °C for 20 h. Compound **15** was purified by column chromatography on silica gel (CHCl_3_/MeOH = 19:1). Yield: 70%, white crystals, m.p.: 230–235 °C. ${\left[\alpha \right]}_{D}^{20}$ = −57.0 (c 0.21, MeOH). ^1^H NMR (500 MHz, DMSO-*d*_6_): δ = 0.55–0.63 (1H, m), 0.78–0.85 (1H, m), 0.85 (3H, d, *J* = 6.2 Hz), 1.22–1.30 (2H, m), 1.50 (2H, d, *J* = 10.9 Hz), 1.64 (1H, d, *J* = 6.7 Hz), 2.03 (1H, d, *J =* 13.4 Hz), 2.50–2.56 (1H, m), 2.59–2.63 (1H, m), 3.05 (1H, t, *J* = 9.7 Hz), 3.42–3.44 (1H, m), 4.47 (1H, br s), 4.57 (1H, d, *J* = 2.1 Hz), 7.39–7.64 (5H, m), 9.48 (1H, d, *J* = 7.6 Hz), 10.04 (1H, br s). ^13^C NMR (125 MHz, DMSO-*d*_6_): δ = 19.5, 21.7, 22.1, 25.7, 31.0, 35.0, 36.0, 40.2, 44.5, 57.6, 78.0, 127.8, 128.9, 129.0, 136.9, 175.4. Anal. Calcd for C_18_H_26_ClNO_2_: C, 66.76; H, 8.09; N, 4.32. Found: C, 66.77; H, 8.13; N, 4.29.

#### 4.3.10. (3S,3aS,6R,7aS)-3-((Diethylamino)methyl)-6-methylhexahydrobenzofuran-2(3*H*)-one hydrochloride (**16**)

Prepared from **4** with diethylamine at 25 °C for 20 h. Compound **16** was purified by column chromatography on silica gel (CHCl_3_/MeOH = 19:1). Yield: 50%, white crystals, m.p.: 158–163 °C. ${\left[\alpha \right]}_{D}^{20}$ = −39.0 (c 0.24, MeOH). ^1^H NMR (500 MHz, DMSO-*d*_6_): δ = 0.81–0.93 (1H, m), 0.87 (1H, d, *J* = 9.0 Hz), 1.20–1.26 (6H, m), 1.26–1.29 (1H, m), 1.33–1.43 (1H, m), 1.60 (1H, d, *J* = 11.5 Hz), 1.82–1.85 (1H, m), 2.07 (1H, d, *J* = 14.6 Hz), 2.64–2.66 (1H, m), 3.14–3.22 (6H, m), 3.58 (1H, q, *J* = 4.2 Hz), 4.58 (1H, d, *J* = 2.2 Hz), 10.6 (1H, br s). ^13^C NMR (125 MHz, DMSO-*d*_6_): δ = 8.0, 8.6, 21.7, 22.6, 25.8, 31.1, 35.1, 37.1, 42.9, 45.9, 46.4, 47.1, 78.0, 176.1. Anal. Calcd for C_14_H_26_ClNO_2_: C, 60.96; H, 9.50; N, 5.08. Found: C, 60.73; H, 9.53; N, 5.05.

#### 4.3.11. (3S,3aS,6R,7aS)-6-Methyl-3-(piperidin-1-ylmethyl)hexahydrobenzofuran-2(3*H*)-one hydrochloride (**17**)

Prepared from **4** with pyridine at 25 °C for 20 h. Compound **17** was purified by column chromatography on silica gel (CHCl_3_/MeOH = 19:1). Yield: 53%, white crystals, m.p.: 231–233 °C. ${\left[\alpha \right]}_{D}^{20}$ = −39.0 (c 0.24, MeOH). ^1^H NMR (500 MHz, DMSO-*d*_6_): δ = 0.76–0.81 (1H, m), 0.87–0.92 (1H, m), 0.88 (3H, d, *J* = 6.0 Hz), 1.22–1.28 (1H, m), 1.31–1.44 (2H, m), 1.59–1.89 (7H, m), 2.07 (1H, d, *J* = 14.5 Hz), 2.65 (1H, d, *J* = 5.6 Hz), 2.87–2.97 (2H, m), 3.17–3.22 (2H, m), 3.41 (1H, d, *J* = 10.9 Hz), 3.52 (1H, d, *J* = 11.1 Hz), 3.61 (1H, br s), 4.56 (1H, br s), 10.6 (1H, br s) ^13^C NMR (125 MHz, DMSO-*d*_6_): δ = 21.2, 21.7, 22.1, 22.2, 22.8, 25.8, 31.1, 35.1, 37.3, 43.2, 51.2, 51.7, 52.6, 77.9, 176.0. Anal. Calcd for C_15_H_26_ClNO_2_: C, 62.59; H, 9.10; N, 4.87. Found: C, 62.57; H, 9.05; N, 4.93.

#### 4.3.12. (3S,3aS,6R,7aS)-3-((Dibenzylamino)methyl)-6-methylhexahydrobenzofuran-2(3*H*)-one hydrochloride (**18**)

Prepared from **4** with dibenzylamine at 70 °C for 72 h. Compound **18** was purified by column chromatography on silica gel (*n*-hexane/ethyl acetate = 9:1). Yield: 50%, white crystals, m.p.: 118–120 °C. ${\left[\alpha \right]}_{D}^{20}$ = −23.0 (c 0.21, MeOH). ^1^H NMR (500 MHz, DMSO-*d*_6_): δ = 0.45–0.52 (1H, m), 0.73–0.85 (1H, m), 0.83 (1H, d, *J* = 6.1 Hz), 1.17–1.30 (3H, m), 1.41 (1H, d, *J* = 12.4 Hz), 2.03 (1H, d, *J* = 13.7 Hz), 2.70 (1H, d, *J* = 4.9 Hz), 2.80 (1H, t, *J* = 10.2 Hz), 3.20–3.24 (1H, m), 3.78 (1H, br s), 4.35–4.40 (2H, m), 4.49–4.53 (2H, m), 7.33–7.70 (10H, m), 11.3 (1H, br s). ^13^C NMR (125 MHz, DMSO-*d*_6_): δ = 21.6, 22.1, 25.6, 31.0, 35.0, 36.9, 42.8, 46.0, 56.1, 56.6, 77.9, 128.8, 129.1, 129.6, 129.7, 129.9, 131.6, 131.9, 175.6. Anal. Calcd for C_24_H_30_ClNO_2_: C, 72.07; H, 7.56; N, 3.50. Found: C, 72.09; H, 7.53; N, 3.55.

### 4.4. General Procedure for Nucleophilic Addition of α-Methylene-γ-Butyrolactone with Amino Esters

To the solution of *α*-methylene-*γ*-butyrolactone **2** or **4** (1.2 mmol) in dry EtOH (2.0 mL) was added l- or *β*-alanine ethyl ester hydrochloride (2.4 mmol) and Et_3_N (2.4 mmol). The reaction mixture was stirred at the appropriate temperature for 20 h. When the reaction was complete (indicated by TLC), EtOH was removed under reduced pressure. The crude residue was purified by column chromatography on silica gel with a mixture of CHCl_3_ and MeOH (19:1). After solvent evaporation, the addition of a few drops of HCl/EtOH, and recrystallization in diethyl ether, compounds **11** and **12,** as well as **19** and **20**, respectively, were isolated.

#### 4.4.1. Ethyl 3-(((3R,3aS,6R,7aR)-6-methyl-2-oxooctahydrobenzofuran-3-yl)methylamino) propanoate hydrochloride (**11**)

Prepared from **2** with *β*-alanine ethyl ester hydrochloride at 25 °C. Yield: 60%, white crystals, m.p.: 125–135 °C. ${\left[\alpha \right]}_{D}^{20}$ = −3.7 (c 0.32, MeOH). ^1^H NMR (500 MHz, CDCl_3_): δ = 1.00–1.07 (1H, m), 1.02 (3H, d, *J* = 6.6 Hz), 1.20–1.30 (1H, m), 1.29 (3H, t, *J* = 7.1 Hz), 1.44–1.50 (1H, m), 1.64–1.72 (2H, m), 1.82 (1H, d, *J =* 13.6 Hz), 2.01 (1H, d, *J =* 10.7 Hz), 2.26 (1H, m), 2.89 (1H, dt, *J* = 4.3, 18.0 Hz), 3.10–3.38 (6H, m), 4.00 (1H, td, *J* = 3.6, 11.3 Hz), 4.22 (2H, q, *J* = 7.1 Hz), 8.70 (1H, br s), 11.30 (1H, br s). ^13^C NMR (125 MHz, CDCl_3_): δ = 14.2, 22.0, 26.2, 30.2, 31.2, 33.8, 38.1, 42.8, 44.6, 47.2, 48.3, 62.2, 84.0, 171.8, 178.3. Anal. Calcd for C_15_H_26_ClNO_4_: C, 56.33; H, 8.19; N, 4.38. Found: C, 56.35; H, 8.15; N, 4.40.

#### 4.4.2. (S)-Ethyl 2-(((3R,3aS,6R,7aR)-6-methyl-2-oxooctahydrobenzofuran-3-yl)methylamino)propanoate hydrochloride (**12**)

Prepared from **2** with L-alanine ethyl ester hydrochloride at 25 °C. Yield: 40%, white crystals, m.p.: 150–160 °C. ${\left[\alpha \right]}_{D}^{20}$ = −3.0 (c 0.28, MeOH). ^1^H NMR (500 MHz, CDCl_3_): δ = 0.97–1.02 (1H, m), 1.02 (3H, d, *J* = 6.5 Hz), 1.22 (1H, q, *J* = 11.4 Hz), 1.33 (3H, t, *J* = 7.1 Hz), 1.49 (1H, q, *J* = 11.9 Hz), 1.62–1.66 (2H, m), 1.82 (6H, d, *J* = 6H, d, *J* = 6.7 Hz), 1.98 (1H, d, *J* = 12.4 Hz), 2.27 (1H, d, *J* = 11.5 Hz), 3.11 (1H, t, *J* = 10.0 Hz), 3.48–3.48 (1H, m), 3.54 (1H, t, *J* = 10.0 Hz), 3.96 (1H, q, *J* = 6.0 Hz), 4.03 (1H, td, *J* = 3.1, 11.3 Hz), 4.27–4.35 (2H, m), 8.14 (1H, br s), 12.2 (1H, br s). ^13^C NMR (125 MHz, CDCl_3_): δ = 14.1, 15.9, 22.0, 26.2, 31.1, 33.9, 38.1, 42.6, 46.3, 48.5, 60.0, 63.3, 83.9, 169.2, 178.6. Anal. Calcd for C_15_H_26_ClNO_4_: C, 56.33; H, 8.19; N, 4.38. Found: C, 56.30; H, 8.20; N, 4.35.

#### 4.4.3. Ethyl 3-(((3S,3aS,6R,7aS)-6-methyl-2-oxooctahydrobenzofuran-3-yl) methylamino)propanoate hydrochloride (**19**)

Prepared from **4** with *β*-alanine ethyl ester hydrochloride at 70 °C. Yield: 60%, white crystals, m.p.: 202–205 °C. ${\left[\alpha \right]}_{D}^{20}$ = −37.0 (c 0.21, MeOH). ^1^H NMR (500 MHz, CDCl_3_): δ = 0.93 (3H, d, *J* = 6.4 Hz), 0.92–0.99 (1H, m), 1.09 (1H, q, *J* = 10.0 Hz), 1.24–1.30 (1H, m), 1.29 (3H, t, *J* = 6.9 Hz), 1.49–1.62 (1H, m), 1.70 (1H, d, *J* = 12.3 Hz), 1.78 (1H, d, *J* = 9.5 Hz), 2.24 (1H, d, *J* = 14.7 Hz), 2.66 (1H, br s), 2.92 (1H, d, *J* = 16.7 Hz), 3.13 (1H, d, *J* = 16.2 Hz), 3.26–3.30 (4H, m), 3.60–3.70 (1H, m), 4.22 (2H, q, *J* = 7.0 Hz), 4.65 (1H, s), 8.81 (1H, br s), 10.8 (1H, br s). ^13^C NMR (125 MHz, CDCl_3_): δ = 14.2, 21.9, 23.3, 26.1, 30.4, 31.5, 35.6, 37.7, 44.4, 44.5, 44.7, 62.0, 79.7, 171.1, 177.7. Anal. Calcd for C_15_H_26_ClNO_4_: C, 56.33; H, 8.19; N, 4.38. Found: C, 56.37; H, 8.18; N, 4.35.

#### 4.4.4. (S)-Ethyl 2-(((3R,3aS,6R,7aR)-6-methyl-2-oxooctahydrobenzofuran-3-yl)methylamino)propanoate hydrochloride (**20**)

Prepared from **4** with L-alanine ethyl ester hydrochloride at 70 °C. Yield: 44%, white crystals, m.p.: 216–218 °C. ${\left[\alpha \right]}_{D}^{20}$ = −35.0 (c 0.24, MeOH). ^1^H NMR (500 MHz, CDCl_3_): δ = 0.92 (3H, d, *J* = 6.5 Hz), 0.94–1.02 (2H, m), 1.24 (1H, t, *J* = 12.3 Hz), 1.33 (3H, t, *J* = 7.0 Hz), 1.50–1.60 (1H, m), 1.70 (1H, d, *J* = 11.5 Hz), 1.76 (3H, d, *J* = 6.9 Hz), 1.93 (1H, d, *J* = 10.6 Hz), 2.00–2.14 (1H, m), 2.23 (1H, d, *J* = 14.9 Hz), 2.78–2.85 (1H, m), 3.32 (1H, d, *J* = 17.2 Hz), 3.77 (1H, d, *J* = 4.5 Hz), 4.08–4.16 (1H, m), 4.30 (2H, q, *J* = 7.0 Hz), 4.61 (1H, s), 9.80 (1H, br s), 10.37 (1H, br s). ^13^C NMR (125 MHz, CDCl_3_): δ = 14.2, 15.0, 22.0, 23.1, 26.1, 31.6, 35.8, 37.7, 41.9, 45.2, 55.8, 63.1, 79.5, 168.7, 176.8. Anal. Calcd for C_15_H_26_ClNO_4_: C, 56.33; H, 8.19; N, 4.38. Found: C, 56.35; H, 8.17; N, 4.40.

### 4.5. General Procedure for the Preparation of β-Aminoamides

To a solution of *α*-methylene-*γ*-butyrolactone, **2** (1.2 mmol) or *β*-aminolactones **13**–**15** (1.2 mmol) in an appropriate solvent (2.0 mL) was added a solution of the appropriate amine (4.8 mmol). The mixture was stirred at the appropriate temperature for 20–72 h. When the reaction was complete (indicated by TLC), the mixture was evaporated to dryness. The crude product was purified by column chromatography on silica gel with CHCl_3_/MeOH (19:1), resulting in compounds **21**–**23** and **32**–**34**. 

#### 4.5.1. (R)-*N*-Benzyl-3-(benzylamino)-2-((1S,2R,4R)-2-hydroxy-4-methylcyclohexyl)propanamide (**21**)

Prepared from **2** with benzylamine at 25 °C for 20 h in dry EtOH. Yield: 90%, white crystals, m.p.: 185–195 °C. ${\left[\alpha \right]}_{D}^{20}$ = −24.0 (c 0.27, MeOH). ^1^H NMR (500 MHz, DMSO-*d*_6_): δ = 0.66–0.73 (1H, m), 0.82–0.92 (2H, m), 0.85 (1H, d, *J* = 6.5 Hz), 1.34–1.37 (2H, m), 1.52 (1H, d, *J* = 12.2 Hz), 1.65 (1H, t, *J* = 11.1 Hz), 1.86 (1H, d, *J =* 12.4 Hz), 2.93–3.02 (1H, m), 3.24–3.27 (3H, m), 4.06–4.17 (3H, m), 4.44 (1H, dd, *J* = 6.4, 15.1 Hz), 5.02–5.09 (1H, m), 7.20–7.54 (10H, m), 8.66–8.68 (2H, m), 9.28 (1H, br s). ^13^C NMR (125 MHz, DMSO-*d*_6_): δ = 22.1, 25.1, 30.9, 33.9, 41.7, 42.3, 43.3, 44.4, 47.2, 50.4, 68.5, 126.7, 127.1, 128.2, 128.6, 128.9, 130.0, 131.8, 139.5, 172.0. Anal. Calcd for C_24_H_32_N_2_O_2_: C, 75.75; H, 8.48; N, 7.36. Found: C, 75.80; H, 8.45; N, 7.35.

#### 4.5.2. (R)-2-((1S,2R,4R)-2-Hydroxy-4-methylcyclohexyl)-*N*-((R)-1-phenylethyl)-3-(((R)-1-phenylethyl)amino)propanamide (**22**)

Prepared from **2** with (*R*)-*α*-methylbenzylamine at 70 °C for 48 h in dry EtOH. Yield: 58%, colorless oil. ${\left[\alpha \right]}_{D}^{20}$ = +54.0 (c 0.20, MeOH). ^1^H NMR (500 MHz, CDCl_3_): δ = 0.72–0.90 (2H, m), 0.89 (1H, 6.4 Hz), 1.04–1.11 (1H, m), 1.25–1.28 (3H, m), 1.36 (3H, d, *J* = 6.6 Hz), 1.47–1.62 (2H, m), 1.53 (3H, d, *J* = 6.9 Hz), 1.78–1.87 (2H, m), 1.96 (1H, 1H, *J* = 13.6 Hz), 2.70 (1H, d, *J* = 11.8 Hz), 3.00–3.06 (2H, m), 3.42 (1H, d, *J* = 8.7 Hz), 3.62–3.70 (1H, m), 5.02 (1H, t, *J* = 7.1 Hz), 7.20–7.49 (10H, m), 8.38 (1H, d, *J* = 7.1 Hz). ^13^C NMR (125 MHz, CDCl_3_): δ = 20.9, 22.0, 22.2, 25.8, 31.7, 34.1, 42.3, 43.5, 44.4, 47.7, 49.8, 58.7, 70.8, 126.7, 127.1, 127.6, 128.5, 128.9, 129.1, 144.4, 172.4. Anal. Calcd for C_26_H_36_N_2_O_2_: C, 76.43; H, 8.88; N, 6.86. Found: C, 76.45; H, 8.90; N, 6.83.

#### 4.5.3. (R)-2-((1S,2R,4R)-2-Hydroxy-4-methylcyclohexyl)-*N*-((S)-1-phenylethyl)-3-(((S)-1-phenylethyl)amino)propanamide (**23**)

Prepared from **2** with (*S*)-*α*-methylbenzylamine at 70 °C for 48 h in dry EtOH. Yield: 54%, white crystals, m.p.: 137–148 °C ${\left[\alpha \right]}_{D}^{20}$= −55.0 (c 0.26, MeOH). ^1^H NMR (500 MHz, DMSO-*d*_6_): δ = 0.52–0.58 (1H, m), 0.82 (3H, d, *J* = 6.4 Hz), 0.86–0.91 (2H, m), 1.23–1.37 (2H, m), 1.36 (3H, d, *J* = 7.1 Hz), 1.56 (3H, d, *J* = 6.8 Hz), 1.69–1.74 (1H, m), 1.84–1.88 (1H, m), 2.78–2.82 (1H, m), 2.87–2.93 (1H, m), 3.24 (2H, d, *J* = 10.0 Hz), 4.29 (1H, d, *J* = 2.3 Hz), 4.91 (1H, quin, *J =* 7.1 Hz), 5.10 (1H, d, *J* = 4.9 Hz), 7.15–7.53 (10H, m), 8.30 (1H, d, *J =* 9.0 Hz), 8.64 (1H, d, *J* = 7.7 Hz), 9.60 (1H, br s). ^13^C NMR (125 MHz, DMSO-*d*_6_): δ = 19.8, 22.1, 22.3, 24.5, 30.8, 33.7, 41.3, 41.7, 44.2, 47.2, 48.4, 58.0, 68.4, 125.6, 126.4, 127.6, 128.0, 128.7, 128.8, 137.1, 145.4, 171.0. Anal. Calcd for C_26_H_36_N_2_O_2_: C, 76.43; H, 8.88; N, 6.86. Found: C, 76.40; H, 8.85; N, 6.90.

#### 4.5.4. (S)-*N*-Benzyl-3-(benzylamino)-2-((1S,2S,4R)-2-hydroxy-4-methylcyclohexyl)propanamide (**32**)

Prepared from **13** with benzylamine at 70 °C for 24 h in dry THF. Yield: 70%, white crystals, m.p.: 251–253 °C. ${\left[\alpha \right]}_{D}^{20}$ = +21.0 (c = 0.20, MeOH). ^1^H NMR (500 MHz, DMSO-*d*_6_): δ = 0.76–0.80 (1H, m), 0.79 (3H, d, *J* = 6.3 Hz), 1.00 (1H, t, *J* = 13.0 Hz), 1.18 (1H, d, *J* = 10.1 Hz), 1.38–1.43 (1H, m), 1.51–1.58 (2H, m), 1.68 (1H, d, *J* = 11.2 Hz), 2.75 (1H, t, *J* = 6.7 Hz), 3.05 (1H, d, *J* = 11.8 Hz), 3.12–3.22 (1H, m), 3.79 (1H, s), 4.11 (2H, s), 4.18 (1H, dd, *J* = 5.2, 14.9 Hz), 4.29 (1H, dd, *J* = 6.1, 14.9 Hz), 4.74 (1H, s), 7.22–7.54 (10H, m), 8.75 (1H, t, *J* = 5.6 Hz), 9.06 (1H, br s), 9.17 (1H, br s). ^13^C NMR (125 MHz, DMSO-*d*_6_): δ = 22.2, 24.3, 25.1, 34.3, 41.2, 41.8, 42.5, 44.3, 45.3, 50.1, 64.0, 126.8, 127.5, 128.2, 128.6, 128.9, 130.1, 131.7, 139.0, 172.3. Anal. Calcd for C_24_H_32_N_2_O_2_: C, 75.75; H, 8.48; N, 7.36. Found: C, 75.76; H, 8.50; N, 7.32.

#### 4.5.5. (S)-2-((1S,2S,4R)-2-Hydroxy-4-methylcyclohexyl)-*N*-((R)-1-phenylethyl)-3-(((R)-1-phenylethyl)amino)propanamide (**33**)

Prepared from **14** with (*R*)-*α*-methylbenzylamine at 70 °C for 72 h in dry THF. Yield: 42%, white crystals, m.p.: 190–192 °C. ${\left[\alpha \right]}_{D}^{20}$ = +46.0 (c 0.21, MeOH). ^1^H NMR (500 MHz, CDCl_3_): δ = 0.64–0.72 (1H, m), 0.79 (3H, 6.5 Hz), 0.99 (1H, t, *J* = 12.5 Hz), 1.14 (1H, d, *J* = 9.8 Hz), 1.39–1.54 (3H, m), 1.62–1.64 (1H, m), 1.77–1.81 (1H, m), 1.78 (3H, d, *J* = 6.7 Hz), 2.96 (2H, d, *J* = 8.6 Hz), 3.27 (1H, d, *J* = 8.9 Hz), 3.97 (1H, s), 4.36 (1H, q, *J* = 7.3 Hz), 4.80 (1H, quin, *J* = 7.3 Hz), 7.17–7.52 (10H, m), 8.23 (1H, d, *J* = 7.7 Hz) . ^13^C NMR (125 MHz, CDCl_3_): δ = 20.2, 22.3, 25.1, 26.0, 34.3, 42.1, 42.6, 45.8, 46.1, 49.7, 59.8, 65.7, 126.6, 127.1, 127.6, 128.5, 129.6, 129.7, 135.7, 143.8, 171.6. Anal. Calcd for C_26_H_36_N_2_O_2_: C, 76.43; H, 8.88; N, 6.86. Found: C, 76.41; H, 8.85; N, 6.90.

#### 4.5.6. (S)-2-((1S,2S,4R)-2-Hydroxy-4-methylcyclohexyl)-*N*-((S)-1-phenylethyl)-3-(((S)-1-phenylethyl)amino)propanamide (**34**)

Prepared by **15** with (*S*)-*α*-methylbenzylamine at 70 °C for 72 h in dry THF. Yield: 45%, colorless oil. ${\left[\alpha \right]}_{D}^{20}$ = −36.0 (c 0.23, MeOH). ^1^H NMR (500 MHz, CDCl_3_): δ = 0.84 (3H, d, *J* = 6.3 Hz), 0.83–0.89 (2H, m), 1.01 (1H, t, *J* = 12.5 Hz), 1.22–1.29 (3H, m), 1.37–1.43 (1H, m), 1.42 (3H, d, *J* = 6.6 Hz), 1.49 (3H, d, *J* = 6.9 Hz), 1.51–1.57 (2H, m), 1.66–1.80 (4H, m), 2.61 (1H, br s), 2.77 (2H, d, *J* = 5.0 Hz), 3.70 (1H, t, *J* = 6.9 Hz), 3.77 (1H, s), 5.08 (1H, d, *J* = 7.1 Hz), 7.23–7.41 (10H, m), 7.65 (1H, br s). ^13^C NMR (125 MHz, CDCl_3_): δ = 22.0, 22.1, 22.3, 25.2, 25.9, 29.8, 34.7, 42.0, 42.8, 46.5, 47.8, 49.2, 58.9, 66.3, 126.3, 126.5, 127.3, 128.5, 128.7, 128.8, 129.0, 143.7, 172.9. Anal. Calcd for C_26_H_36_N_2_O_2_: C, 76.43; H, 8.88; N, 6.86. Found: C, 76.45; H, 8.83; N, 6.87.

### 4.6. General Procedure for the Hydrolysis of β-Aminoamides

The solution of *β*-aminoamides **21**–**23** or **32**–**34** (0.5 mmol) in EtOH (2 mL) mixed with 10% aqueous HCl (10 mL) was stirred at room temperature. After completion of the reaction (as monitored by TLC, 24 h), the mixture was extracted with CH_2_Cl_2_ (3 × 10 mL), dried over Na_2_SO_4_, filtered, and evaporated to dryness. The crude product was purified by recrystallization with diethyl ether, resulting in compounds **5a**–**7** or **13**–**15**, respectively. All spectroscopic data are listed above.

### 4.7. General Procedure for Preparation of Dipeptides

To the solution of *α*-methylene-*γ*-butyrolactone **2** or *N*-benzyl aminolactone **5a** (1.2 mmol) in dry EtOH (2.0 mL) was added L- or *β*-alanine ethyl ester (3.6 mmol). The mixture was stirred at the appropriate temperature for 48 h. When the reaction was complete (monitored by TLC), the mixture was evaporated to dryness, then purified by column chromatography on silica gel (CHCl_3_/MeOH = 19:1), affording compounds **27**–**29**. 

#### 4.7.1. Ethyl 3-((R)-2-((1S,2R,4R)-2-hydroxy-4-methylcyclohexyl)-3-((3-ethoxy-3-oxopropyl)amino)propanemido)propanoate (**27**)

Prepared from **2** with *β*-alanine ethyl ester at 25 °C. Yield: 63%, colorless oil. ${\left[\alpha \right]}_{D}^{20}$ = −14.2 (c 0.33, MeOH). ^1^H NMR (500 MHz, CDCl_3_): δ = 0.81–1.09 (5H, m), 0.90 (3H, d, *J* = 6.2 Hz), 1.25–1.28 (11H, m), 1.33–1.40 (1H, m), 1.60 (2H, t, *J* = 14.6 Hz), 1.72 (1H, t, *J* = 10.2 Hz), 2.00 (1H, d, *J* = 13.2 Hz), 2.59 (2H, t, *J* = 6.3 Hz), 0.83 (1H, d, *J* = 17.6 Hz), 2.98–3.02 (1H, m), 3.18–3.37 (5H, m), 3.49–3.60 (3H, m), 4.14 (2H, q, *J* = 7.2 Hz), 4.19 (2H, q, *J* = 6.4 Hz). ^13^C NMR (125 MHz, CDCl_3_): δ = 14.1, 14.2, 22.0, 25.3, 29.7, 30.3, 31.8, 34.1, 34.2, 35.4, 42.1, 43.9, 44.0, 45.1, 47.8, 60.7, 61.6, 70.0, 170.8, 172.3, 173.2. Anal. Calcd for C_20_H_36_N_2_O_6_: C, 59.98; H, 9.06; N, 6.99. Found: C, 60.00; H, 9.05; N, 6.95.

#### 4.7.2. Ethyl 3-((R)-3-(benzylamino)-2-((1S,2R,4R)-2-hydroxy-4-methylcyclohexyl)propanamido)propanoate (**28**)

Prepared from **5a** with *β*-alanine ethyl ester at 70 °C. Yield: 45%, white crystals, m.p.: 169–173 °C. ${\left[\alpha \right]}_{D}^{20}$ = −24.0 (c 0.24, MeOH). ^1^H NMR (500 MHz, CDCl_3_): δ = 0.78–0.98 (3H, m), 0.85 (3H, d, *J* = 6.4 Hz), 1.24 (3H, t, *J* = 7.1 Hz), 1.23–1.29 (1H, m), 1.58 (2H, d, *J* = 10.6 Hz), 1.71 (2H, t, *J* = 11.5 Hz), 2.57 (2H, t, *J* = 5.7 Hz), 3.22–3.33 (3H, m), 3.42–3.47 (1H, m), 3.52–3.60 (2H, m), 4.08 (3H, q, *J* = 7.1 Hz), 4.31–4.34 (1H, m), 7.40 (3H, 6.0 Hz), 7.57 (2H, d, *J* = 5.3 Hz), 7.86 (1H, br s), 8.18 (1H, t, *J* = 5.5 Hz).10.08 (1H, br s). ^13^C NMR (125 MHz, CDCl_3_): δ = 14.3, 22.0, 25.4, 31.8, 34.0, 34.3, 35.6, 42.3, 43.9, 44.7, 47.8, 52.3, 60.9, 70.2, 129.3, 129.7, 130.1, 130.7, 172.8, 173.4. Anal. Calcd for C_22_H_34_N_2_O_4_: C, 67.66; H, 8.78; N, 7.17. Found: C, 67.70; H, 8.75; N, 7.20.

#### 4.7.3. (S)-Ethyl 2-((R)-3-(benzylamino)-2-((1S,2R,4R)-2-hydroxy-4-methylcyclohexyl)propanamido)propanoate (**29**)

Prepared from **5a** with L-alanine ethyl ester at 70 °C. Yield: 40%, white crystals, m.p.: 115–117 °C. ${\left[\alpha \right]}_{D}^{20}$ = −30.0 (c 0.25, MeOH). ^1^H NMR (500 MHz, CDCl_3_): δ = 0.81–0.89 (2H, m), 0.88 (3H, d, *J* = 6.5 Hz), 1.04 (1H, q, *J* = 11.8 Hz), 1.25 (3H, t, *J* = 7.1 Hz), 1.28–1.38 (1H, m), 1.47 (3H, d, *J* = 7.2 Hz), 1.58–1.66 (2H, m), 1.81–1.90 (2H, m), 1.95–2.17 (2H, m), 3.10–3.18 (1H, m), 3.27–3.34 (2H, m), 3.64 (1H, d, *J* = 10.2 Hz), 4.00–4.03 (1H, m), 4.14–4.18 (3H, m), 4.42 (1H, quin, *J* = 6.9 Hz), 7.39 (3H, d, *J* = 3.6 Hz), 7.51 (2H, d, *J* = 3.8 Hz), 7.99 (1H, d, *J* = 6.1 Hz), 10.1 (br s). ^13^C NMR (125 MHz, CDCl_3_): δ = 14.3, 17.3, 22.1, 25.2, 31.8, 34.1, 42.1, 43.8, 44.2, 48.0, 49.0, 52.3, 52.3, 61.3, 70.0, 129.4, 129.8, 130.1, 130.4, 173.0, 173.2. Anal. Calcd for C_22_H_34_N_2_O_4_: C, 67.66; H, 8.78; N, 7.17. Found: C, 67.65; H, 8.80; N, 7.15.

### 4.8. General Procedure for Debenzylation

To a suspension of 5% Pd/C or Pd(OH)_2_/C (100 mg) in MeOH (10 mL) was added *β*-aminoamides **21**–**23** and **32**–**34** or *N*-benzyldipepetides **28**–**29** (0.38 mmol) in MeOH (10 mL). The mixture was stirred under H_2_ at room temperature and normal pressure. When the reaction was complete (indicated by TLC), the mixture was filtered through a Celite pad, the solution was evaporated to dryness and purified by recrystallization in diethyl ether providing **24**–**26** and **35**–**37** as well as **30**–**31**, respectively.

#### 4.8.1. (R)-3-Amino-N-benzyl-2-((1S,2R,4R)-2-hydroxy-4-methylcyclohexyl)propanamide (**24**)

Prepared from **21** with 5% Pd/C for 96 h. Yield: 80%, white crystals, m.p.: 226–230 °C. ${\left[\alpha \right]}_{D}^{20}$ = −24.0 (c 0.29, MeOH). ^1^H NMR (500 MHz, DMSO-*d*_6_): δ = 0.67–0.74 (1H, m), 0.82–0.97 (2H, m), 0.85 (3H, d, *J* = 6.4 Hz), 1.34–1.37 (2H, m), 1.54 (1H, d, *J* = 12.0 Hz), 1.69 (1H, t, *J* = 12.3 Hz), 1.87 (1H, d, *J* = 11.9 Hz), 2.81 (1H, dd, *J* = 1.9, 10.1 Hz), 3.08 (1H, t, *J* = 11.9 Hz), 3.17 (1H, d, *J* = 10.3 Hz), 3.28 (1H, td, *J* = 3.9, 10.4 Hz), 4.18 (1H, dd, *J* = 5.4, 15.1 Hz), 4.43 (1H, dd, *J* = 6.4, 15.2 Hz), 7.21–7.32 (5H, m), 7.91 (3H, br s), 8.72 (1H, t, *J* = 5.8 Hz). ^13^C NMR (125 MHz, DMSO-*d*_6_): δ = 22.1, 25.0, 30.9, 34.1, 35.5, 42.2, 42.6, 44.7, 46.8, 68.7, 126.7, 127.1, 128.2, 139.6, 172.2. Anal. Calcd for C_17_H_26_N_2_O_2_: C, 70.31; H, 9.02; N, 9.65. Found: C, 70.35; H, 9.05; N, 9.60.

#### 4.8.2. (R)-3-Amino-2-((1S,2R,4R)-2-hydroxy-4-methylcyclohexyl)-*N*-((R)-1-phenylethyl)propanamide (**25**)

Prepared from **22** with 5% Pd/C for 168 h. Yield: 62%, white crystals, m.p.: 145–150 °C. ${\left[\alpha \right]}_{D}^{20}$ = +28.7 (c 0.31, MeOH). ^1^H NMR (500 MHz, DMSO-*d*_6_): δ = 0.73–0.79 (1H, m), 0.87 (3H, d, *J* = 6.4 Hz), 0.90–1.00 (2H, m), 1.34 (3H, d, *J* = 6.9 Hz), 1.37–1.46 (2H, m), 1.59 (1H, d, *J* = 12.2 Hz), 1.81 (1H, t, *J* = 12.3 Hz), 1.88 (1H, d, *J* = 11.9 Hz), 2.81 (1H, dd, *J* = 2.6, 12.1 Hz), 3.05 (1H, d, *J* = 12.0 Hz), 3.14–3.17 (1H, m), 3.28 (1H, td, *J* = 3.7, 10.3 Hz), 5.01 (1H, quin, *J* = 7.4 Hz), 7.20–7.40 (5H, m), 8.65 (1H, d, *J* = 8.3 Hz). ^13^C NMR (125 MHz, DMSO-*d*_6_): δ = 22.1, 22.6, 25.1, 31.0, 34.3, 35.5, 42.5, 44.8, 46.5, 48.0, 68.9, 126.4, 126.6, 128.2, 144.3, 171.4. Anal. Calcd for C_18_H_28_N_2_O_2_: C, 71.02; H, 9.27; N, 9.20. Found: C, 71.05; H, 9.30; N, 9.15.

#### 4.8.3. (R)-3-Amino-2-((1S,2R,4R)-2-hydroxy-4-methylcyclohexyl)-*N*-((S)-1-phenylethyl)propanamide (**26**)

Prepared from **23** (0.16 g, 0.39 mmol) with Pd(OH)_2_/C for 200 h. Yield: 65%, white crystals, m.p.: 150–160 °C. ${\left[\alpha \right]}_{D}^{20}$ = −37.4 (c 0.31, MeOH). ^1^H NMR (500 MHz, DMSO-*d*_6_): δ = 0.59–0.66 (1H, m), 0.74–0.82 (1H, m), 0.84 (3H, d, *J* = 6.4 Hz), 0.88–0.97 (1H, m), 1.06 (1H, d, *J* = 10.8 Hz), 1.22–1.26 (1H, m), 1.30–1.37 (1H, m), 1.36 (3H, d, *J* = 7.0 Hz), 1.42 (1H, d, *J* = 12.1 Hz), 1.77–1.87 (2H, m), 2.75 (1H, d, *J* = 11.8 Hz), 3.00 (1H, d, *J* = 12.0 Hz), 3.16–3.25 (2H, m), 4.93 (1H, quin, *J* = 7.3 Hz), 7.18–7.29 (5H, m), 8.77 (1H, d, *J* = 7.7 Hz). ^13^C NMR (125 MHz, DMSO-*d*_6_): δ = 22.1, 22.4, 24.8, 30.9, 42.1, 44.7, 46.7, 48.3, 68.7, 125.7, 126.5, 128.1, 145.5, 171.4. Anal. Calcd for C_18_H_28_N_2_O_2_: C, 71.02; H, 9.27; N, 9.20. Found: C, 71.00; H, 9.25; N, 9.23.

#### 4.8.4. Ethyl 3-((R)-3-amino-2-((1S,2R,4R)-2-hydroxy-4-methylcyclohexyl)propanamido)propanoate (**30**)

Prepared from **28** with 5% Pd/C for 24 h. Yield: 50%, colorless oil. ${\left[\alpha \right]}_{D}^{20}$ = −15.5(c 0.31, MeOH). ^1^H NMR (500 MHz, DMSO-*d*_6_): δ = 0.66–0.73 (1H, m), 0.79–1.05 (2H, m), 0.83 (3H, d, *J* = 6.2 Hz), 1.17 (3H, t, *J* = 7.0 Hz), 1.22–1.28 (1H, m), 1.29–1.35 (1H, m) 1.41–1.53 (3H, m), 1.73–1.82 (1H, m), 2.36–2.43 (3H, m), 2.59–2.76 (3H, m), 3.16 (1H, s), 3.19–3.24 (3H, m), 4.04 (2H, q, *J* = 7.1 Hz). ^13^C NMR (125 MHz, DMSO-*d*_6_): δ = 14.1, 22.2, 26.0, 31.1, 34.0, 34.4, 34.7, 44.8, 46.4, 59.9, 69.3. Anal. Calcd for C_15_H_28_N_2_O_2_: C, 59.97; H, 9.40; N, 9.33. Found: C, 60.00; H, 9.45; N, 9.30.

#### 4.8.5. (S)-Ethyl 2-((R)-3-amino-2-((1S,2R,4R)-2-hydroxy-4-methylcyclohexyl)propanamido)propanoate (**31**)

Prepared from **29** with 5% Pd/C for 24 h. Yield: 55%, colorless oil. ${\left[\alpha \right]}_{D}^{20}$ = −20.0 (c 0.30, MeOH). ^1^H NMR (500 MHz, DMSO-*d*_6_): δ = 0.70–0.77 (1H, m), 0.85 (3H, d, *J* = 6.4 Hz), 0.84–0.93 (2H, m), 1.15 (3H, t, *J* = 7.1 Hz), 1.28 (2H, d, *J* = 7.3 H), 1.36 (1H, br s), 1.47–1.56 (2H, m), 1.74 (1H, t, *J* = 11.6 Hz), 1.86 (1H, d, *J* = 12.7 Hz), 2.78 (1H, d, *J* = 11.1 Hz), 3.04 (1H, t, *J* = 11.8 Hz), 3.13 (1H, d, *J* = 10.3 Hz), 3.26 (1H, td, *J* = 3.6, 10.2 Hz), 4.04 (2H, q, *J* = 6.9 Hz), 4.26 (1H, quin, *J* = 7.0 Hz), 8.58 (1H, d, *J* = 1.3 Hz). ^13^C NMR (125 MHz, DMSO-*d*_6_): δ = 14.0, 16.5, 22.2, 24.8, 30.9, 34.2, 35.4, 42.3, 44.7, 46.5, 48.0, 60.4, 68.7, 172.3, 172.6. Anal. Calcd for C_15_H_28_N_2_O_2_: C, 59.97; H, 9.40; N, 9.33. Found: C, 59.97; H, 9.38; N, 9.35.

#### 4.8.6. (S)-3-Amino-N-benzyl-2-((1S,2S,4R)-2-hydroxy-4-methylcyclohexyl)propanamide (**35**)

Prepared from **32** with 5% Pd/C for 96 h. Yield: 70%, white crystals, m.p.: 270–275 °C. ${\left[\alpha \right]}_{D}^{20}$ = +36.0 (c 0.26, MeOH). ^1^H NMR (500 MHz, DMSO-*d*_6_): 0.74–0.86 (1H, m), 0.79 (3H, d, *J* = 6.4 Hz), 1.01 (1H, t, *J* = 12.8 Hz), 1.18–1.26 (2H, m), 1.34–1.40 (1H, m), 1.47 (1H, t, *J* = 11.4 Hz), 1.55 (1H, d, *J* = 12.5 Hz), 1.69 (2H, d, *J* = 11.0 Hz), 2.23–2.27 (1H, m), 2.67–2.71 (1H, m), 2.77–2.80 (1H, m), 3.84 (1H, s), 4.22–4.31 (1H, m), 7.20–7.32 (5H, m), 8.39 (1H, t, *J* = 5.6 Hz). ^13^C NMR (125 MHz, DMSO-*d*_6_): δ = 22.5, 25.1, 25.5, 34.6, 40.8, 41.3, 41.9, 42.3, 50.6, 64.3, 126.6, 127.2, 128.2, 139.9, 174.7. Anal. Calcd for C_17_H_26_N_2_O_2_: C, 70.31; H, 9.02; N, 9.65. Found: C, 70.29; H, 9.03; N, 9.60.

#### 4.8.7. (S)-3-Amino-2-((1S,2S,4R)-2-hydroxy-4-methylcyclohexyl)-*N*-((R)-1-phenylethyl)propanamide (**36**)

Prepared from **33** with 5% Pd/C for 240 h. Yield: 70%, white crystals, m.p.: 245–250 °C. ${\left[\alpha \right]}_{D}^{20}$ = +20.0 (c 0.24, MeOH). ^1^H NMR (500 MHz, DMSO-*d*_6_): δ = 0.65–0.72 (1H, m), 0.78 (3H, d, *J* = 6.3 Hz), 0.95–1.02 (1H, m), 1.36 (3H, d, *J* = 7.0 Hz), 1.35–1.39 (1H, m), 1.48–1.82 (2H, m), 1.65 (2H, d, *J* = 12.2 Hz), 2.58–2.64 (1H, m), 2.93 (1H, d, *J* = 12.1 Hz), 3.02–3.06 (1H, m), 3.74 (1H, s), 4.60 (1H, s), 4.93 (1H, quin, *J* = 7.3 Hz), 7.20–7.30 (5H, m), 7.87 (3H, br s), 8.68 (1H, d, *J* = 7.8 Hz). ^13^C NMR (125 MHz, DMSO-*d*_6_): δ = 22.3, 22.5, 24.4, 25.2, 34.3, 37.9, 42.0, 44.9, 48.3, 63.9, 126.0, 126.6, 128.1, 144.7, 171.7. Anal. Calcd for C_18_H_28_N_2_O_2_: C, 71.02; H, 9.27; N, 9.20. Found: C, 71.00; H, 9.25; N, 9.25.

#### 4.8.8. (S)-3-Amino-2-((1S,2S,4R)-2-hydroxy-4-methylcyclohexyl)-*N*-((S)-1-phenylethyl)propanamide (**37**)

Prepared from **34** with Pd(OH)_2_/C for 300 h. Yield: 52%, white crystals, m.p.: 225–228 °C. ${\left[\alpha \right]}_{D}^{20}$ = −32.0 (c 0.24, MeOH). ^1^H NMR (500 MHz, DMSO-*d*_6_): δ = 0.78–0.85 (1H, m), 0.82 (3H, d, *J* =*.*6.3 Hz), 0.96–1.19 (1H, m), 1.02 (3H, d, *J* = 6.3 Hz), 1.10 (3H, d, *J* = 6.0 Hz), 1.35 (3H, d, *J* = 7.0 Hz), 1.44–1.55 (2H, m), 1.61–1.73 (3H, m), 2.64 (1H, br s), 2.92 (1H, d, *J* = 11.4 Hz), 3.03–3.15 (2H, m), 3.89 (1H, s), 4.96 (1H, quin, *J* =.7.0 Hz), 7.20–7.36 (5H, m), 8.68 (1H, d, J = 7.5 Hz). ^13^C NMR (125 MHz, DMSO-*d*_6_): δ = 22.3, 22.5, 24.4, 25.3, 34.4, 41.3, 42.1, 48.0, 49.0, 64.1, 126.2, 126.7, 128.2, 144.2, 171.9. Anal. Calcd for C_18_H_28_N_2_O_2_: C, 71.02; H, 9.27; N, 9.20. Found: C, 70.97; H, 9.30; N, 9.17.

### 4.9. Determination of Antiproliferative Properties

The human cancer cell lines isolated from cervical adenocarcinoma (HeLa) and breast cancers (MCF7 and MDA-MB-231) were purchased from European Collection of Cell Cultures (Salisbury, UK). The cells were maintained in Minimum Essential Medium (MEM) supplemented with fetal calf serum (10%), non-essential amino acids (1%), and penicillin-streptomycin (1%) at 37 °C in a humidified atmosphere containing 5% CO_2_. All media and supplements for these experiments were obtained from Lonza Group Ltd. (Basel, Switzerland). The antiproliferative properties of the prepared compounds were determined by the MTT [3-(4,5-dimethylthiazol-2-yl)-2,5-diphenyltetrazolium bromide] assay [33]. Briefly, cells were seeded into 96 well plates (5000 cells/well) and incubated with the tested compounds at 10 and 30 µM under cell-culturing conditions for 72 h. Then MTT solution (5 mg/mL) was added to each sample, which were incubated for a further 4 h. The formazan crystals precipitated were dissolved in 100 µL dimethyl sulfoxide, and the absorbance was measured at 545 nm with a microplate reader (Awareness Technology, Palm City, FL, USA). Two independent experiments were performed with five wells for each condition. Cisplatin (Ebewe GmbH, Unterach, Austria), a clinically used anticancer agent, was used as a reference agent. Calculations were performed by means of the GraphPad Prism 5.01 software (GraphPad Software Inc., San Diego, CA, USA).

## Figures and Tables

**Table 1:** Nucleophilic addition reaction of **2** with benzylamine.

Entry	Solvent	Temperature (°C)	Reaction Time (h)	Products Ratios (%)		Yield of 5a (%)
				5a	5b	
1	DCM	0	20	44	56	10
2	-	0	20	50	50	30
3	DMF	0	20	67	33	44
4	MeOH	0	20	92	8	30
5	*i*-PrOH	0	20	94	6	55
6	EtOH	0	20	94	6	60
7	EtOH	25	20	94	6	65
8	EtOH	40	15	80	20	55
9	EtOH	50	10	80	20	55
10	EtOH	60	7	80	20	50
11	EtOH	70	5	80	20	45

**Table 2:** Nucleophilic addition reaction of amines with **2**.

Entry	Compound	R^1^	R^2^	Yield (%)
1	6	H	(*R*)-*α*-Methylbenzyl	75
2	7	H	(*S*)-*α*-Methylbenzyl	71
3	8	C_2_H_5_	C_2_H_5_	50
4	9	-(CH_2_)_5_-		47
5	10	Benzyl	Benzyl	59
6	11	H	*β*-Alanine ethyl ester	60
7	12	H	L-Alanine ethyl ester	40

**Table 3:** Nucleophilic addition of amines with **4**.

Entry	Compound	R^1^	R^2^	Temperature (°C)	Yield (%)
1	13	H	Benzyl	25	60
2	14	H	(*R*)-*α*-Methylbenzyl	25	65
3	15	H	(*S*)-*α*-Methylbenzyl	25	70
4	16	C_2_H_5_	C_2_H_5_	25	50
5	17	-(CH_2_)_5_-		25	53
6	18	Benzyl	Benzyl	25	50
7	19	H	*β*-Alanine ethyl ester	70	60
8	20	H	L-Alanine ethyl ester	70	44

**Table 4:** Preparation of *β*-aminoamides from **2** and **13**–**15**.

Entry	Compound	R	Temperature (°C)	Reaction Time (h)	Yield (%)
1	21	Benzyl	25	20	90
2	22	(*R*)-*α*-Methylbenzyl	70	48	58
3	23	(*S*)-*α*-Methylbenzyl	70	48	54
4	32	Benzyl	70	24	70
5	33	(*R*)-*α*-Methylbenzyl	70	72	42
6	34	(*S*)-*α*-Methylbenzyl	70	72	45

**Table 5:** Debenzylation of aminoamides **21**–**23** and **32**–**34**.

Entry	Compound	R	Catalyst	Reaction Time (h)	Yield (%)
1	24	Benzyl	5% Pd/C	96	80
2	25	(*R*)-*α*-Methylbenzyl	5% Pd/C	168	62
3	26	(*S*)-*α*-Methylbenzyl	Pd(OH)_2_/C	200	65
4	35	Benzyl	5% Pd/C	96	70
5	36	(*R*)-*α*-Methylbenzyl	5% Pd/C	240	70
6	37	(*S*)-*α*-Methylbenzyl	Pd(OH)_2_/C	300	52

**Table 6:** Antiproliferative activities of the tested monoterpene analogs.

Analog	Conc. (µM)	Growth Inhibition (%) ± SEM *		
		HeLa	MCF7	MDA-MB-231
2	10	–	–	–
	30	–	18.33 ± 2.90	–
4	10	–	–	–
	30	21.10 ± 2.44	–	–
5	10	16.90 ± 2.60	18.76 ± 2.50	–
	30	28.74 ± 2.30	31.25 ± 3.01	–
7	10	–	–	–
	30	–	17.29 ± 2.90	–
21	10			
	30	31.79 ± 1.95	24.36 ± 2.42	22.75 ± 1.84
22	10	–	–	–
	30	27.09 ± 1.66	–	–
23	10	41.25 ± 2.60	33.96 ± 1.84	24.71 ± 1.86
	30	94.83 ± 0.73	87.93 ± 1.47	70.56 ± 3.51
29	10	–	17.00 ± 2.51	–
	30	–	36.45 ± 1.00	23.43 ± 2.03
32	10	24.36 ± 2.70	17.06 ± 1.46	–
	30	32.43 ± 0.52	40.40 ± 2.88	–
34	10	–	22.70 ± 1.82	19.51 ± 2.35
	30	36.04 ± 0.51	45.41 ± 2.92	34.61 ± 2.22
cisplatin	10	42.61 ± 2.33	53.03 ± 2.29	67.51 ± 1.01
	30	99.93 ± 0.26	86.90 ± 1.24	87.75 ± 1.10

* Growth inhibition values less than 15% are considered negligible and are not given numerically. Compounds **6**, **8**, **9**, **13**, **26**, **27,** and **28** were also examined but did not elicit 15% growth inhibition even at 30 μM.

## Supplementary Materials

Supplementary materials can be found at http://www.mdpi.com/1422-0067/19/11/3522/s1.

## Abbreviations

DCM	Dichloromethane
DMF	Dimethylformamide
THF	Tetrahydrofuran
EtOH	Ethanol
