# Supplementary material for: Synthesis and Transformation of (-)-Isopulegol-Based Chiral β-Aminolactones and β-Aminoamides
Source: Int J Mol Sci. 2018 Nov 8;19(11):3522. doi: 10.3390/ijms19113522 (PMC6274863; doi:10.3390/ijms19113522)

Supporting informations
for
Synthesis and transformation of (-)-isopulegol-based chiral *β*-aminolactones and *β*-aminoamides

Tam Minh Le[1,2], Péter Bérdi[3], Zupkó István[3,4], Ferenc Fülöp[1,2], Zsolt Szakonyi[1,4,*]

[1] Institute of Pharmaceutical Chemistry, University of Szeged, H-6720 Szeged, Eötvös utca 6, Hungary; leminhtam@pharm.u-szeged.hu; fulop@pharm.u-szeged.hu
[2] Stereochemistry Research Group of the Hungarian Academy of Sciences, H-6720 Szeged, Eötvös utca 6, Hungary
[3] Department of Pharmacodynamics and Biopharmacy, University of Szeged, H-6720 Szeged, Eötvös utca 6, Hungary; zupko@pharm.u-szeged.hu
[4] Interdisciplinary Centre of Natural Products, University of Szeged, H-6720 Szeged, Eötvös utca 6, Hungary
[*] Correspondence: szakonyi@pharm.u-szeged.hu; Tel.: +36-62-546809; Fax: +36-62-545705

Contents

^{1}H, ^{13}C NMR spectra of new compounds 3 – 70

^{1}H-NMR of compound **5a** + **5b**

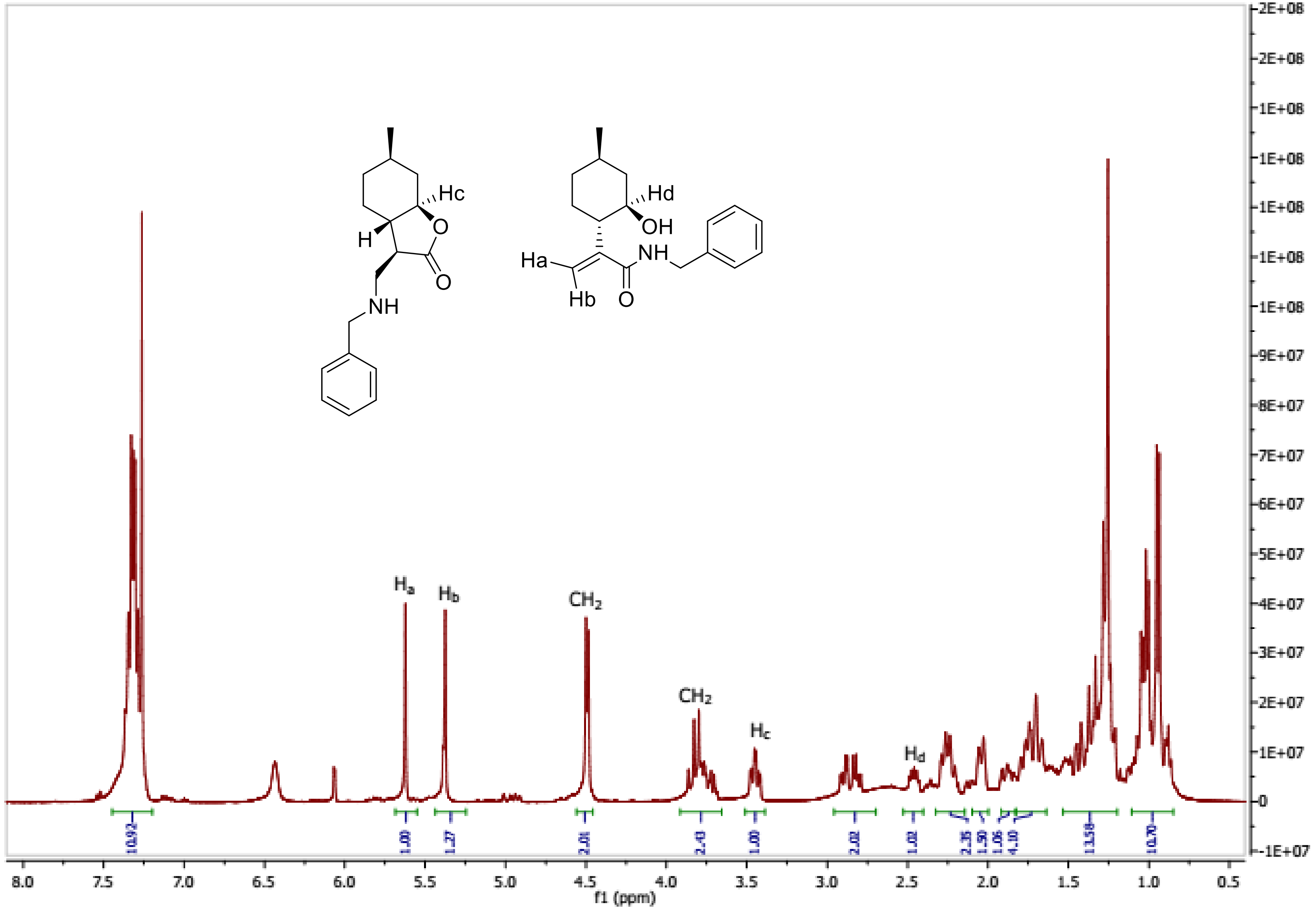

^{1}H-NMR of compound **5a**

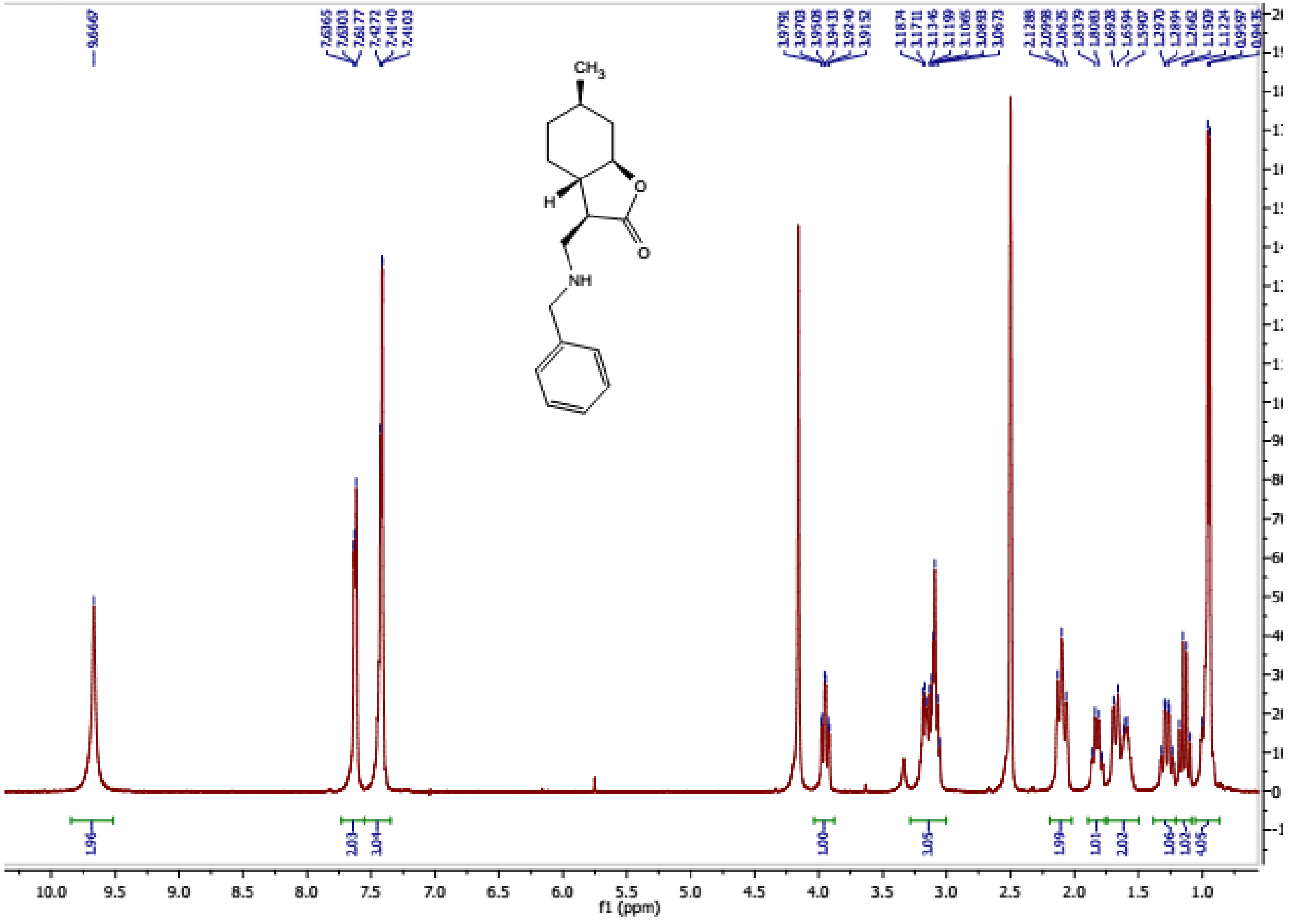

^{13}C-NMR of compound **5a**

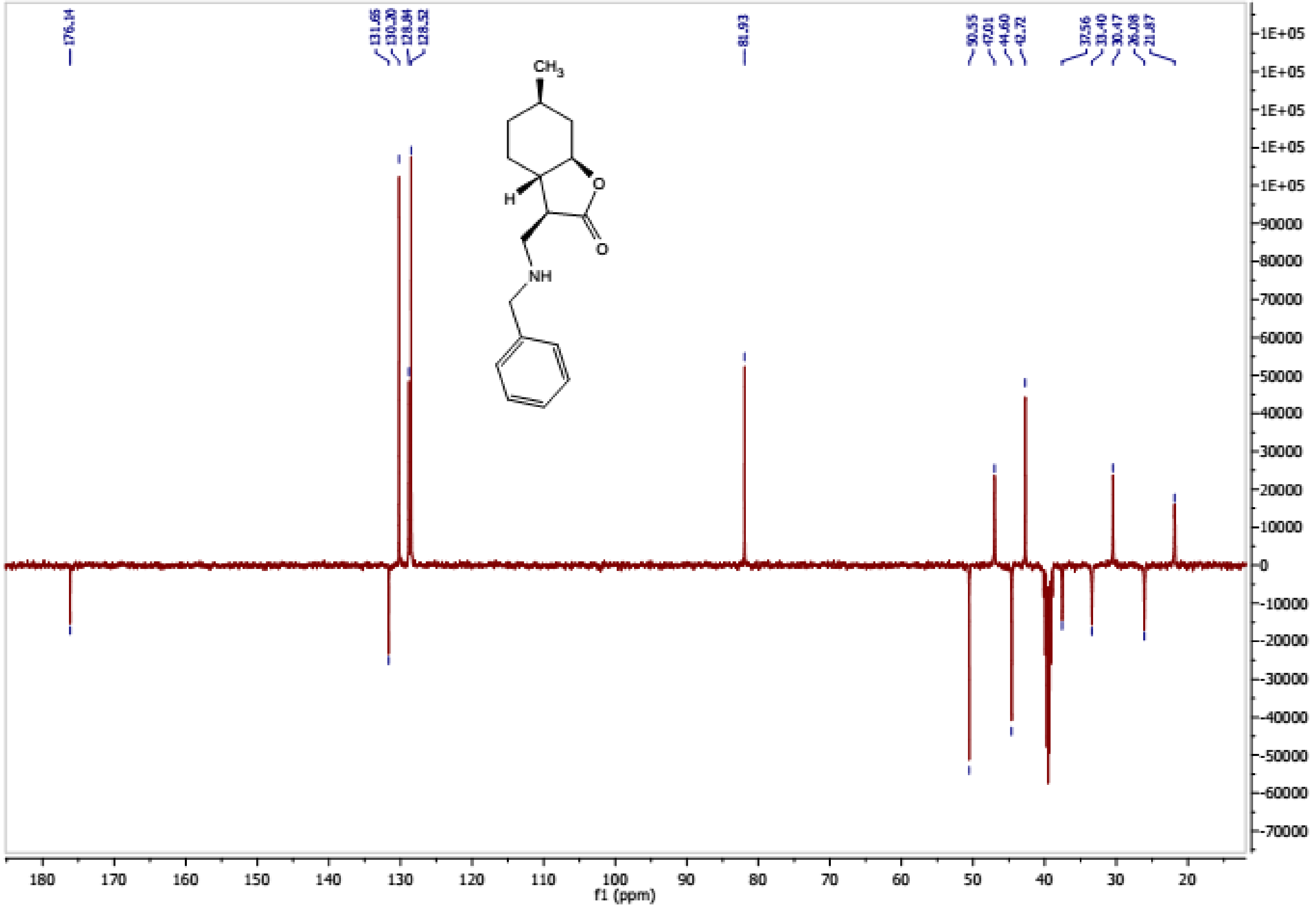

^{1}H-NMR of compound **6**

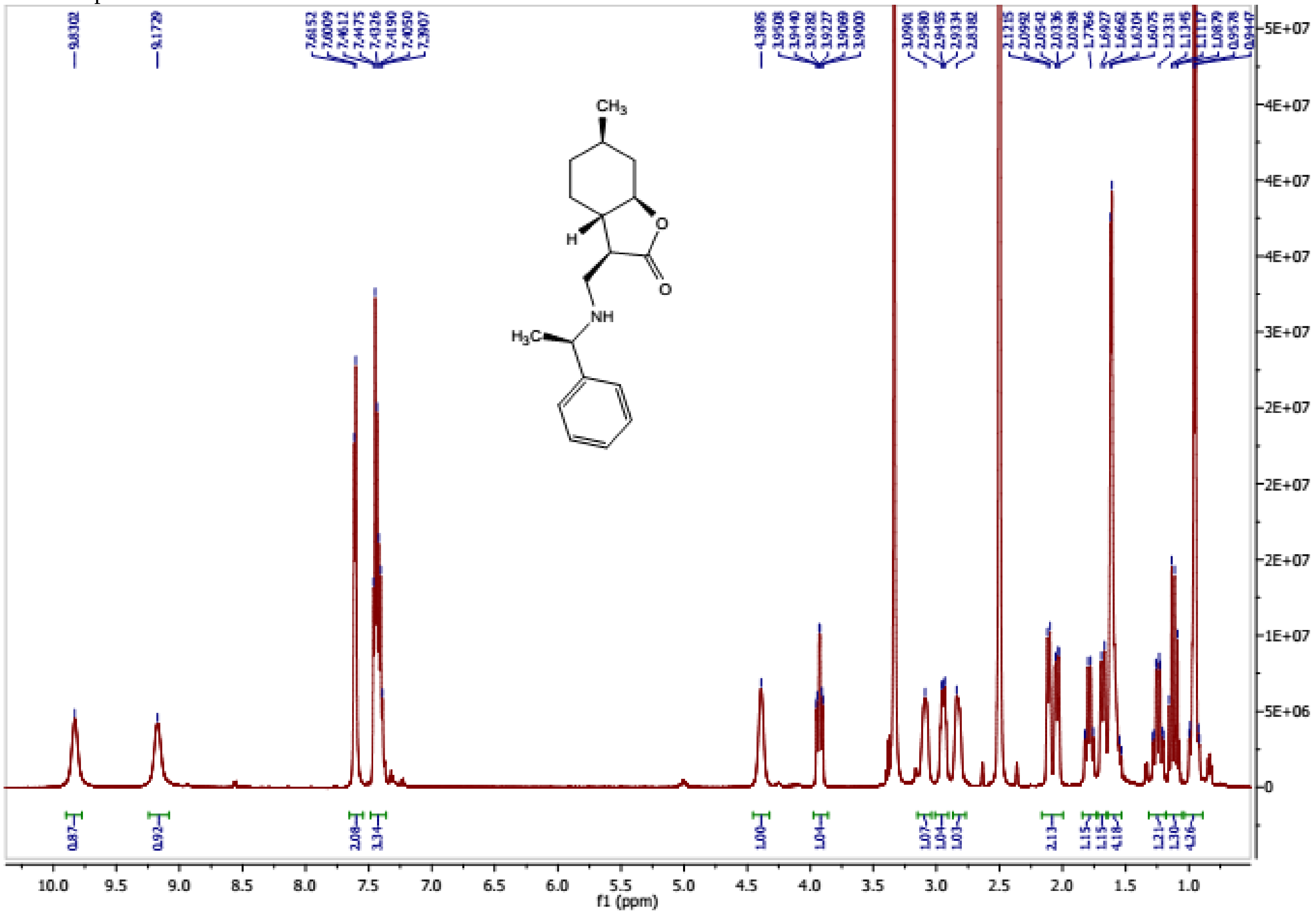

^{13}C-NMR of compound **6**

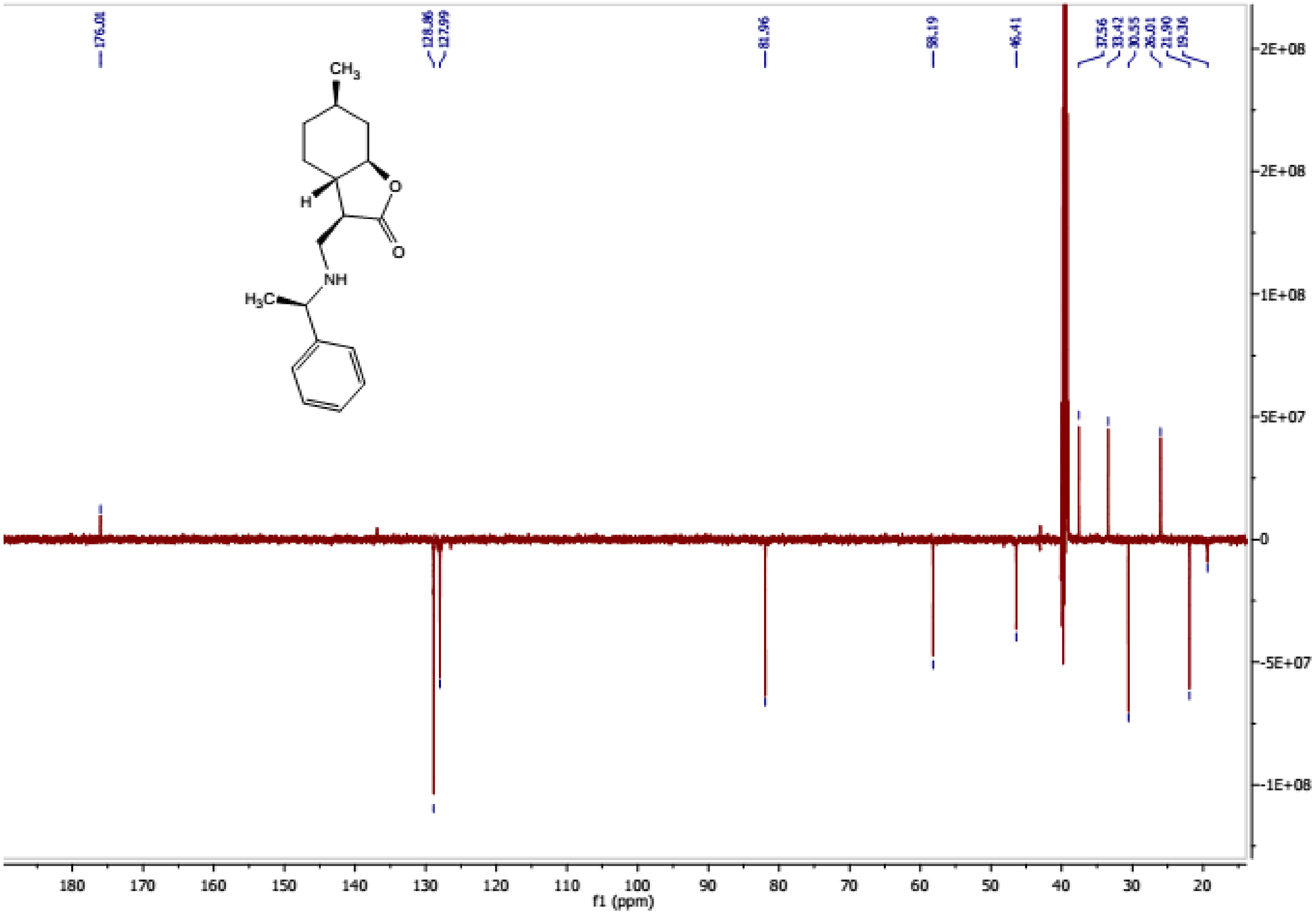

^{1}H-NMR of compound **7**

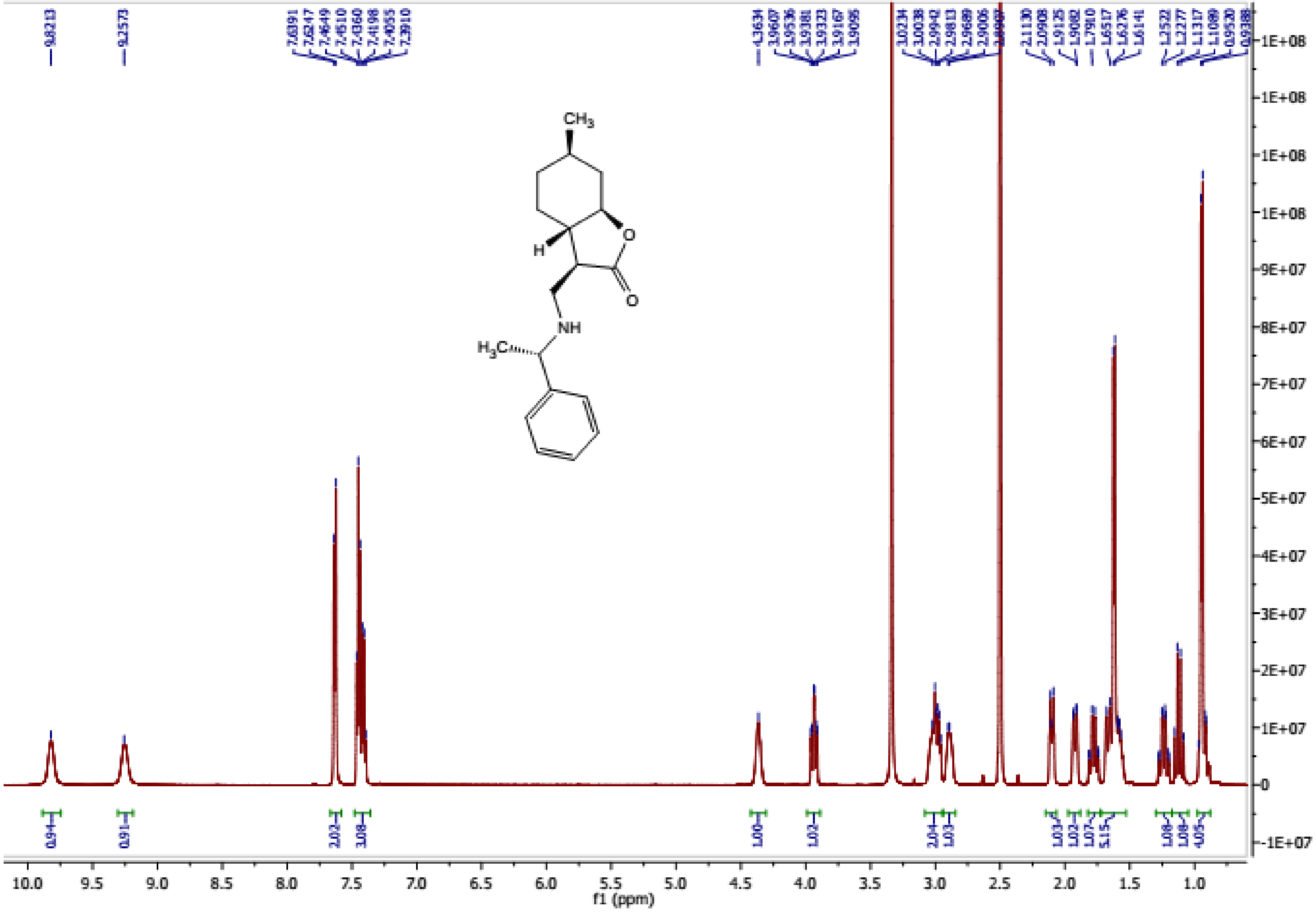

^{13}C-NMR of compound **7**

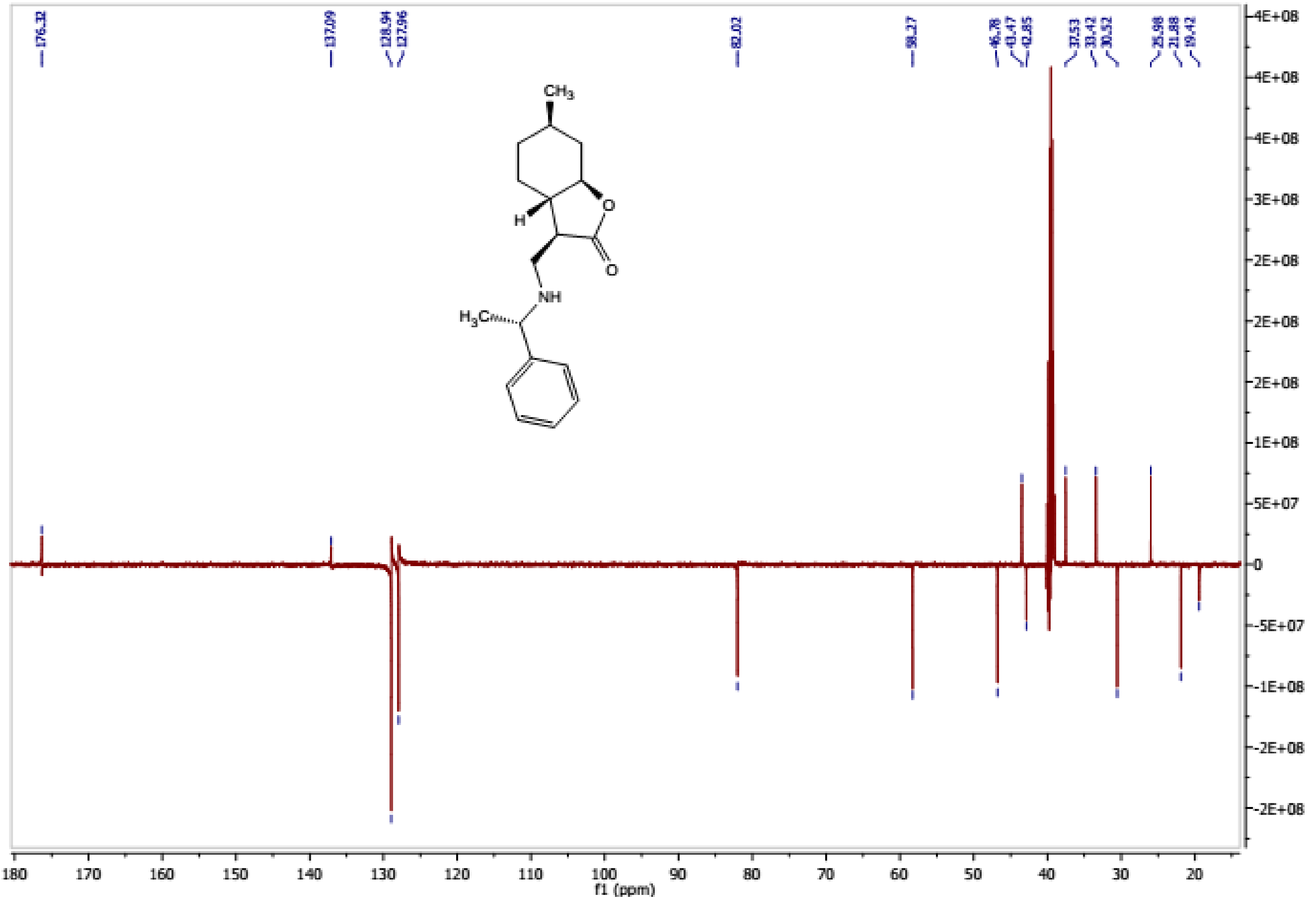

^{1}H-NMR of compound **8**

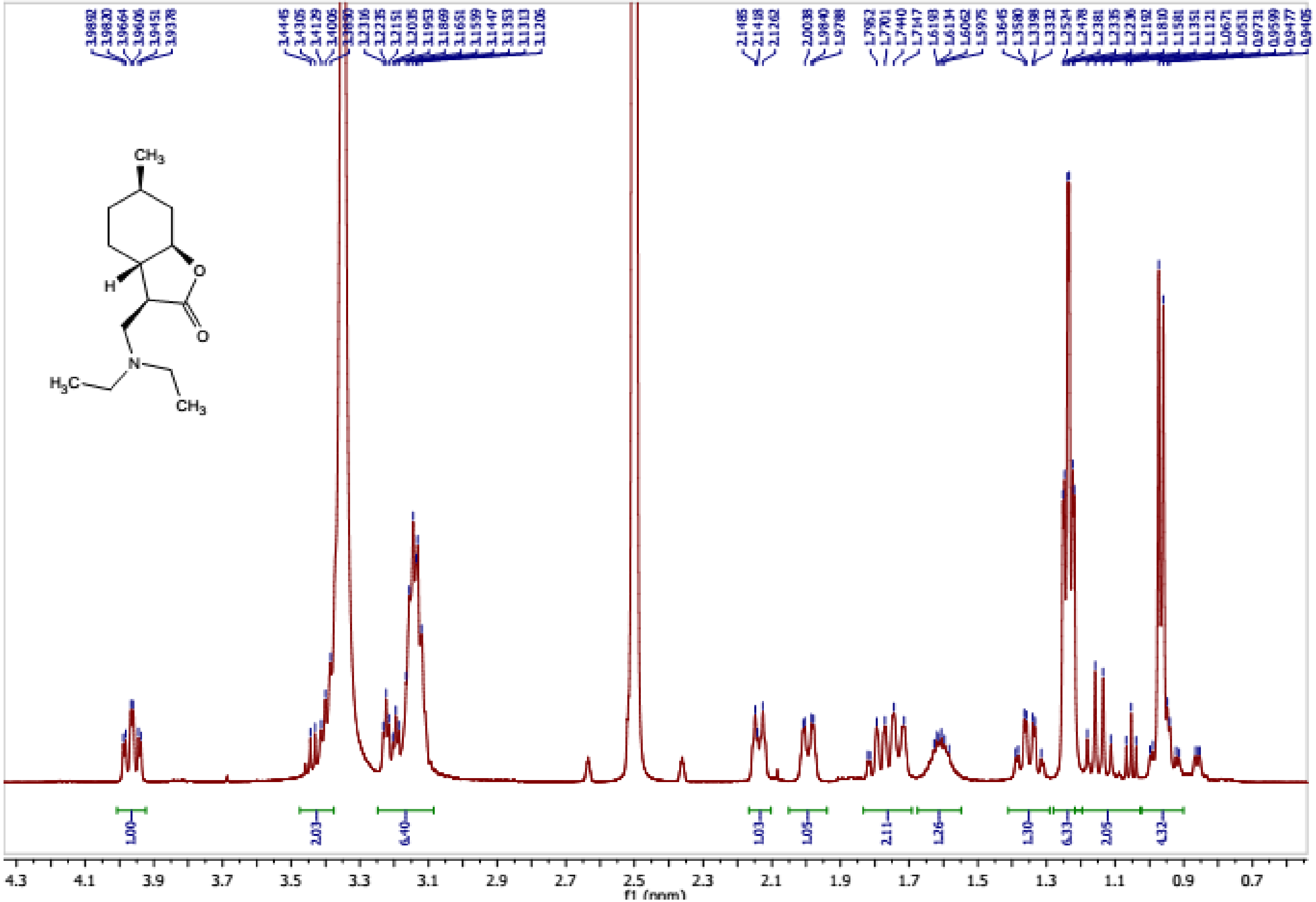

^{13}C-NMR of compound **8**

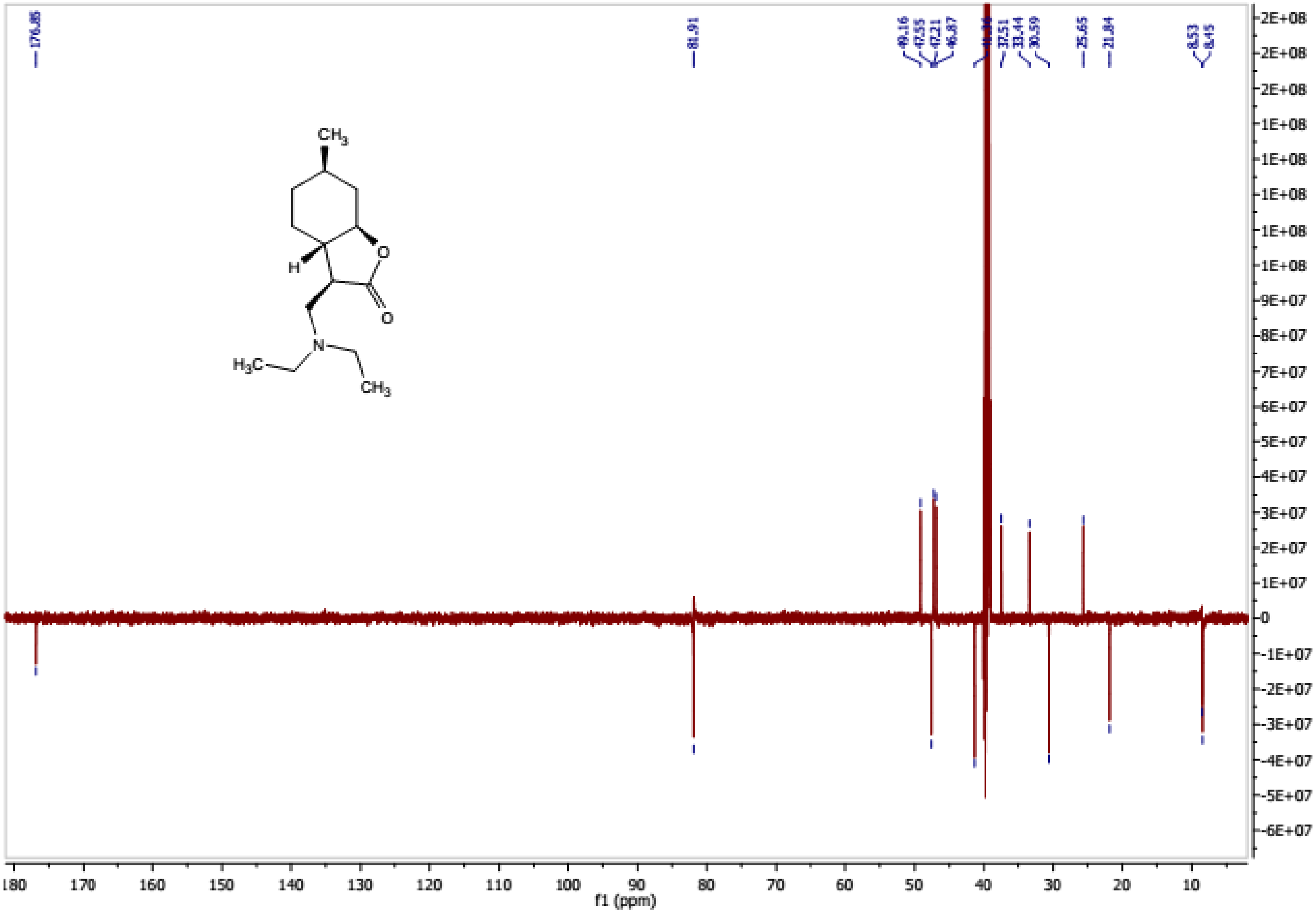

^{1}H-NMR of compound **9**

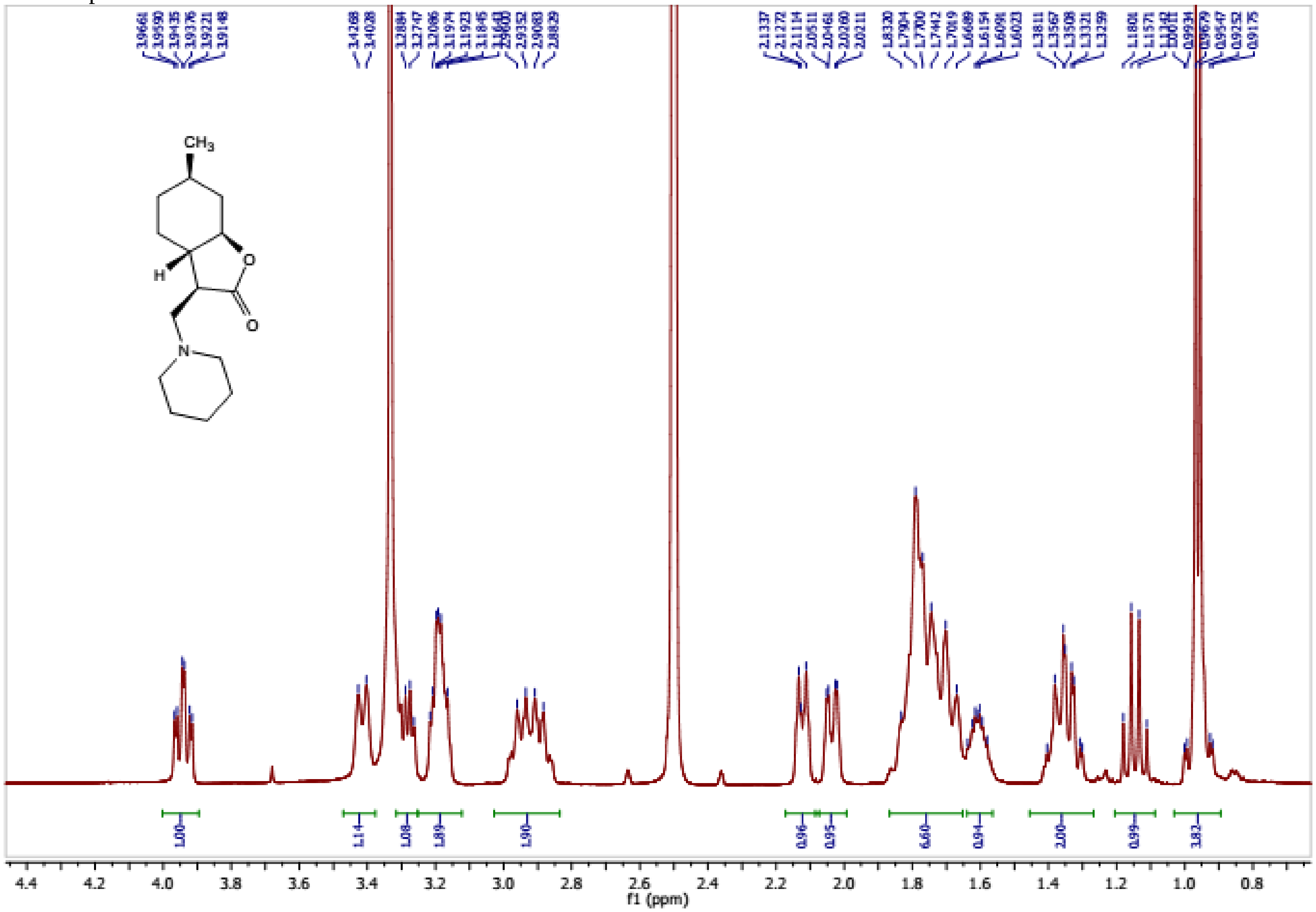

^{13}C-NMR of compound **9**

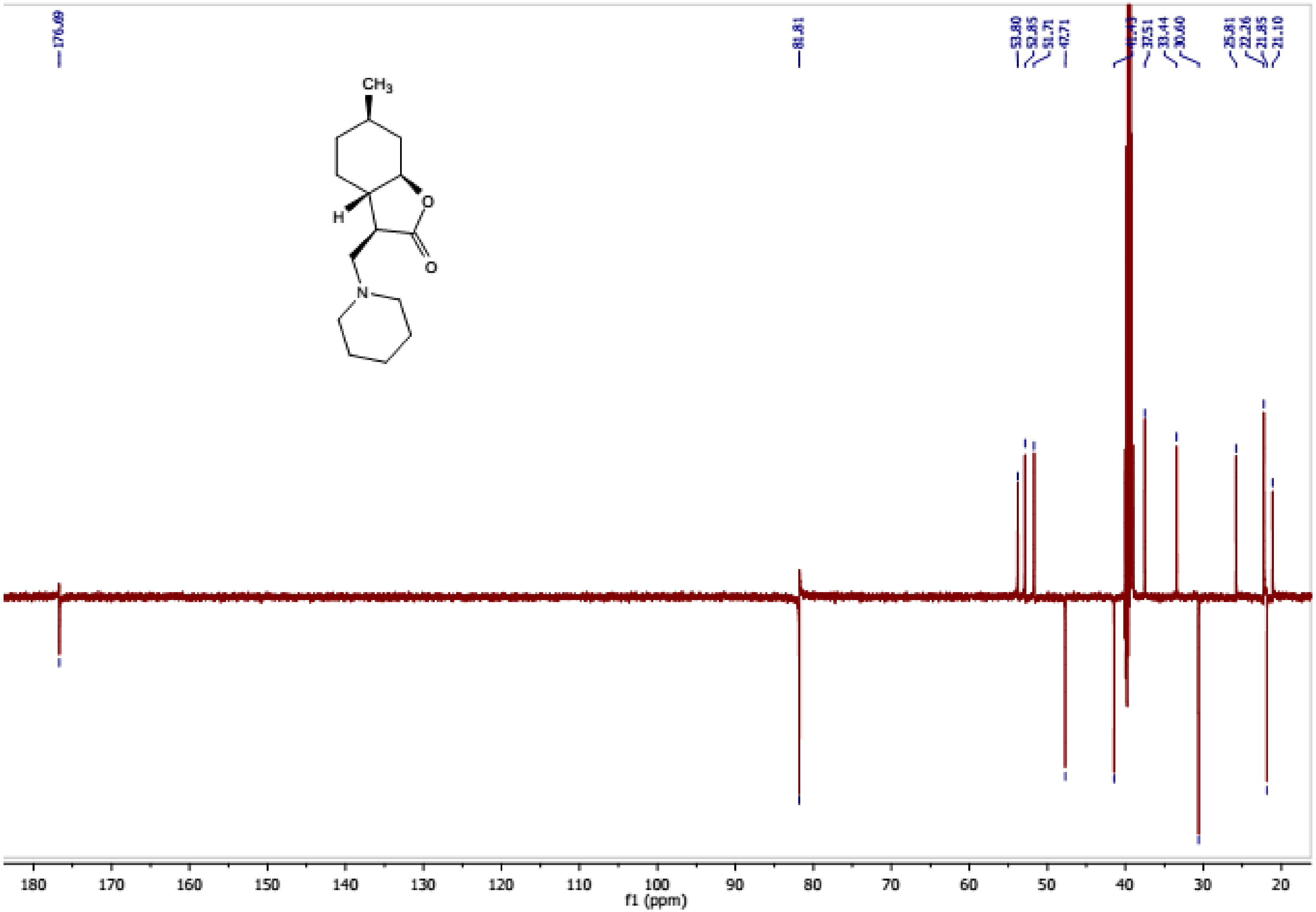

^{1}H-NMR of compound **10**

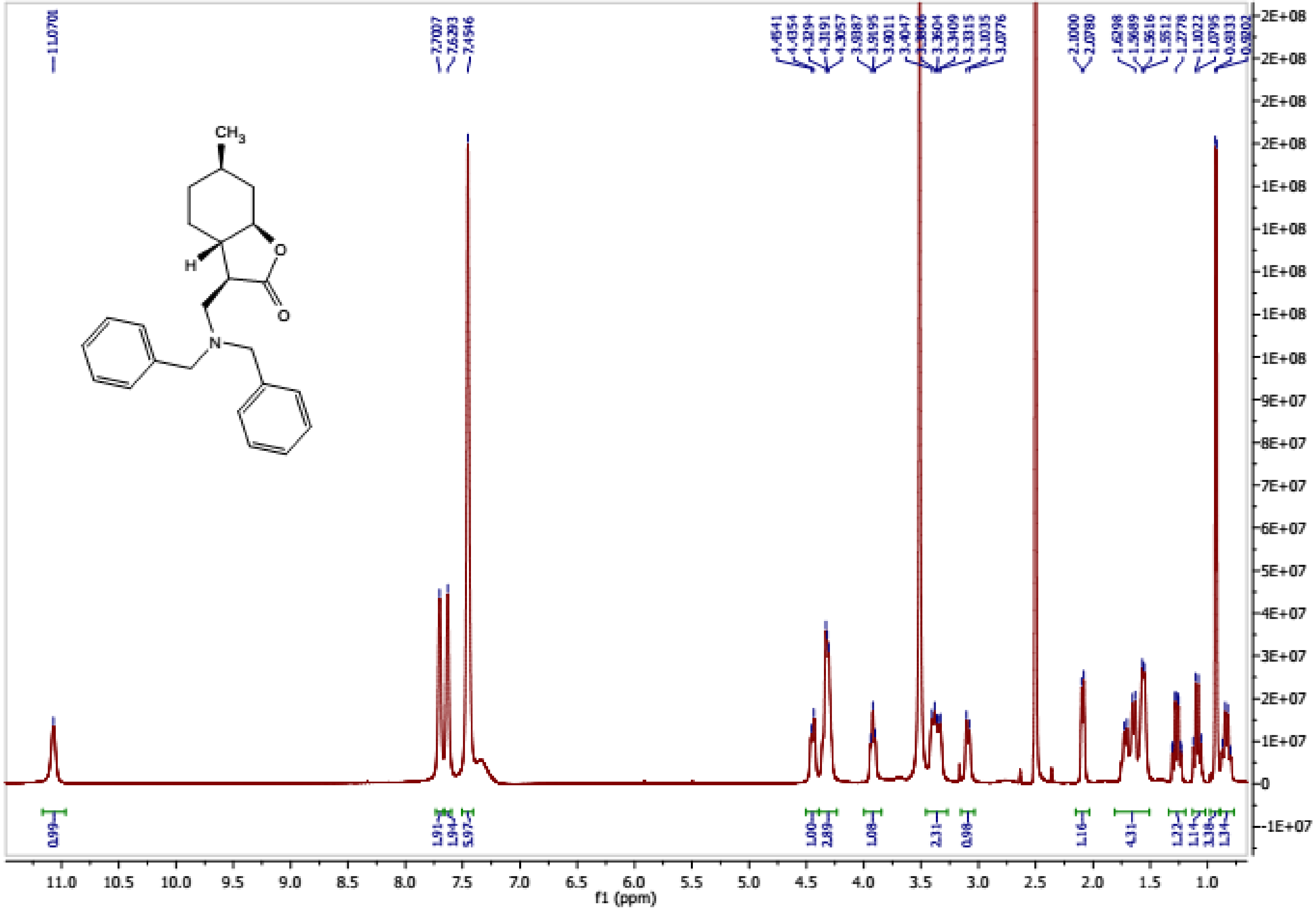

^{13}C-NMR of compound **10**

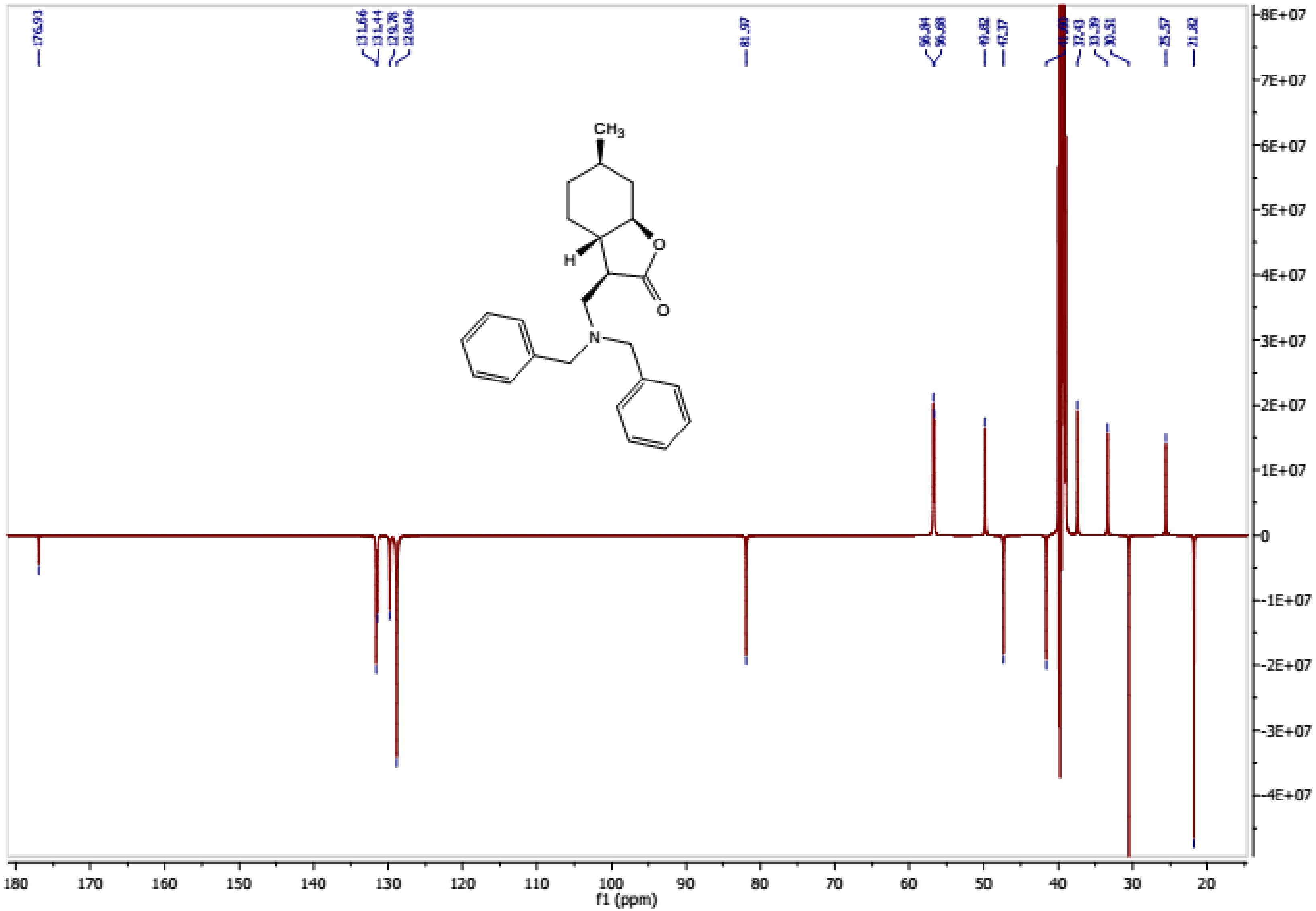

^{1}H-NMR of compound **11**

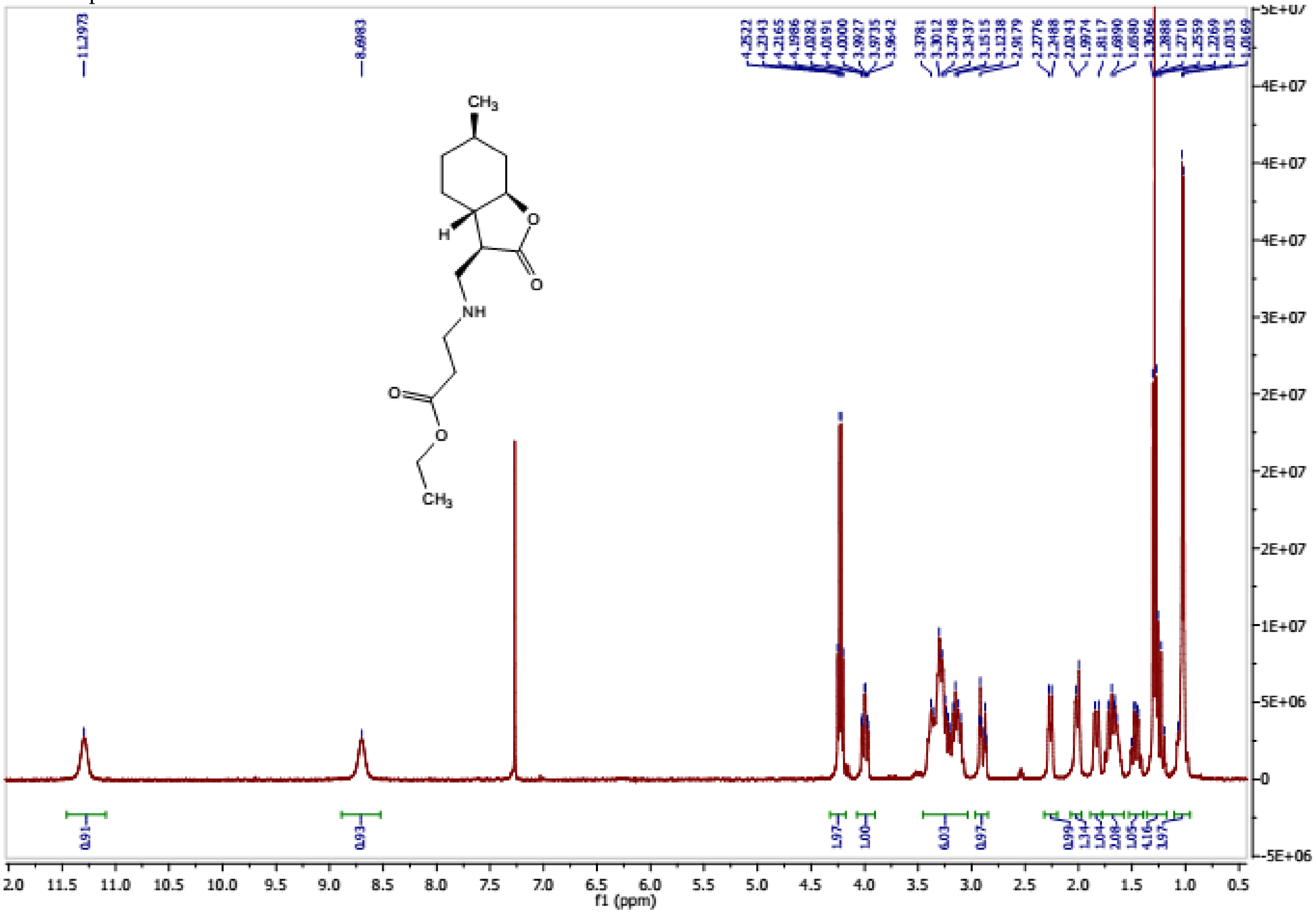

^{13}C-NMR of compound **11**

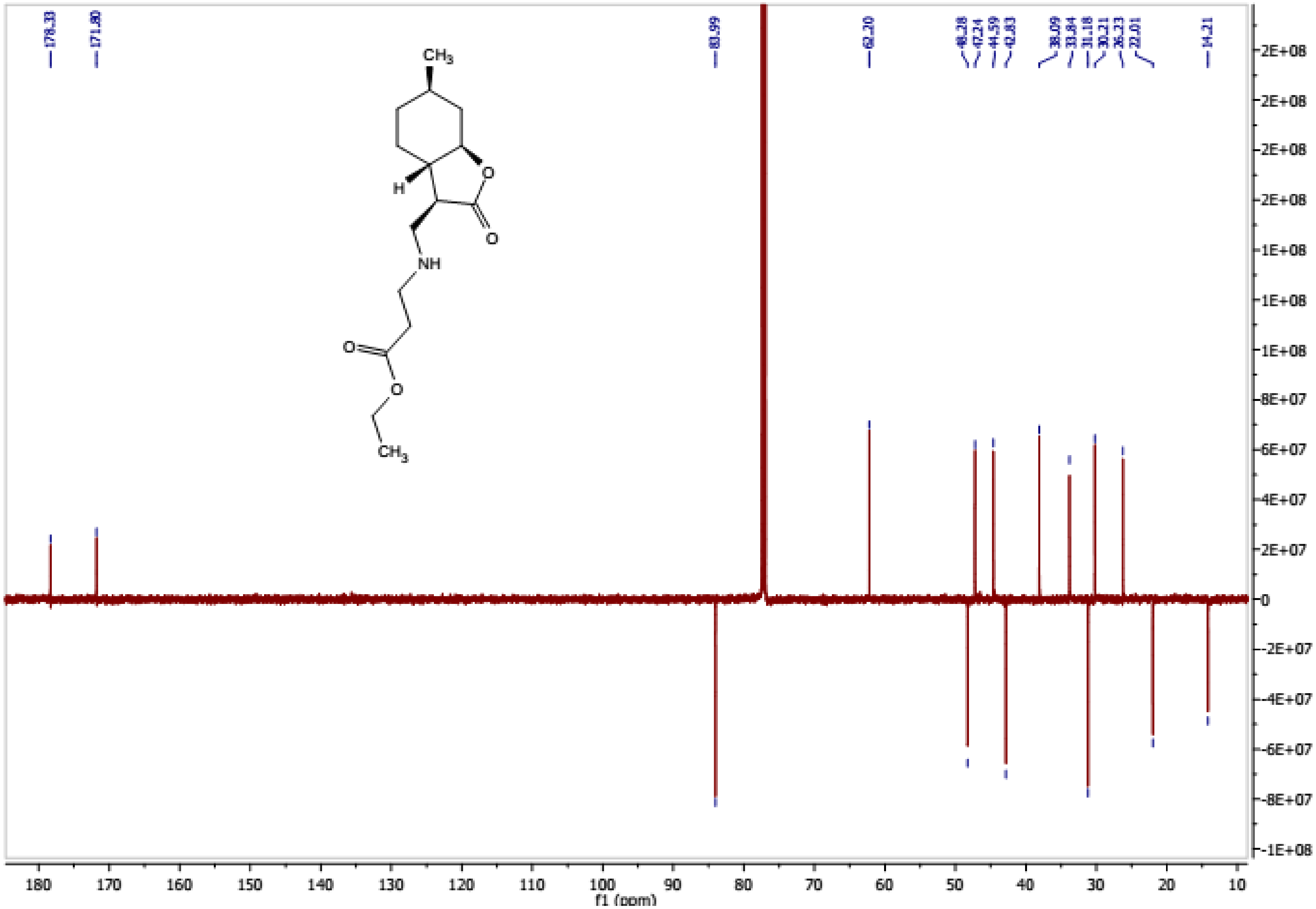

^{1}H-NMR of compound **12**

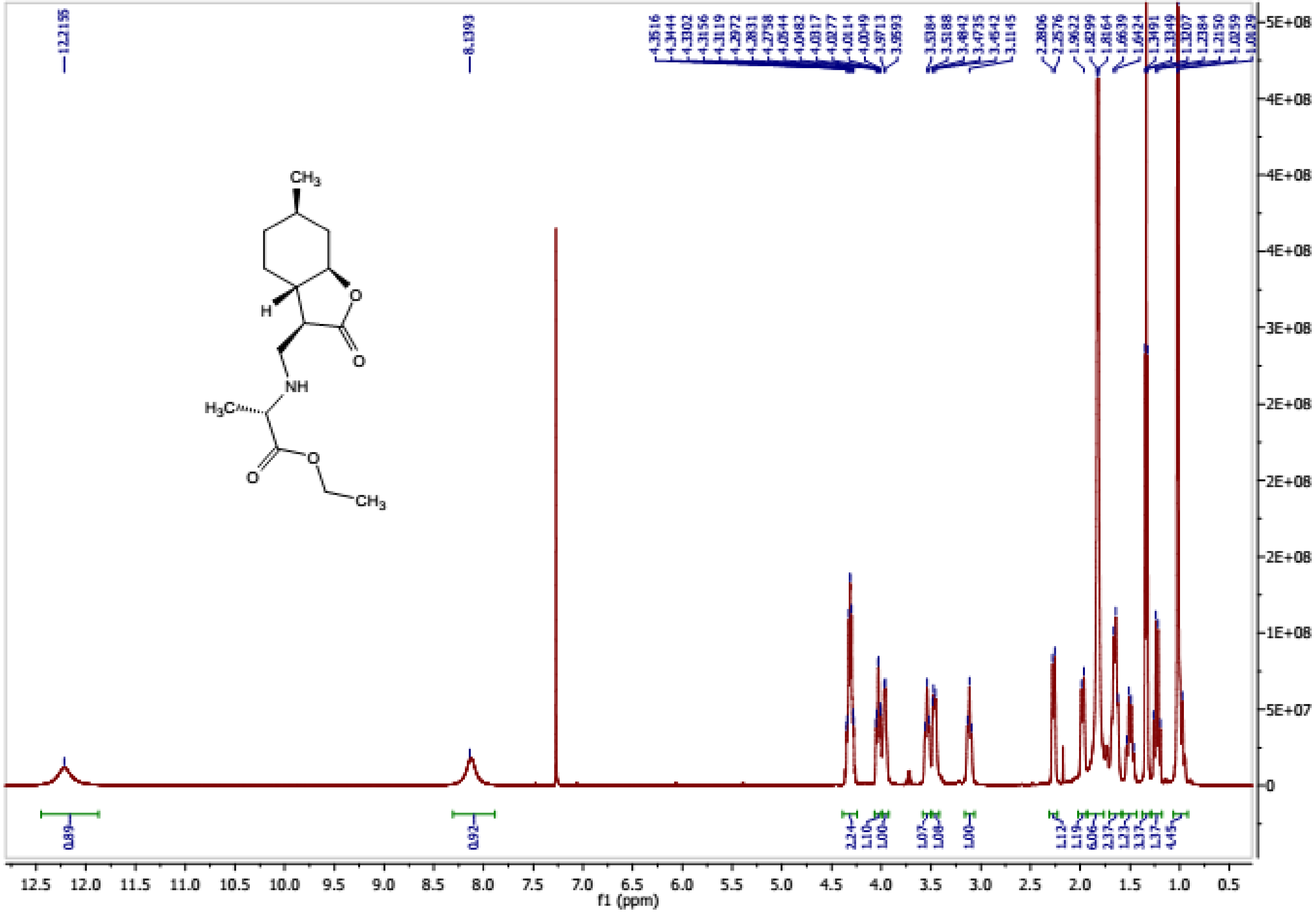

^{13}C-NMR of compound **12**

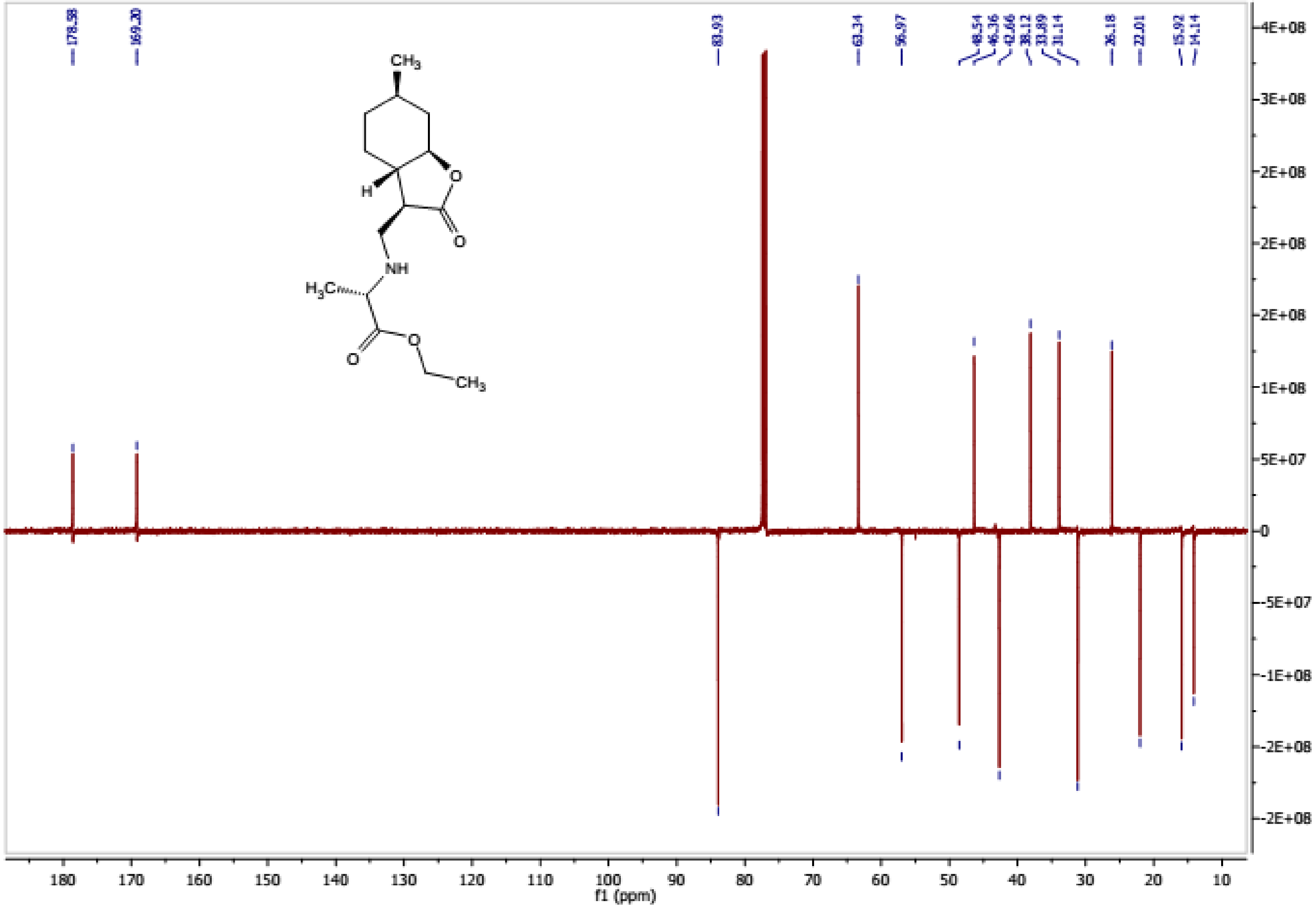

^{1}H-NMR of compound **13**

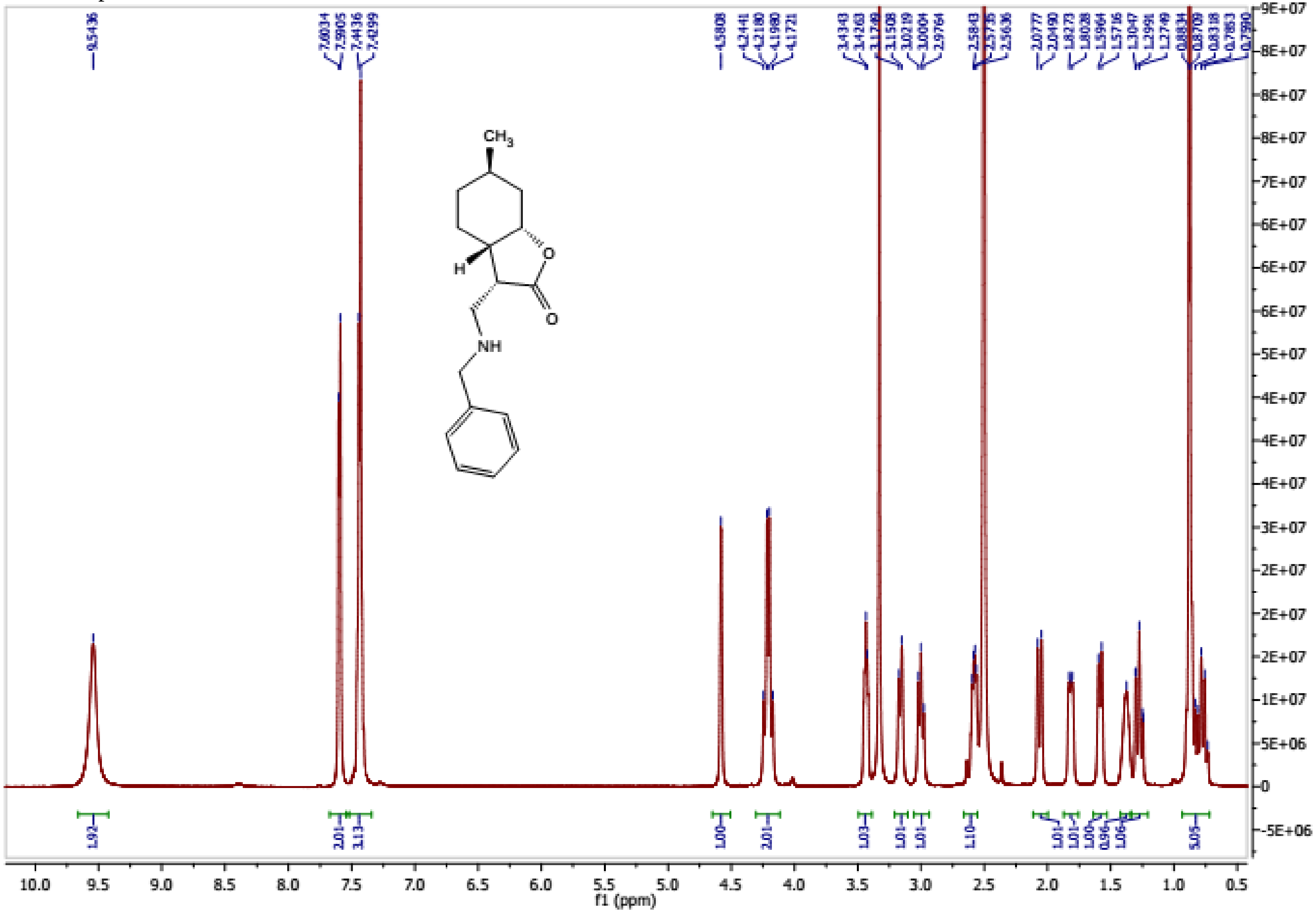

^{13}C-NMR of compound **13**

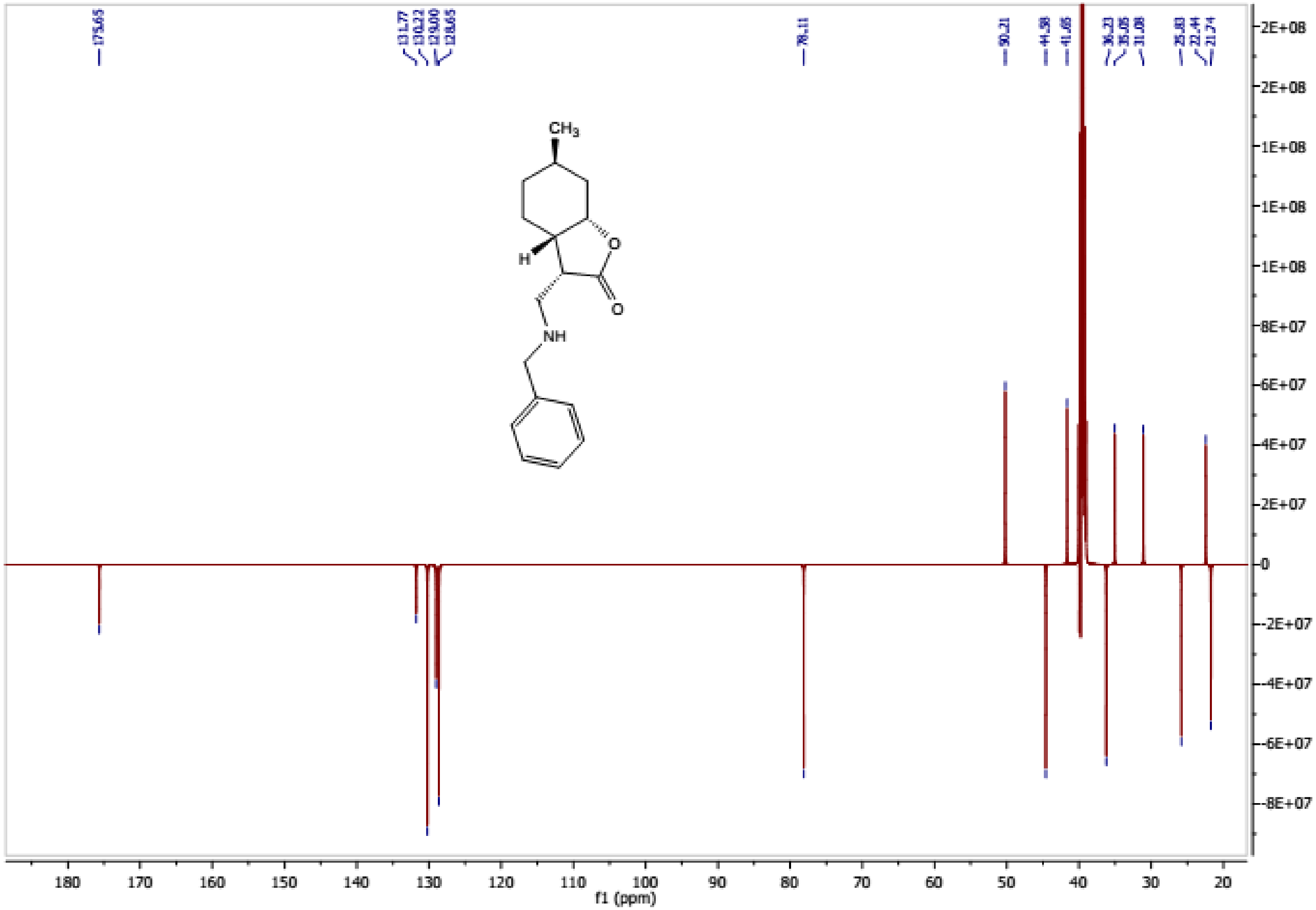

^{1}H-NMR of compound **14**

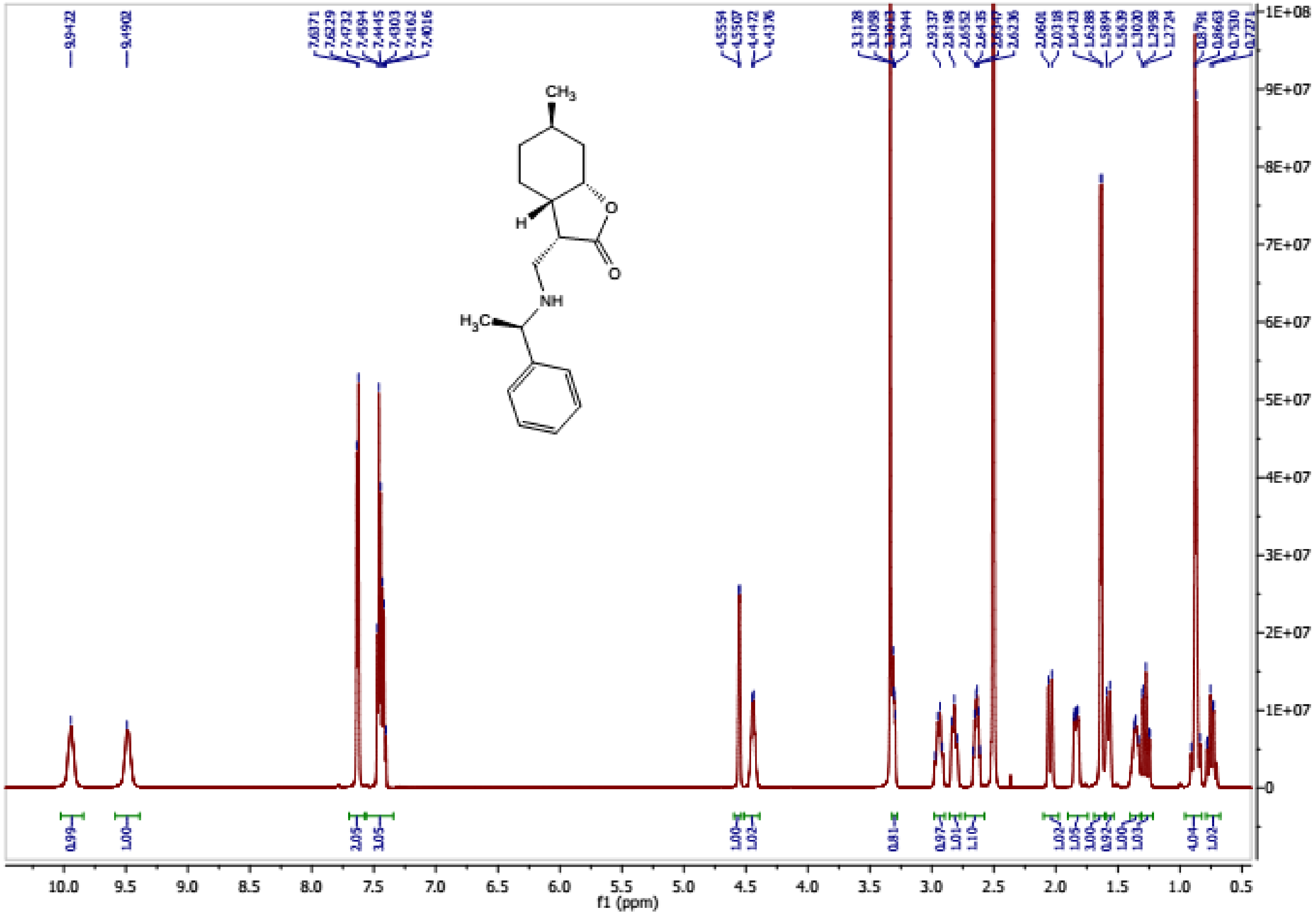

^{13}C-NMR of compound **14**

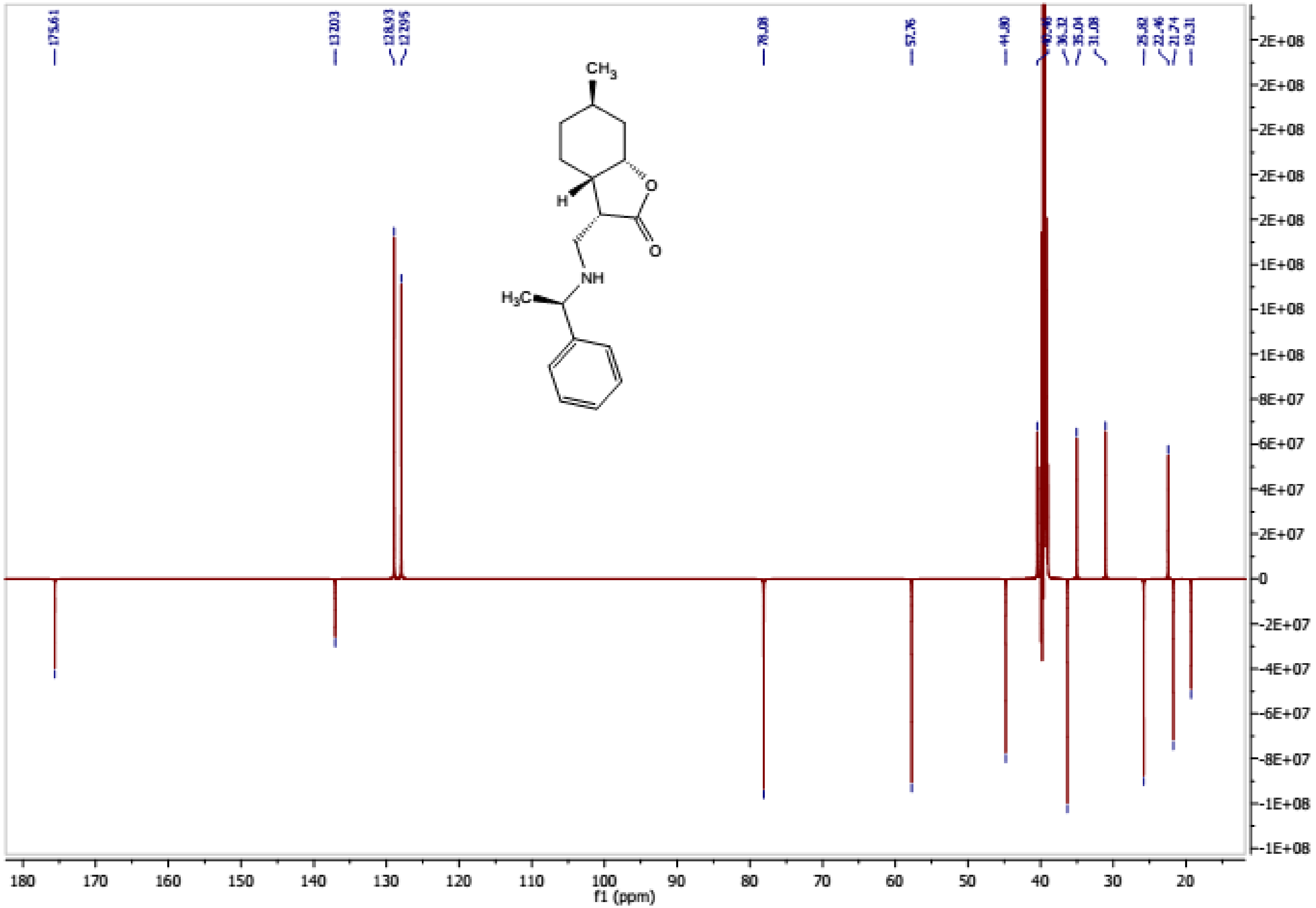

^{1}H-NMR of compound **15**

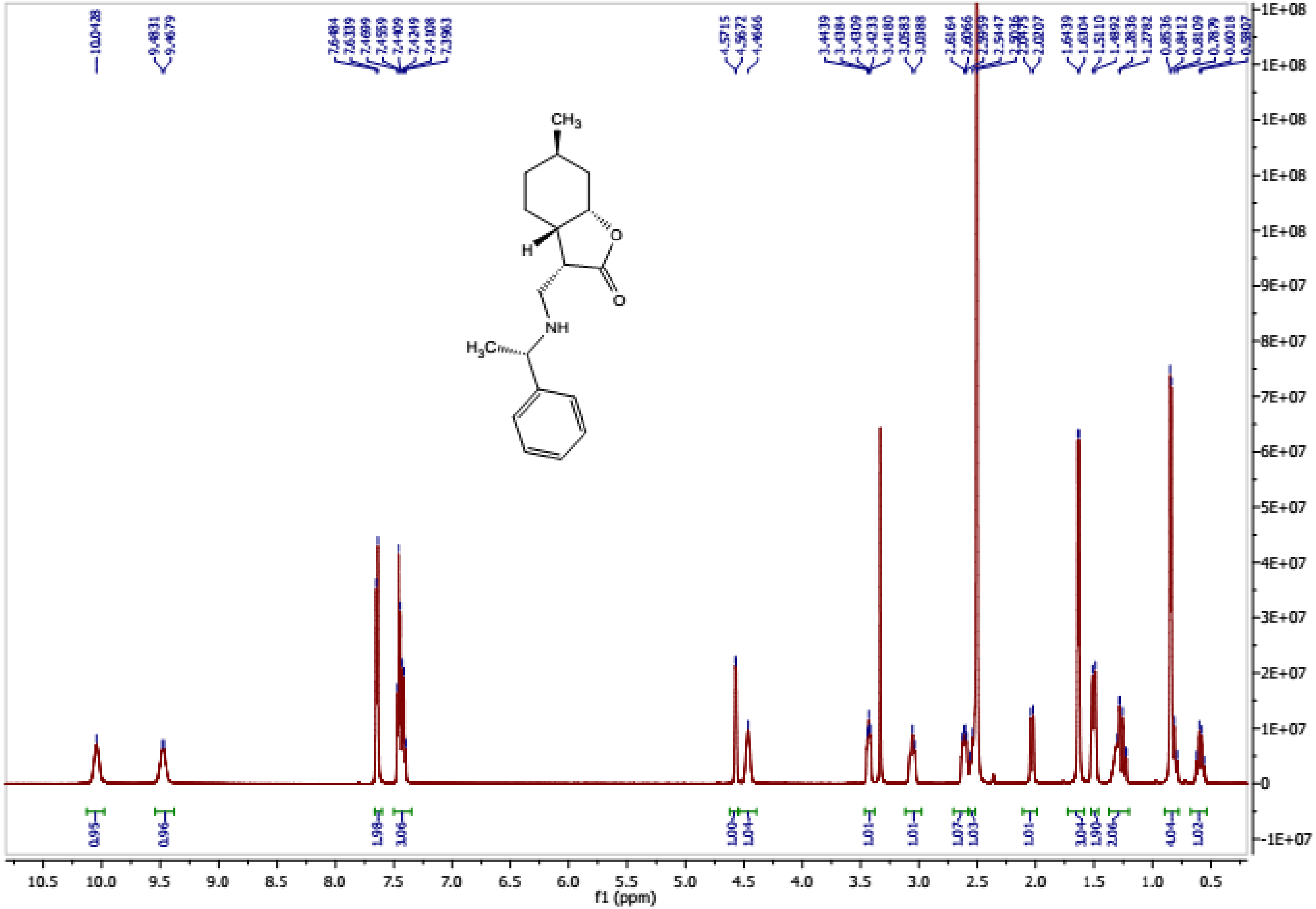

^{13}C-NMR of compound **15**

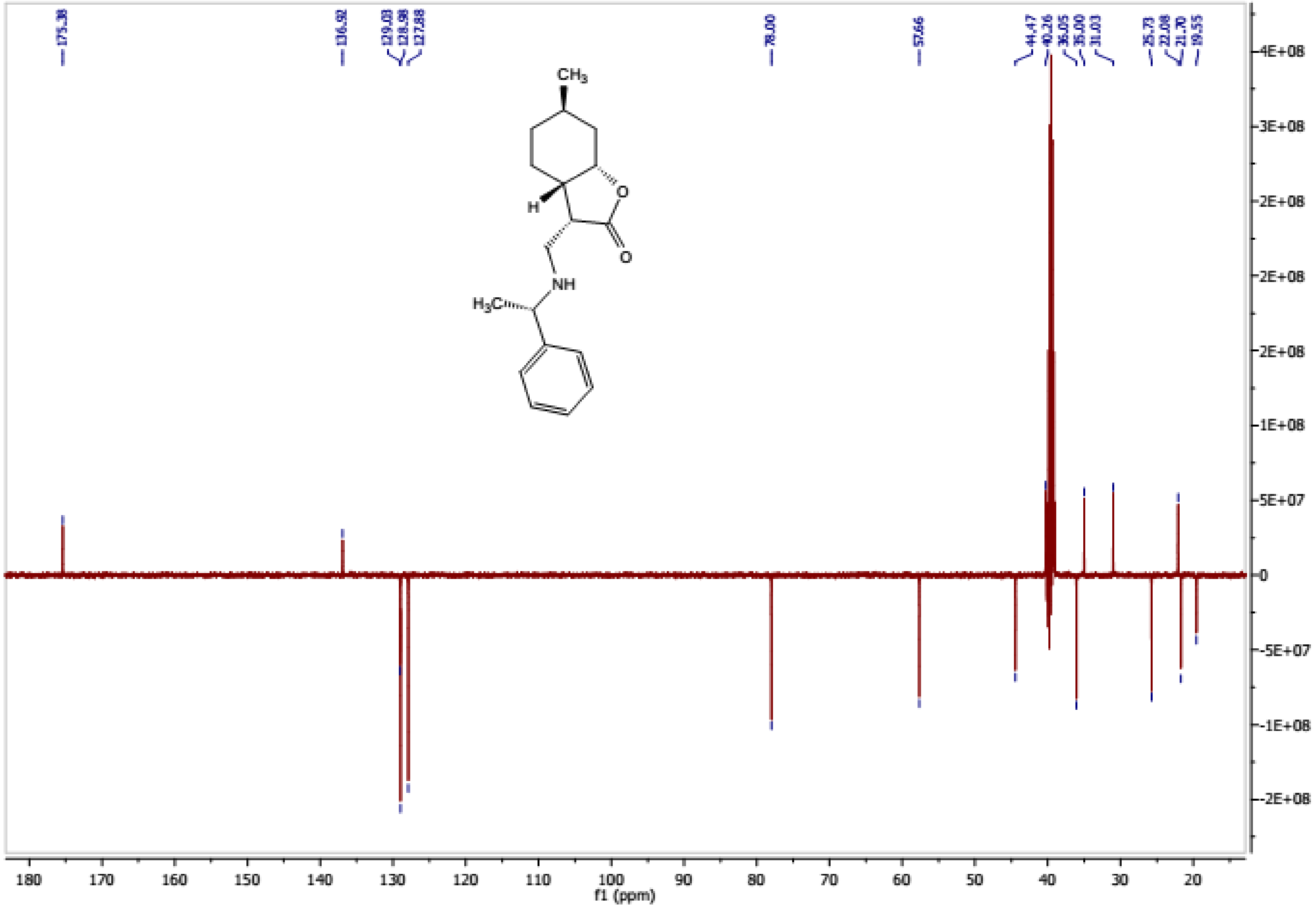

^{1}H-NMR of compound **16**

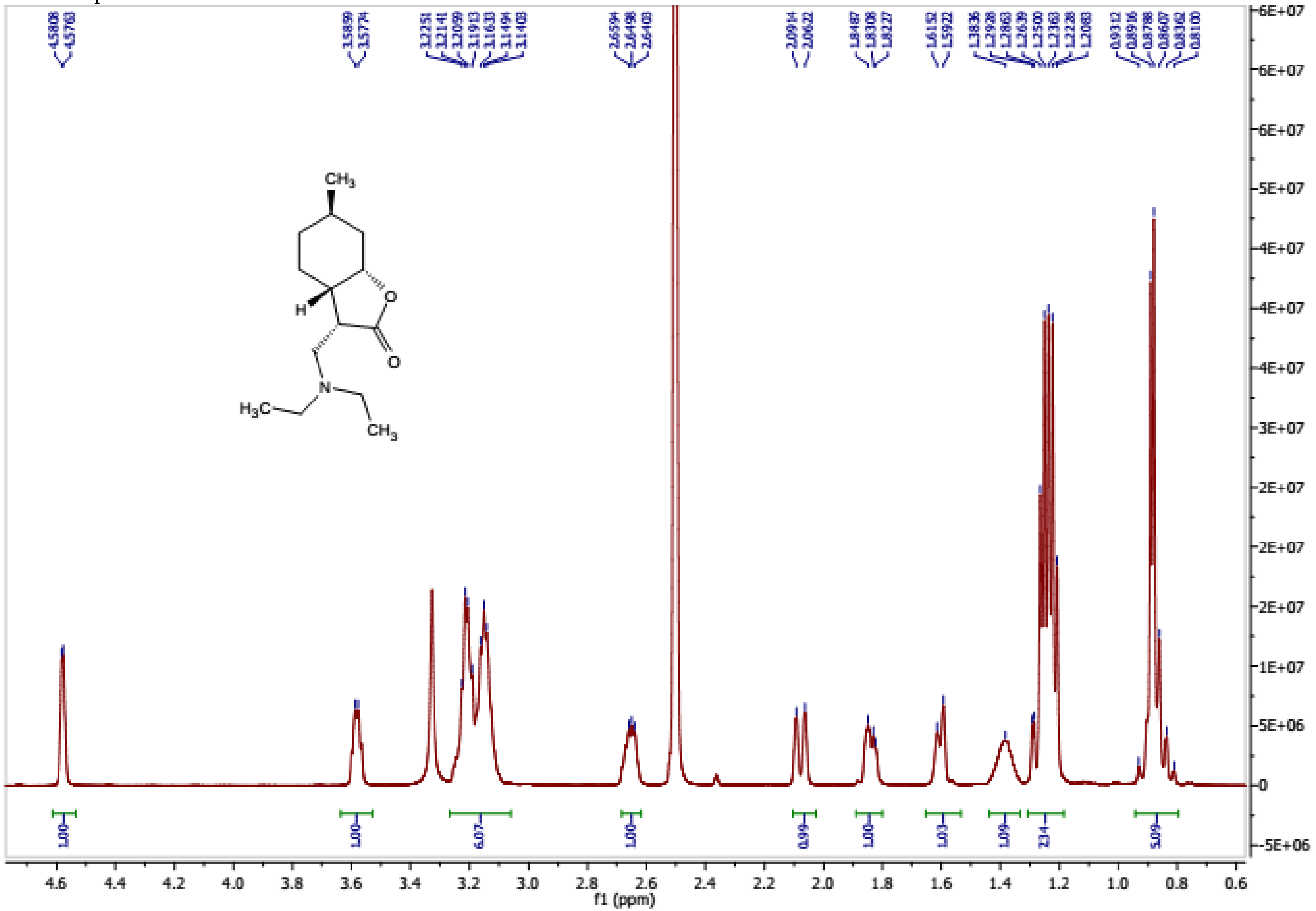

^{13}C-NMR of compound **16**

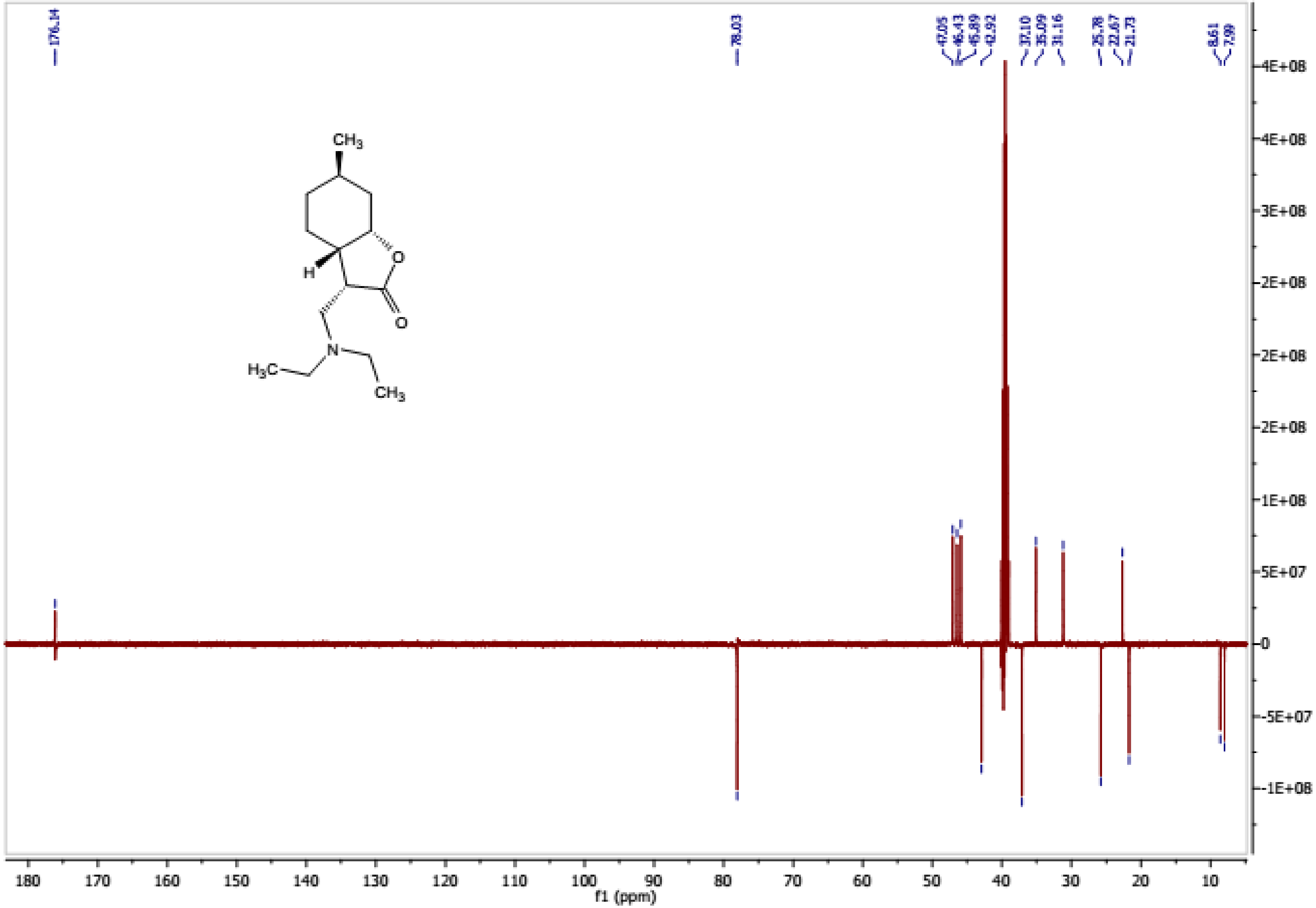

^{1}H-NMR of compound **17**

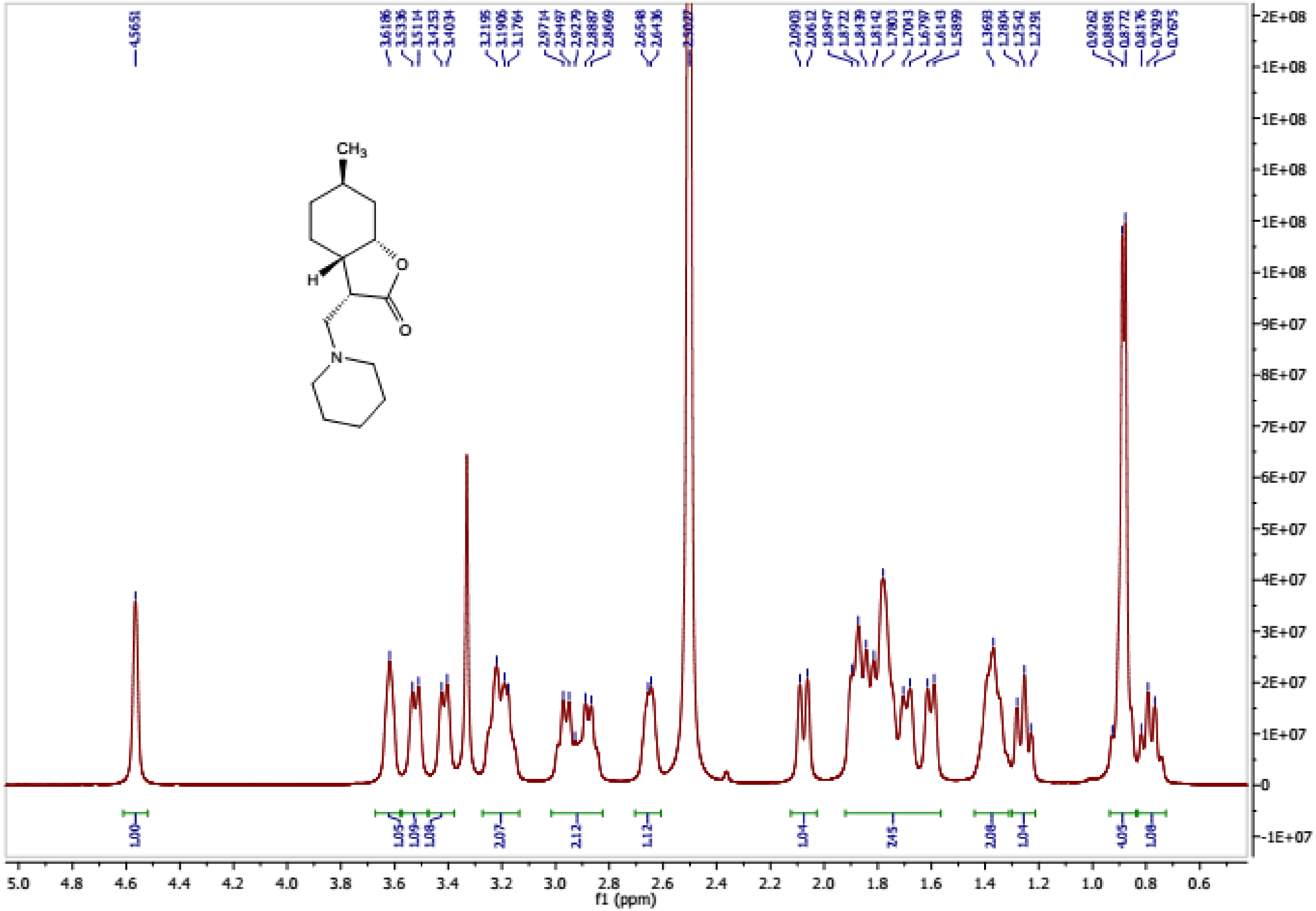

^{13}C-NMR of compound **17**

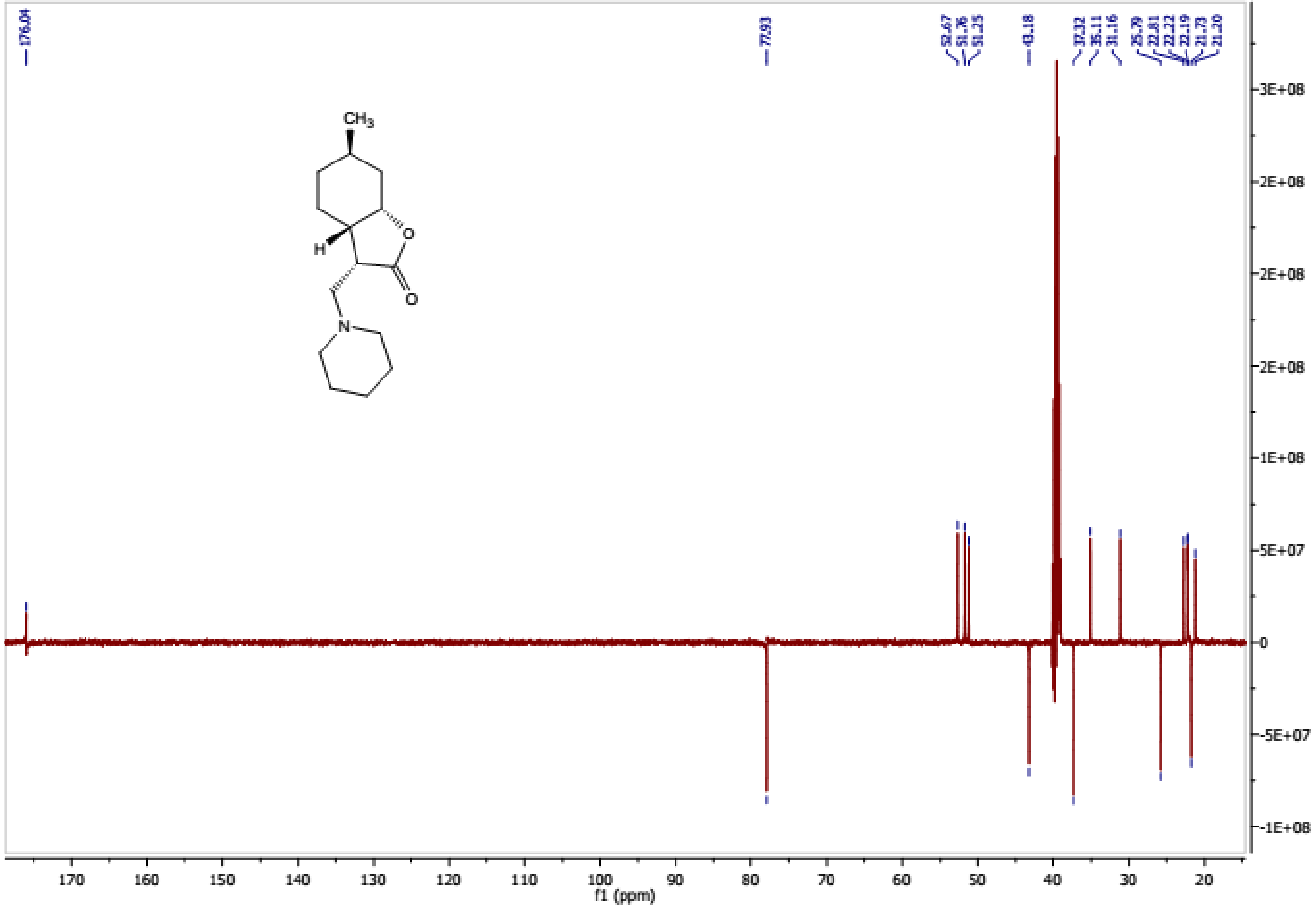

^{1}H-NMR of compound **18**

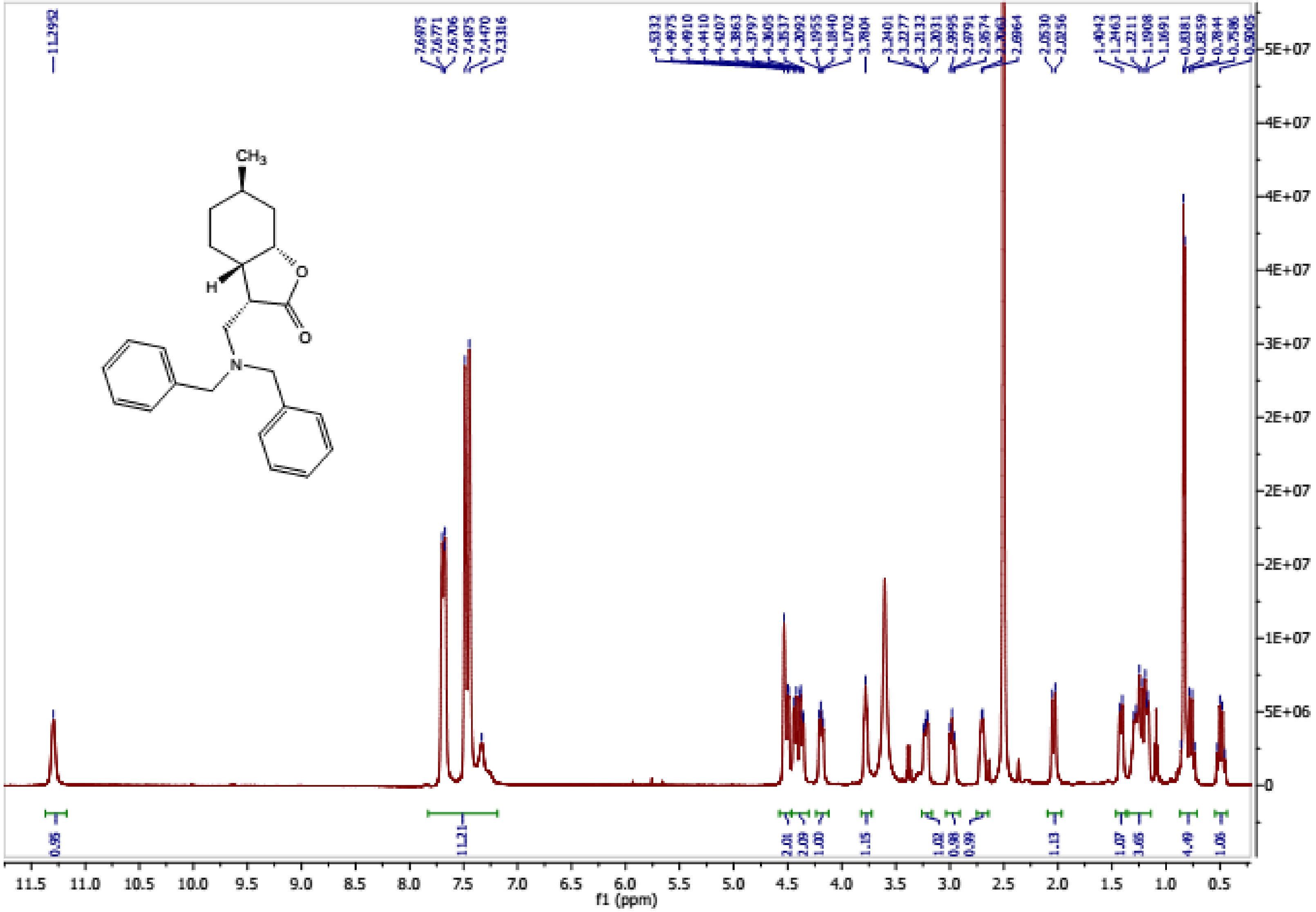

^{13}C-NMR of compound **18**

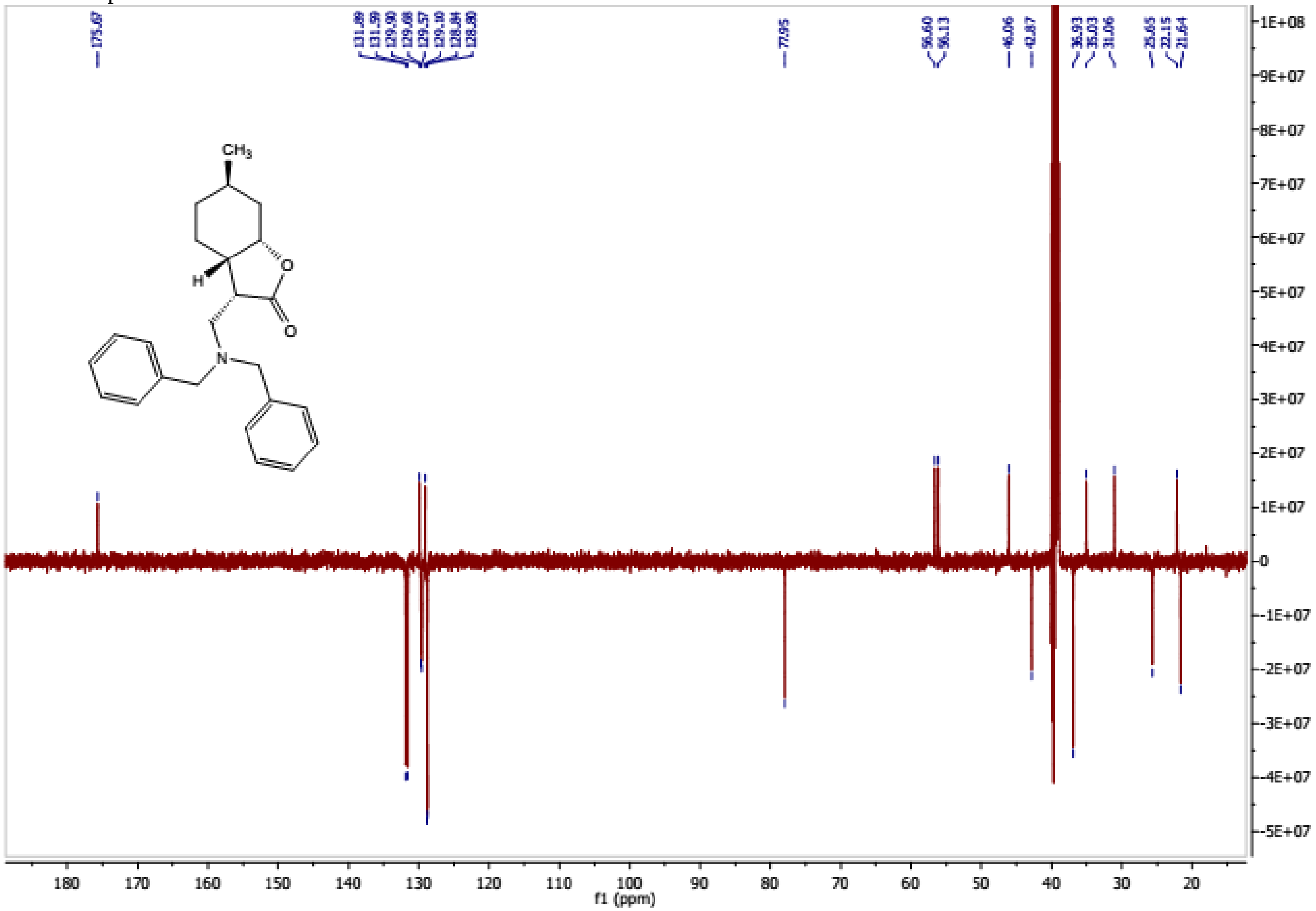

^{1}H-NMR of compound **19**

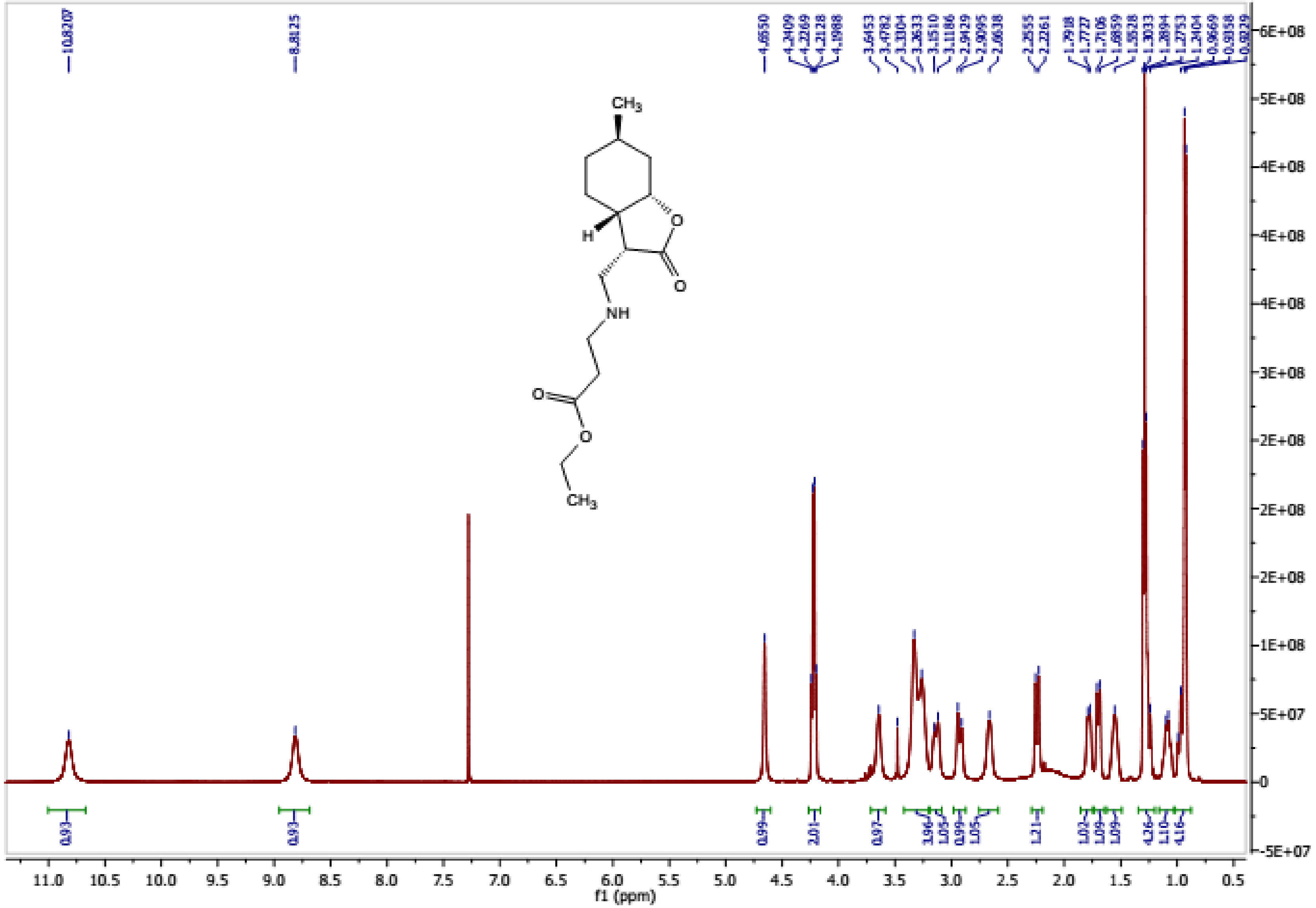

^{13}C-NMR of compound **19**

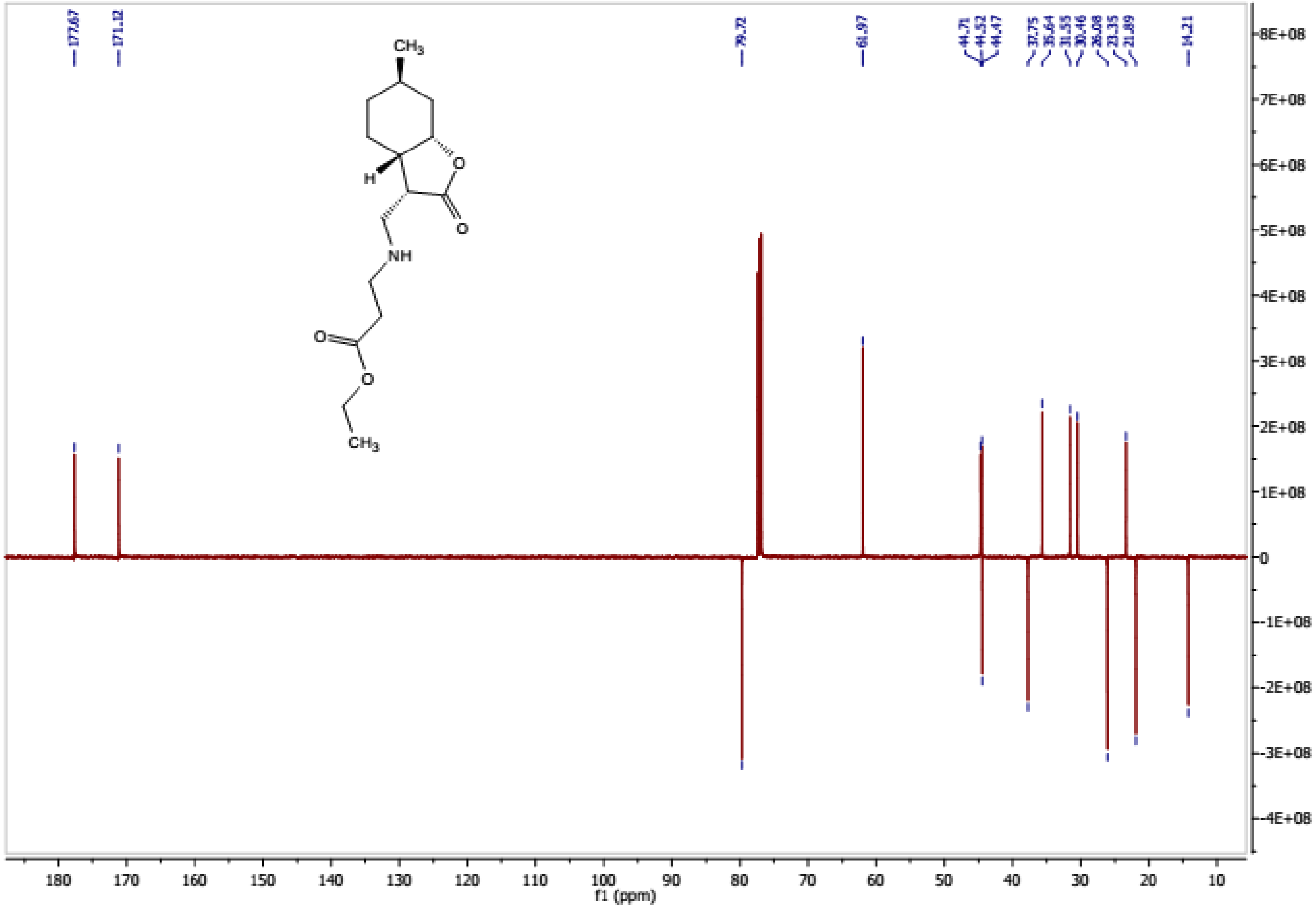

^{1}H-NMR of compound **20**

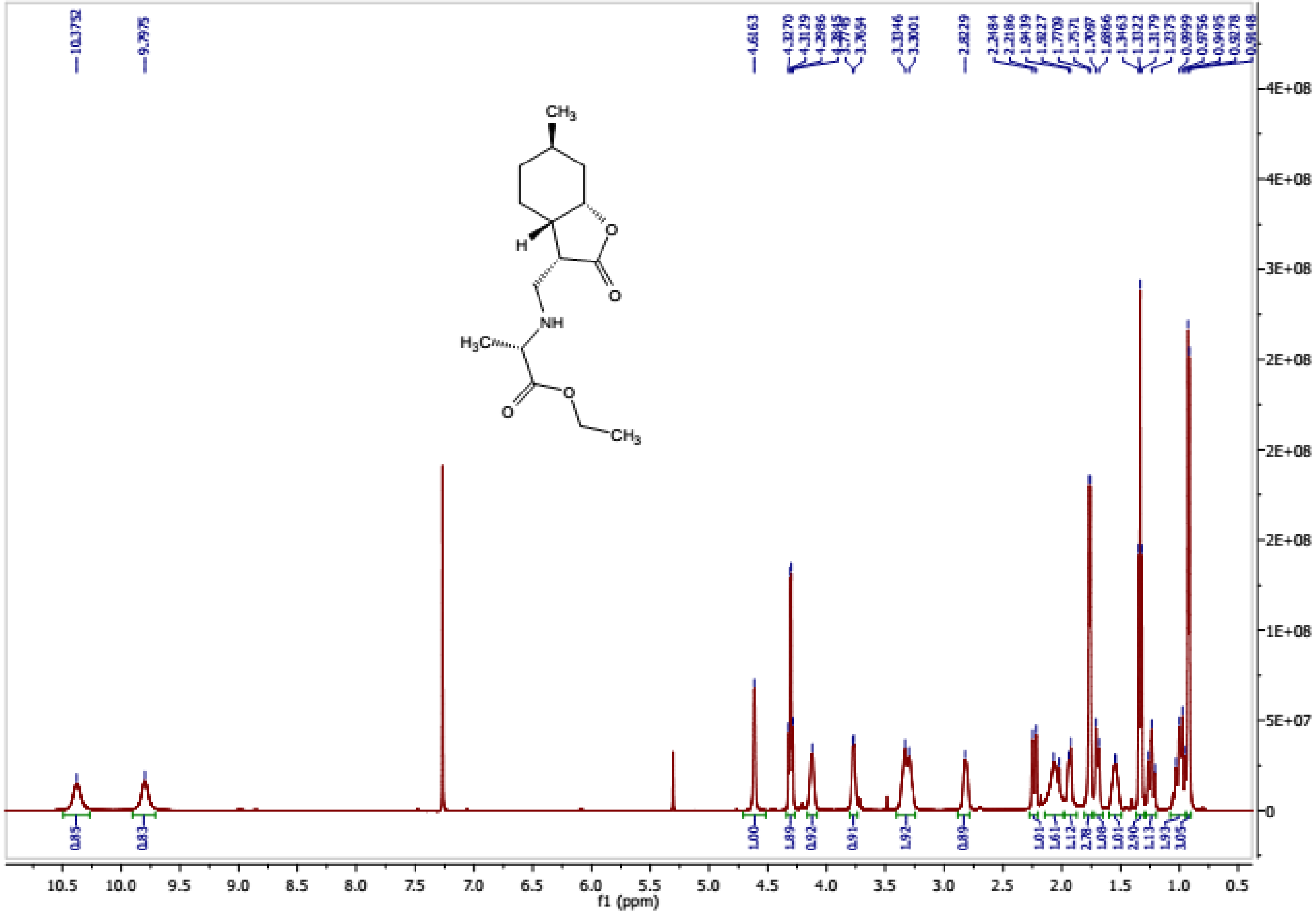

^{13}C-NMR of compound **20**

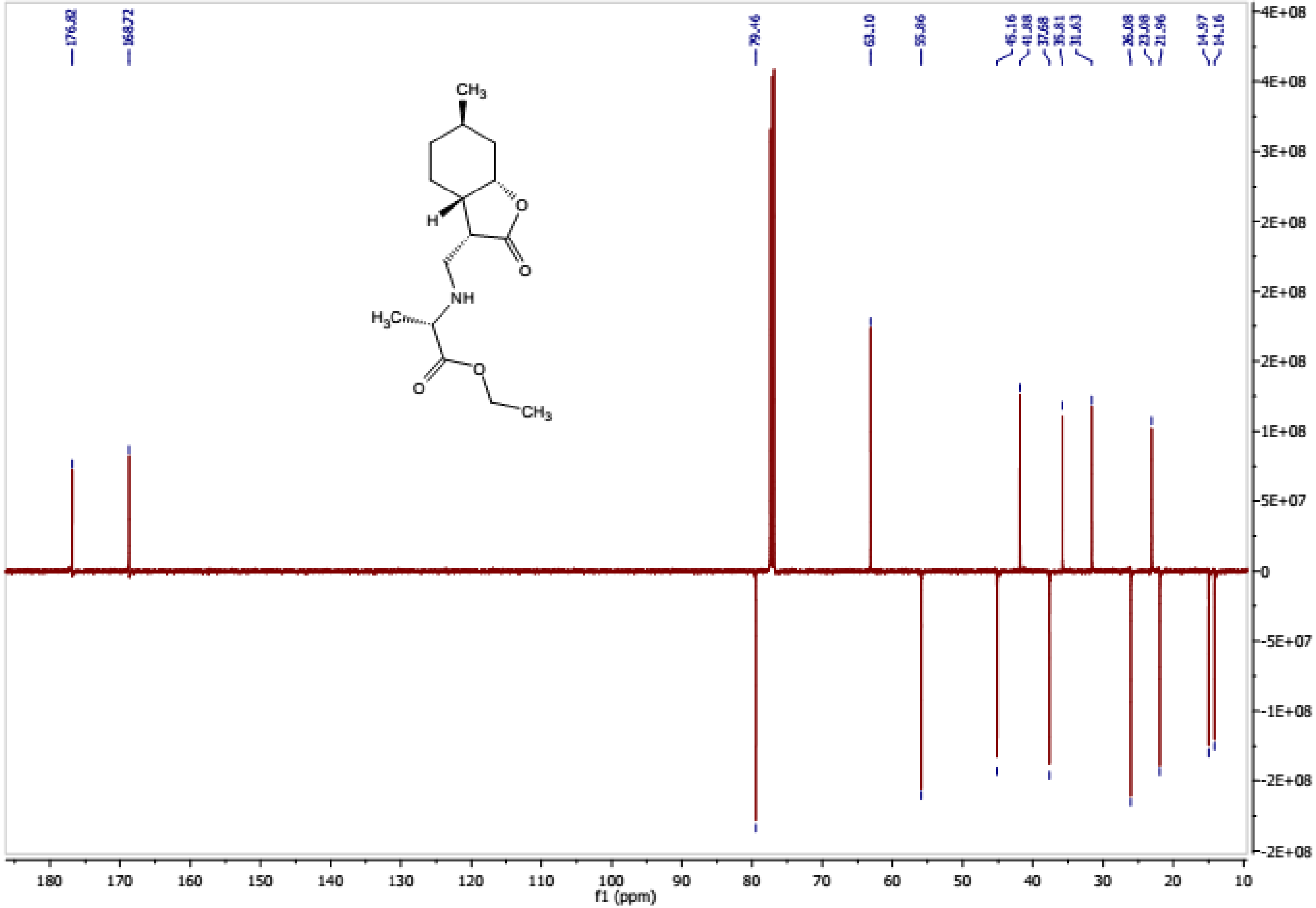

^{1}H-NMR of compound **21**

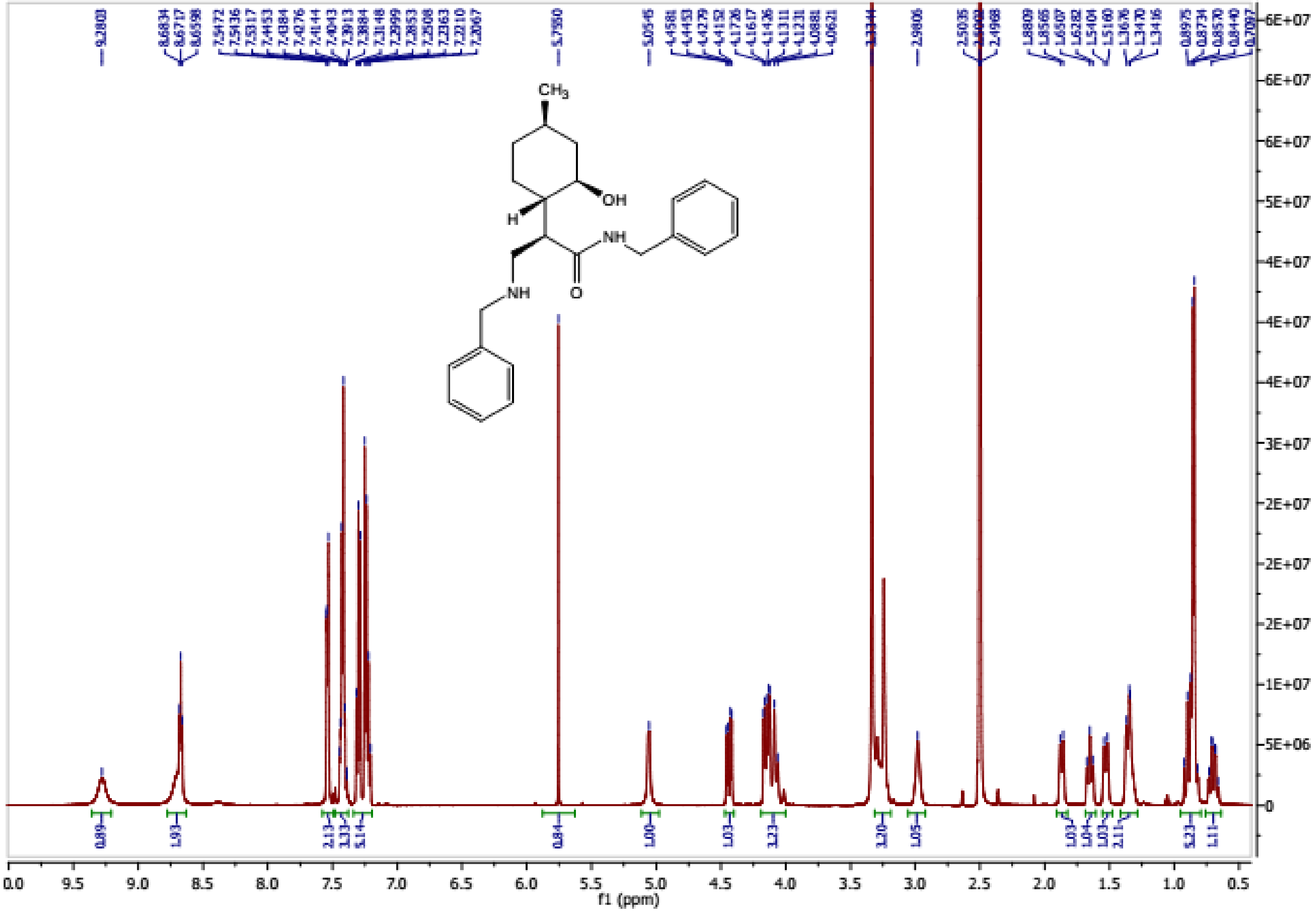

^{13}C-NMR of compound **21**

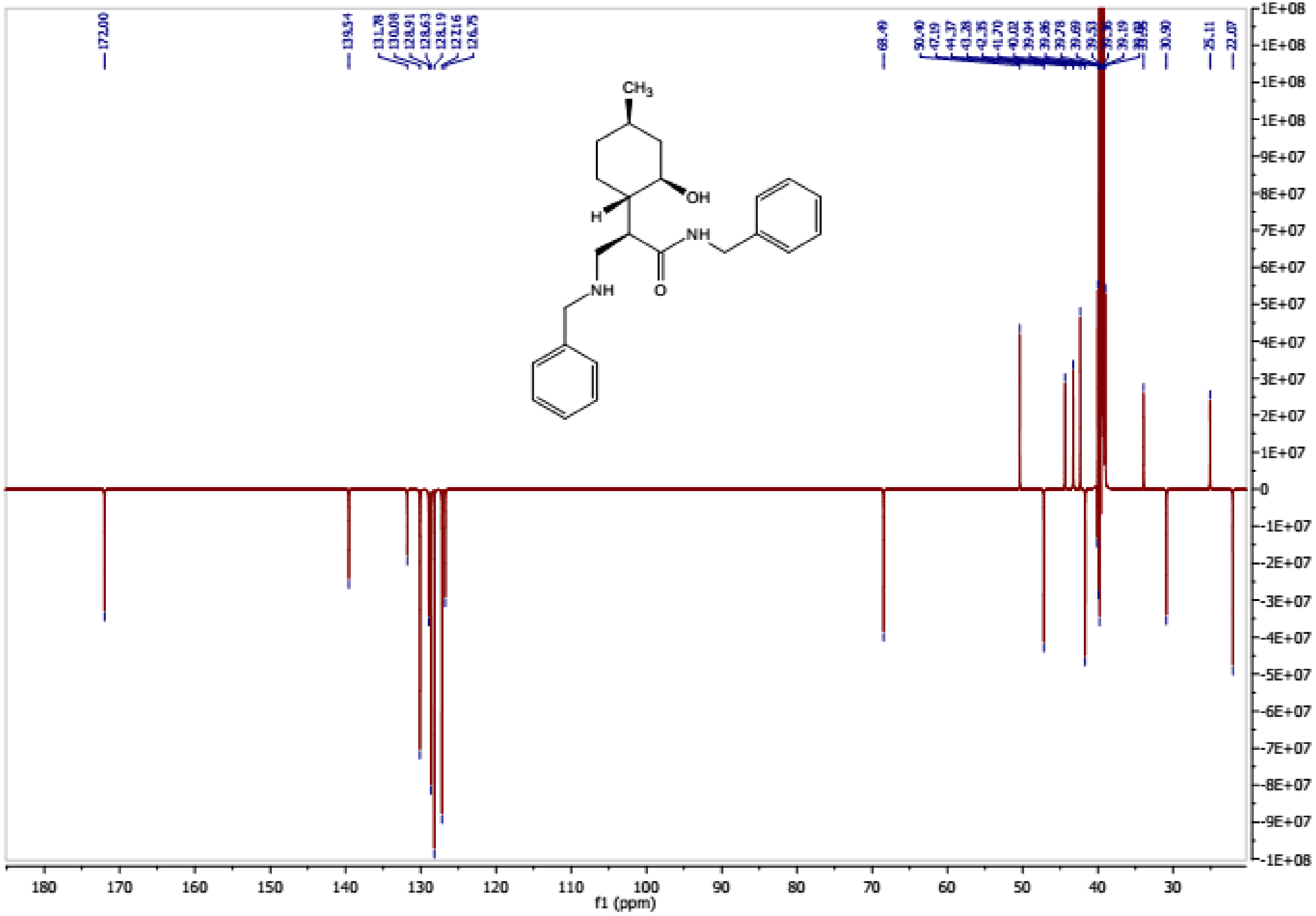

^{1}H-NMR of compound **22**

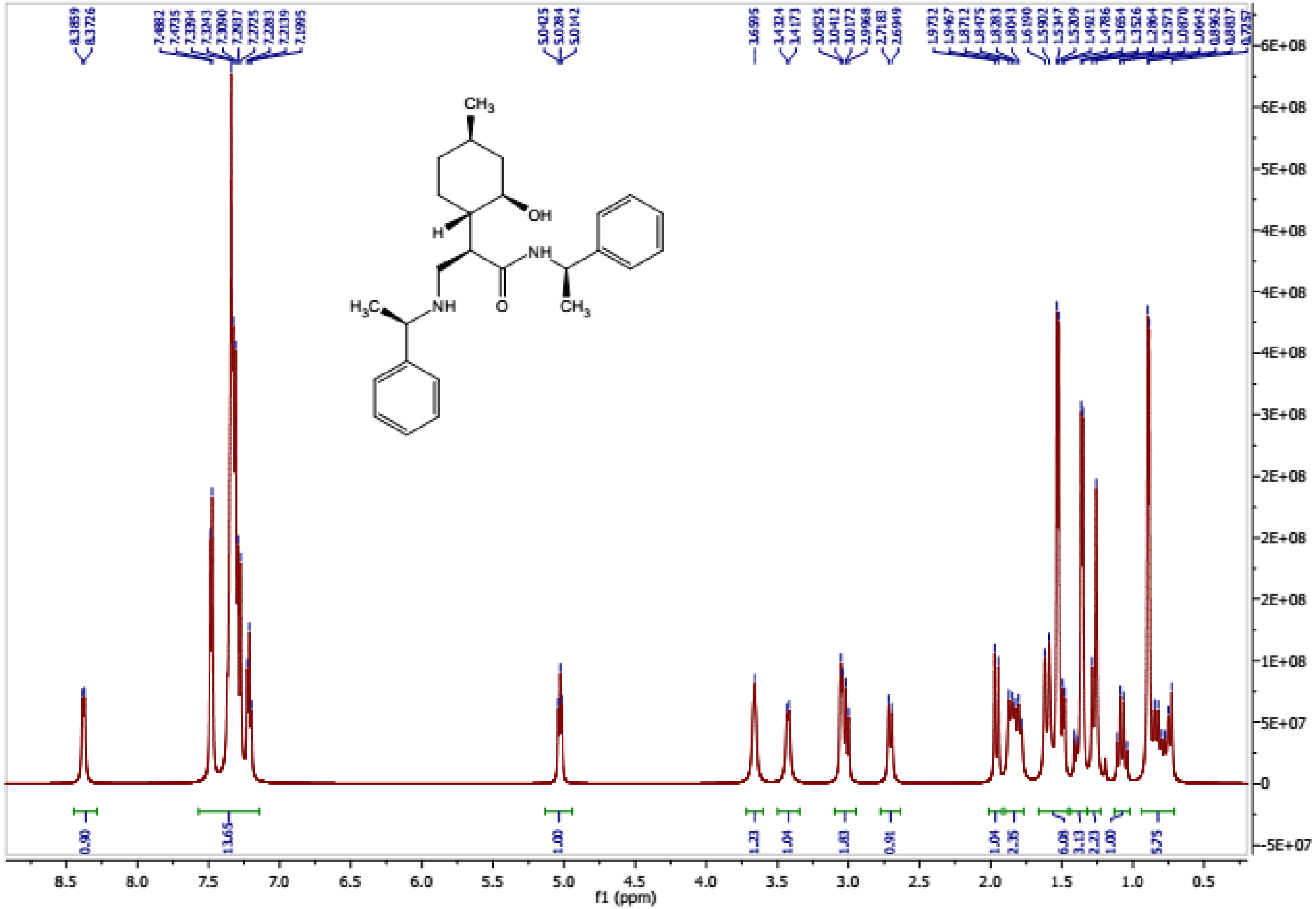

^{13}C-NMR of compound **22**

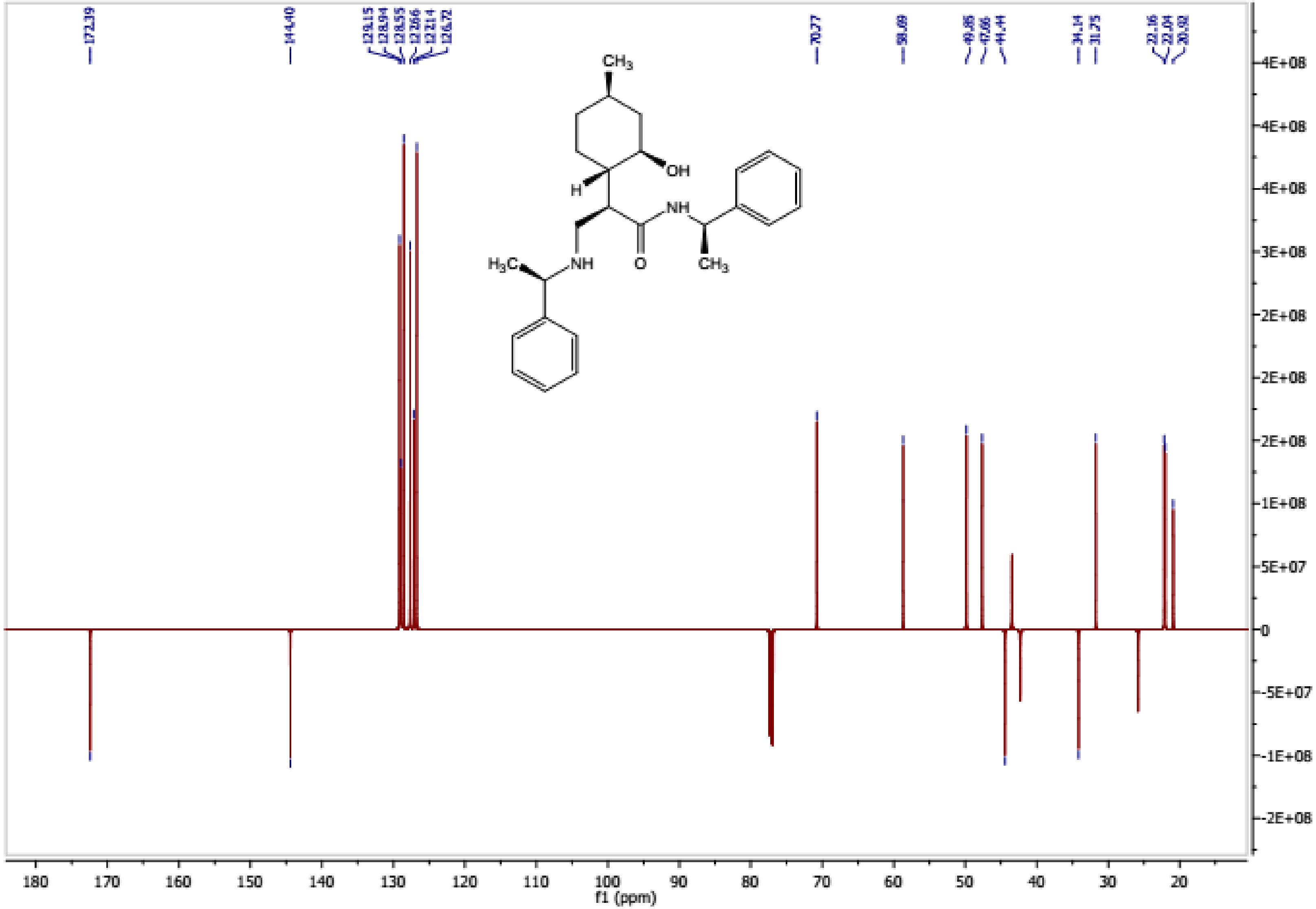

^{1}H-NMR of compound **23**

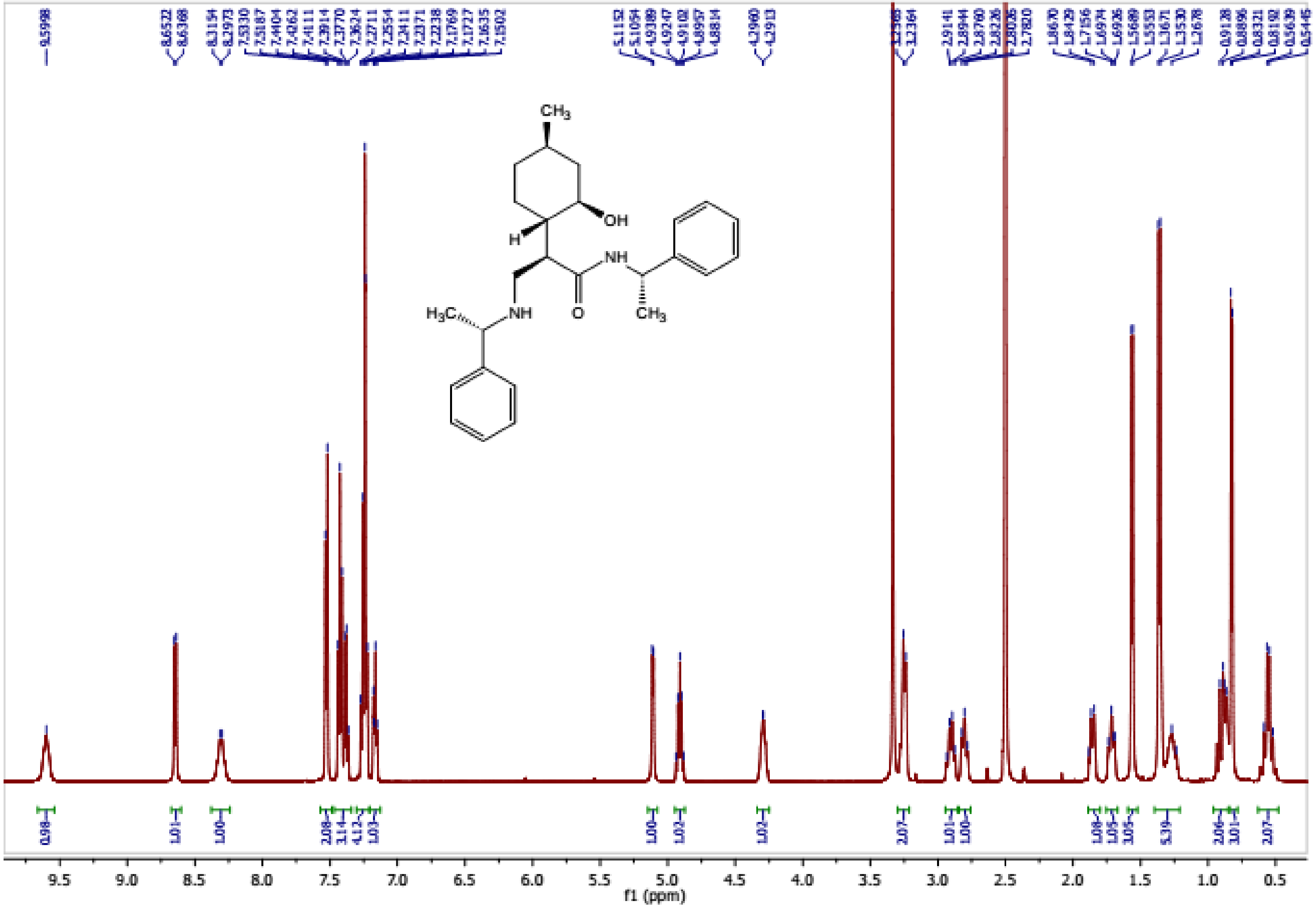

^{13}C-NMR of compound **23**

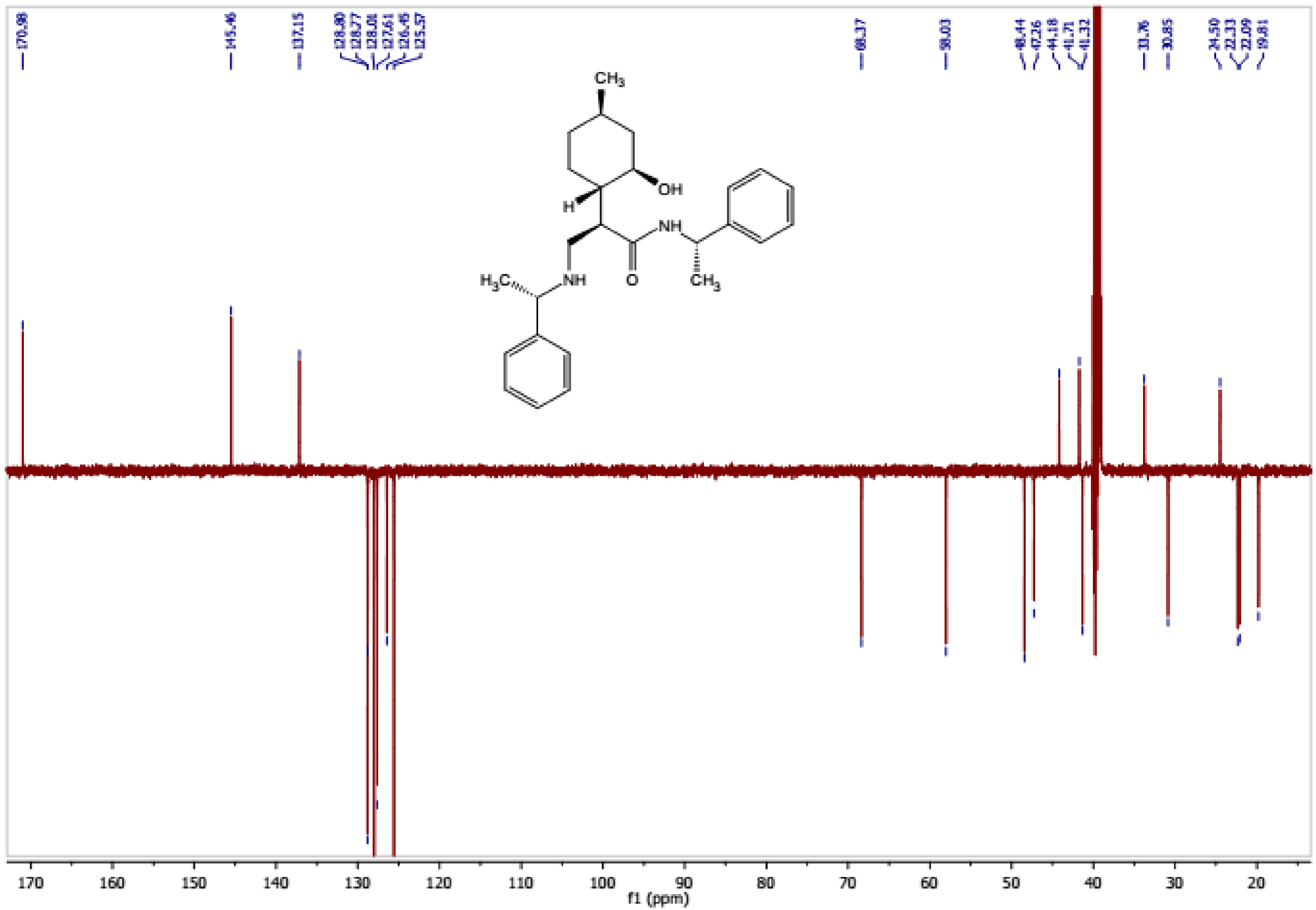

^{1}H-NMR of compound **24**

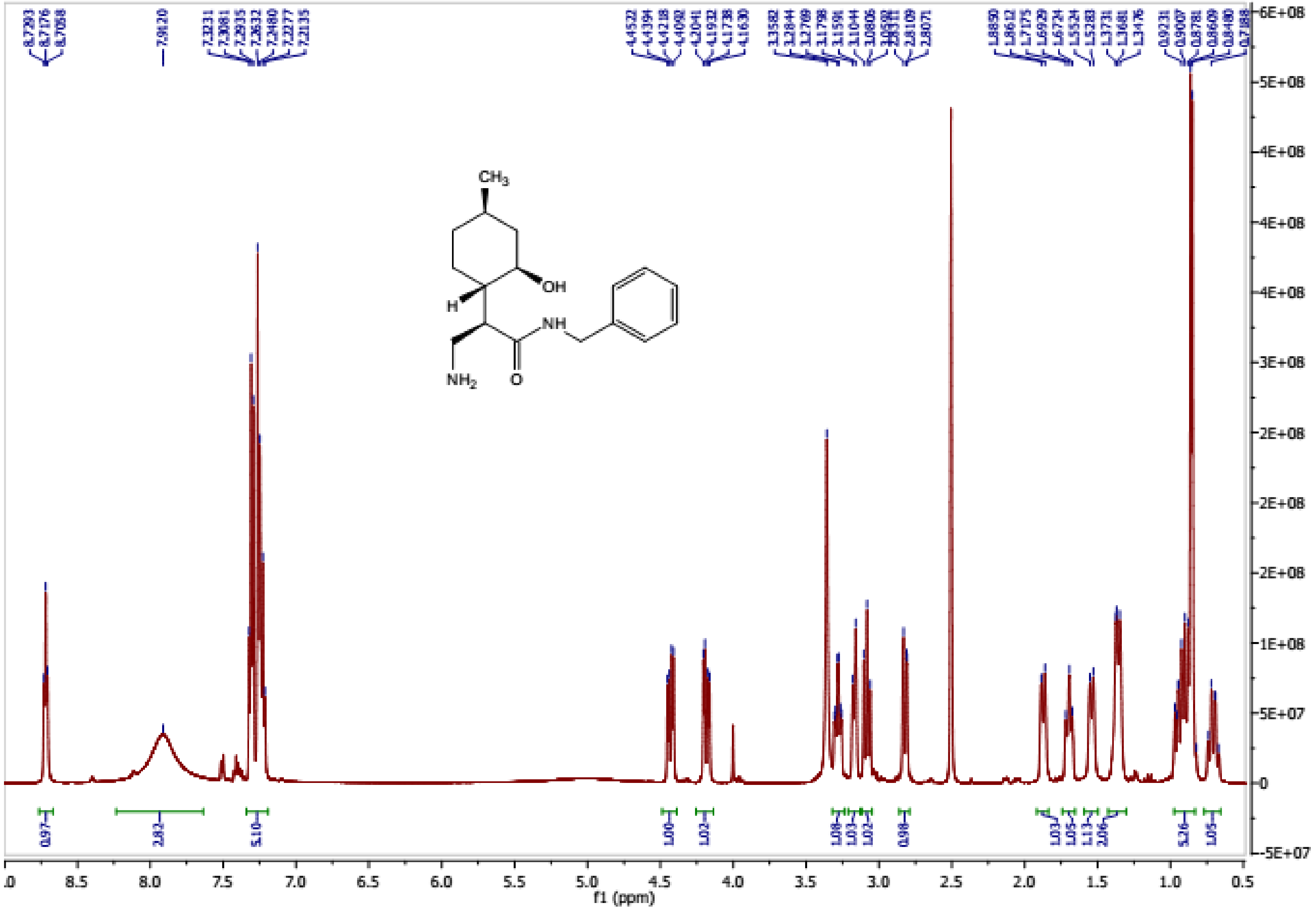

^{13}C-NMR of compound **24**

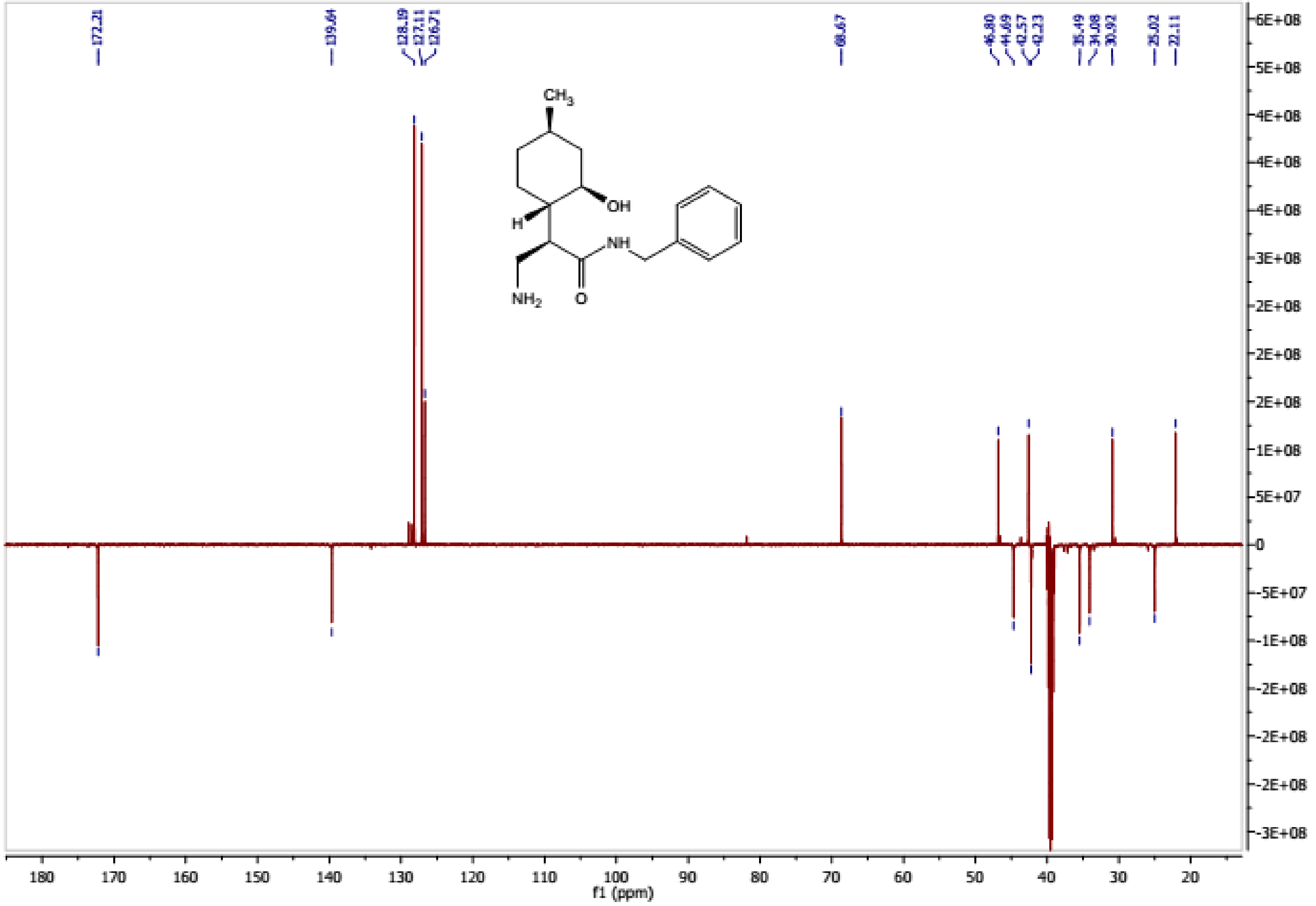

^{1}H-NMR of compound **25**

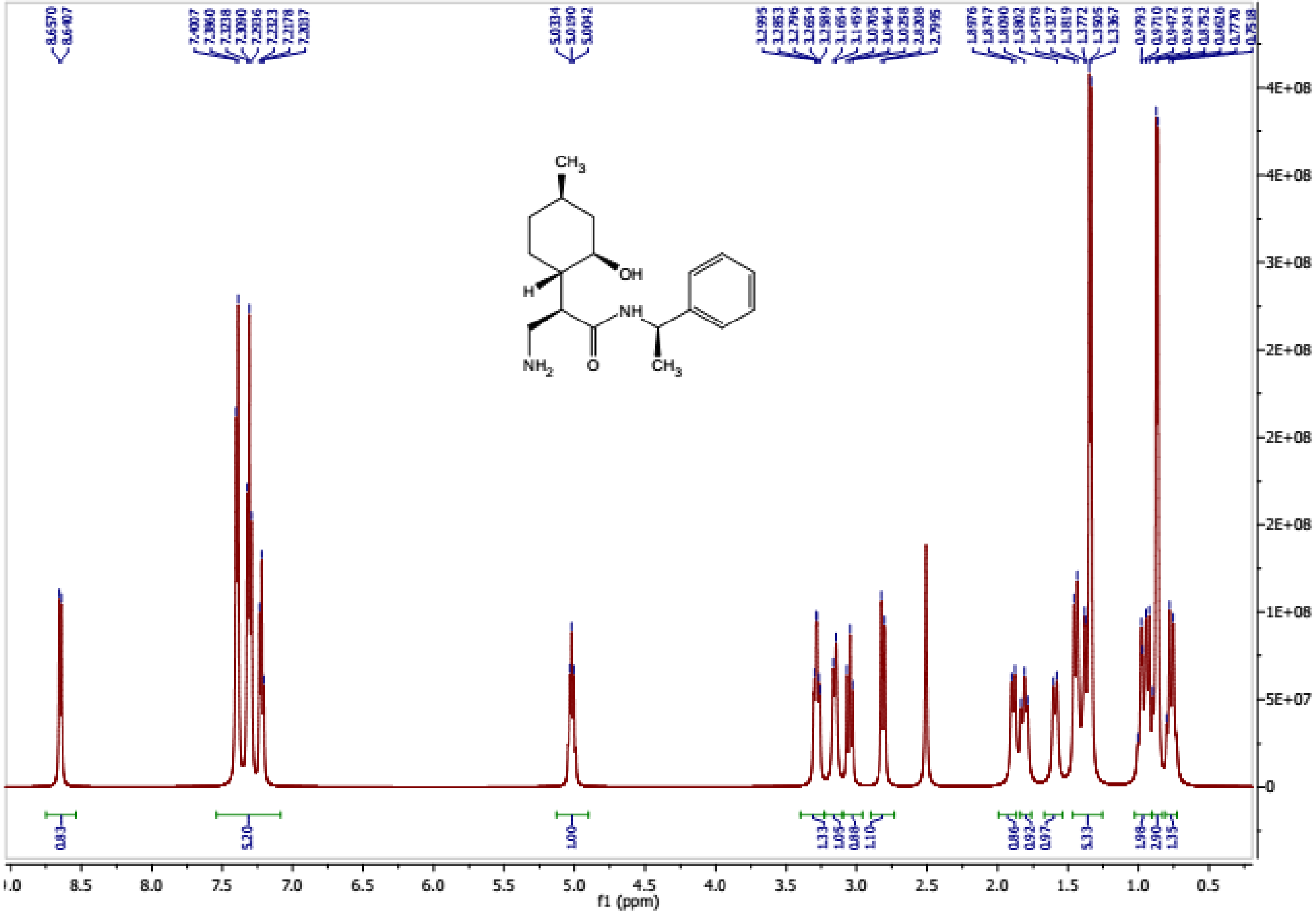

^{13}C-NMR of compound **25**

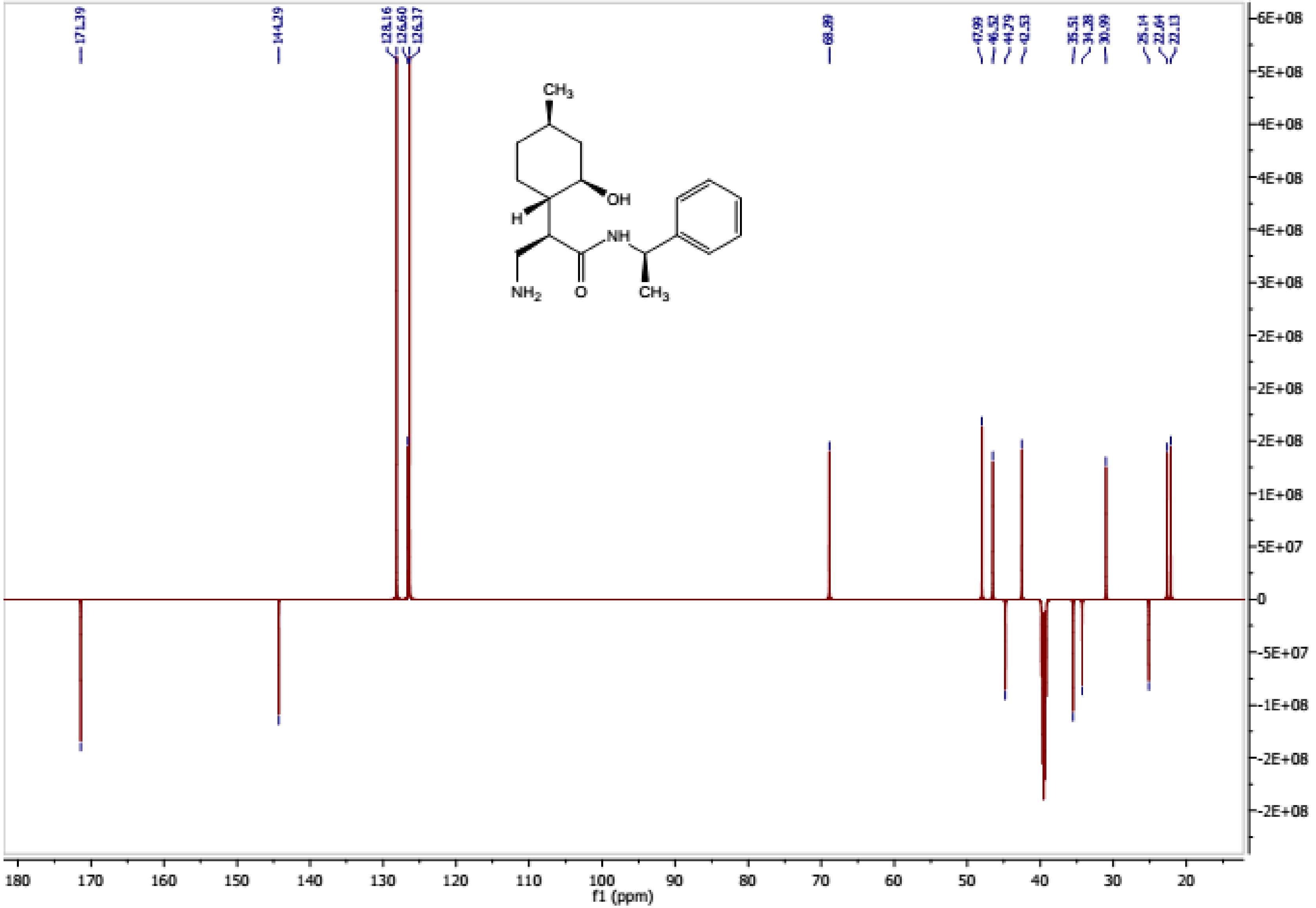

^{1}H-NMR of compound **26**

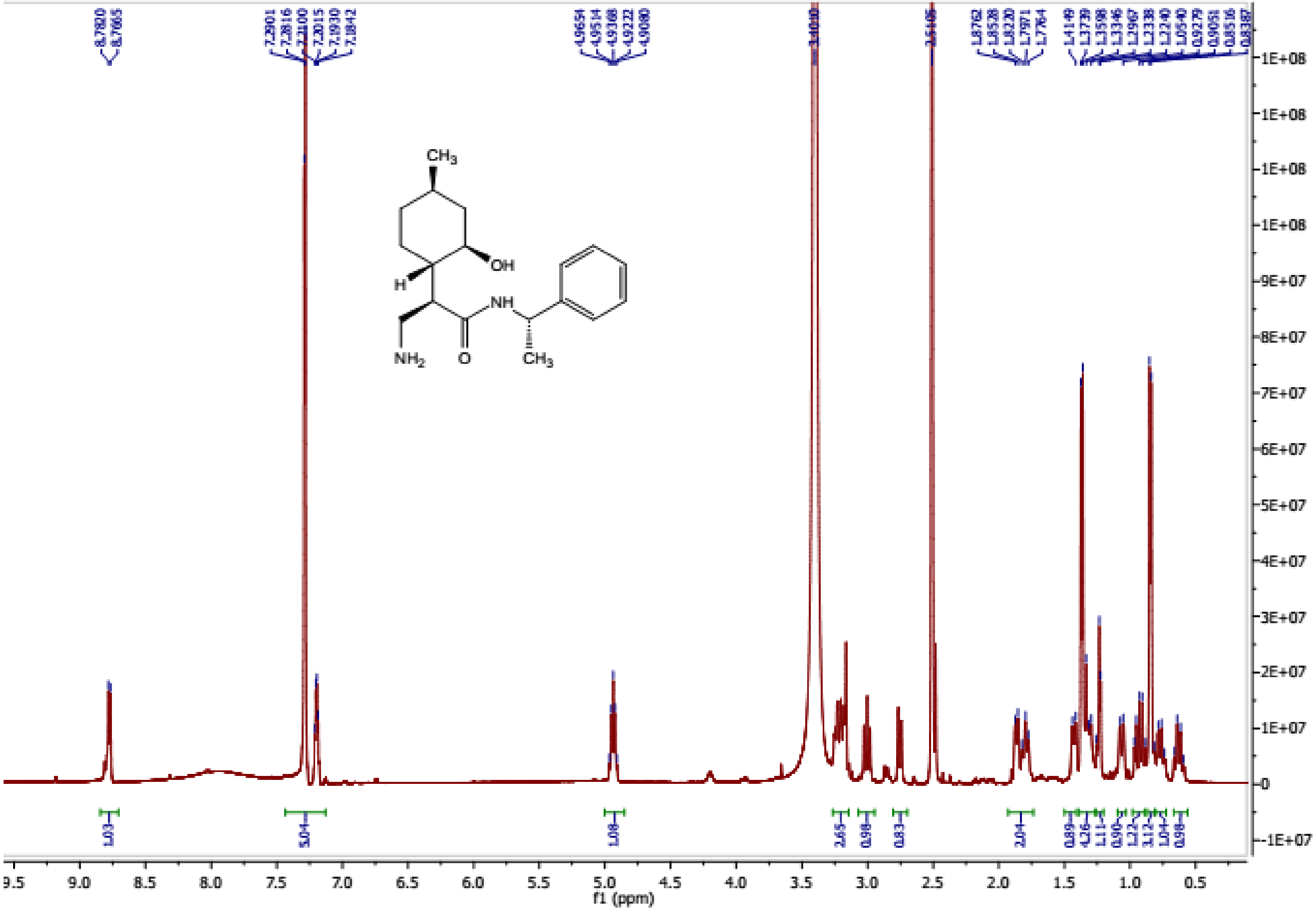

^{13}C-NMR of compound **26**

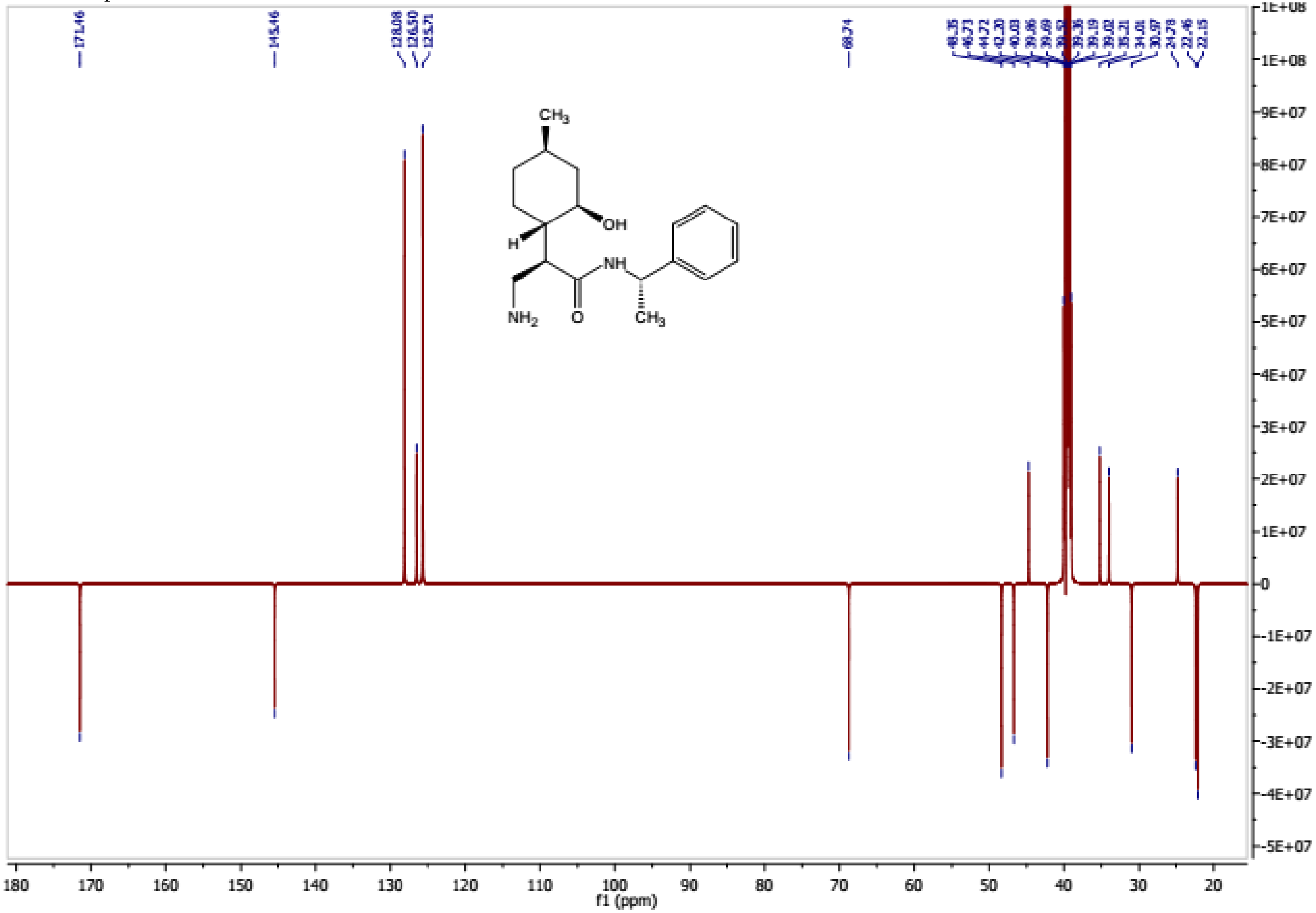

^{1}H-NMR of compound **27**

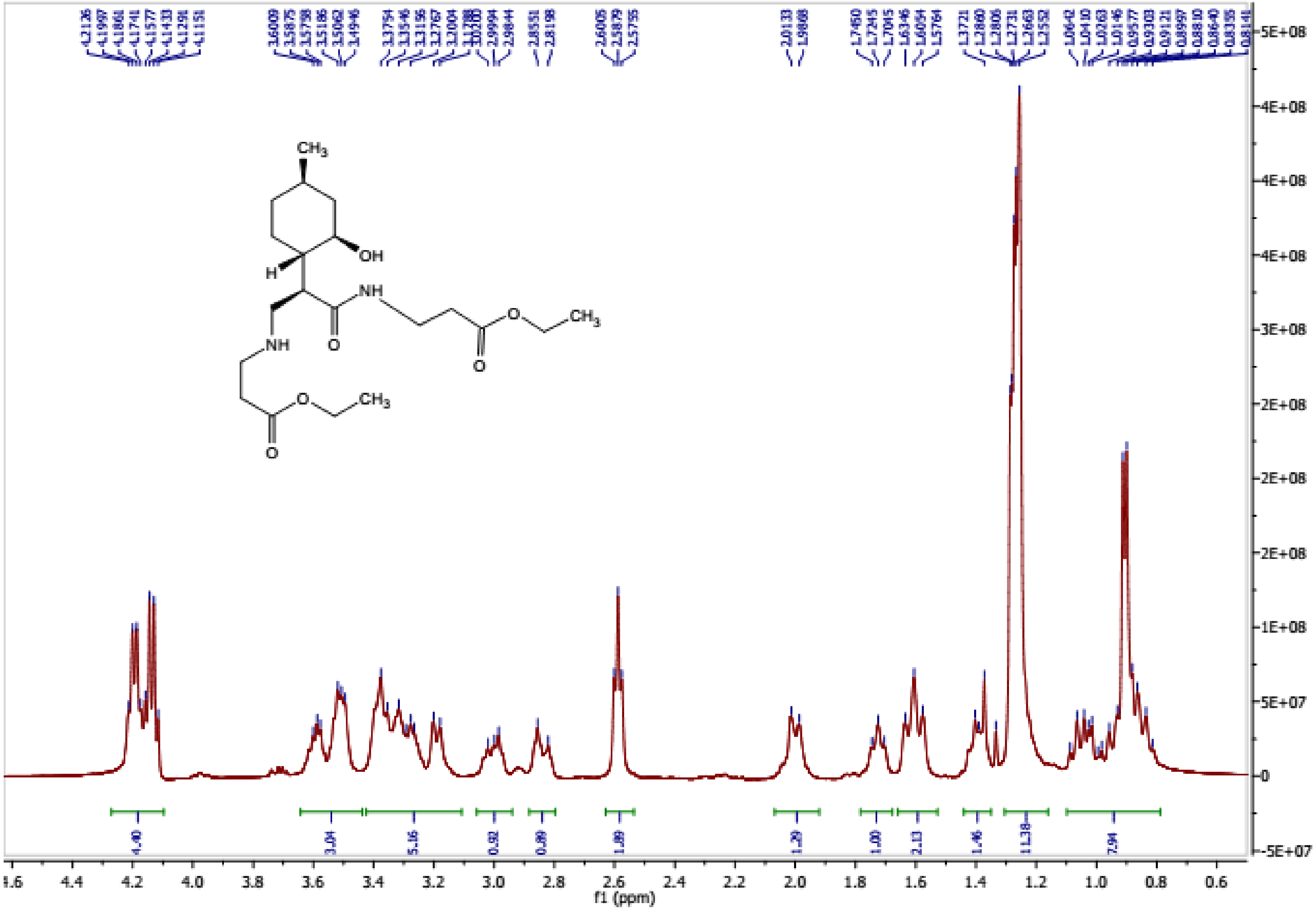

^{13}C-NMR of compound **27**

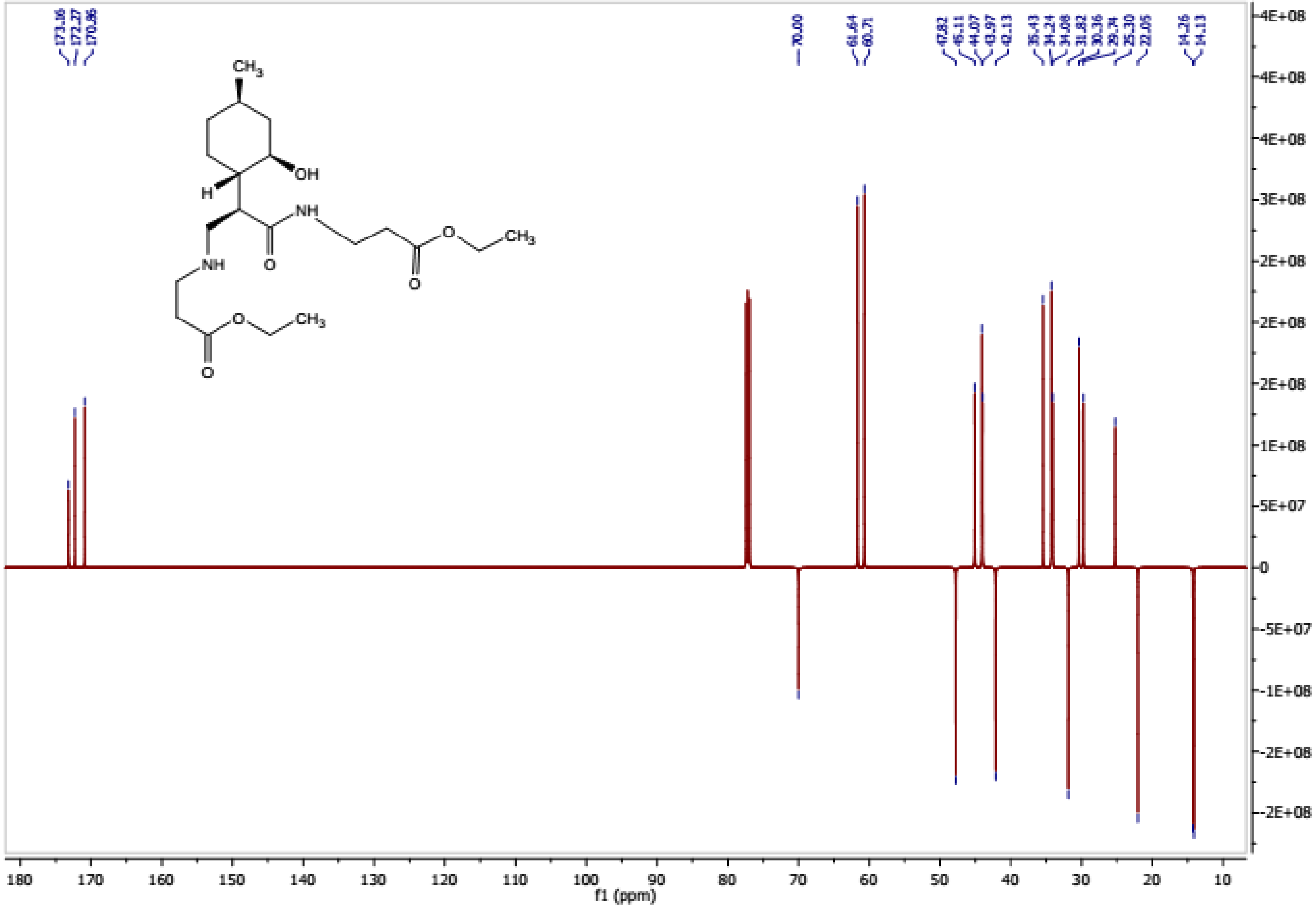

^{1}H-NMR of compound **28**

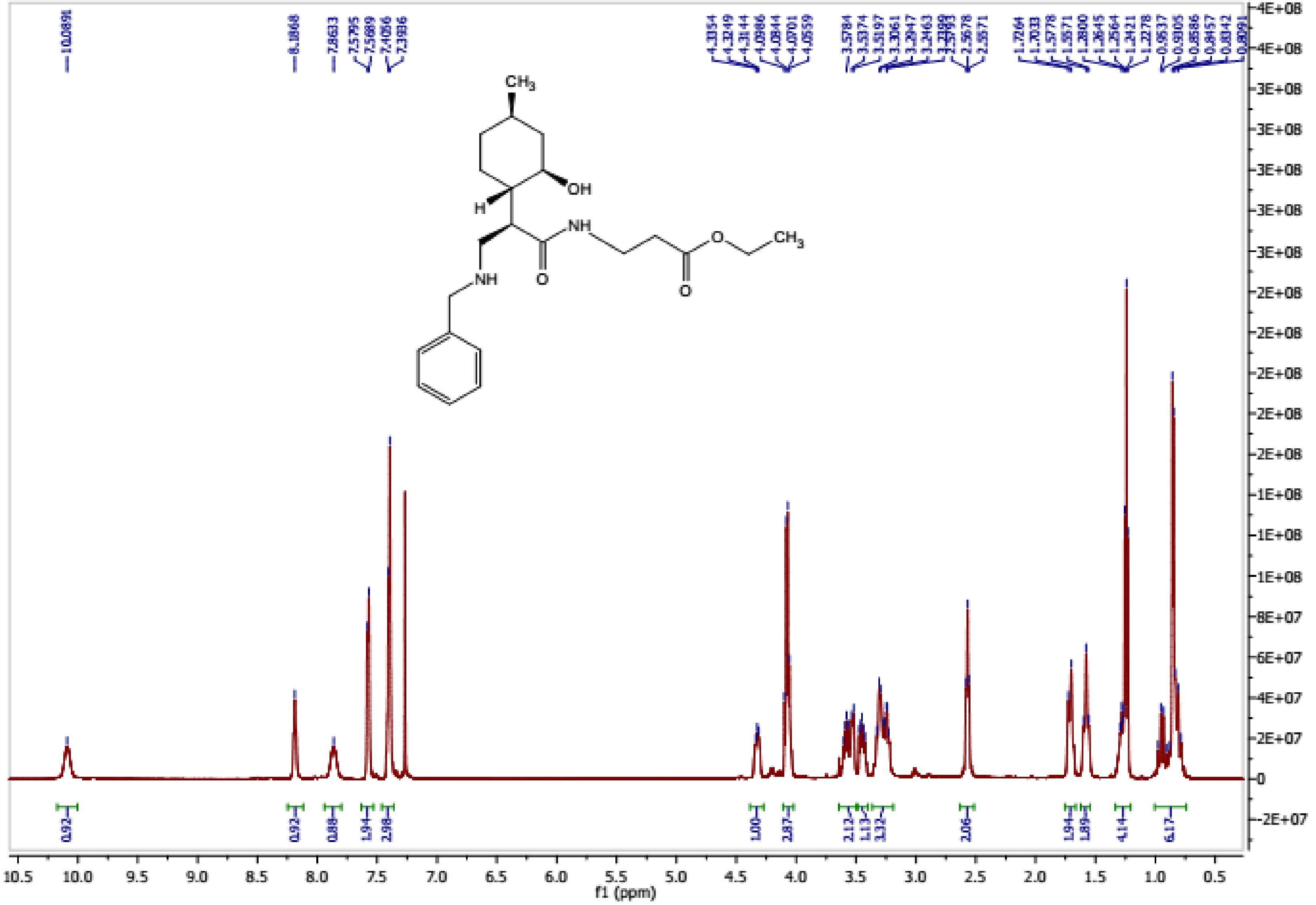

^{13}C-NMR of compound **28**

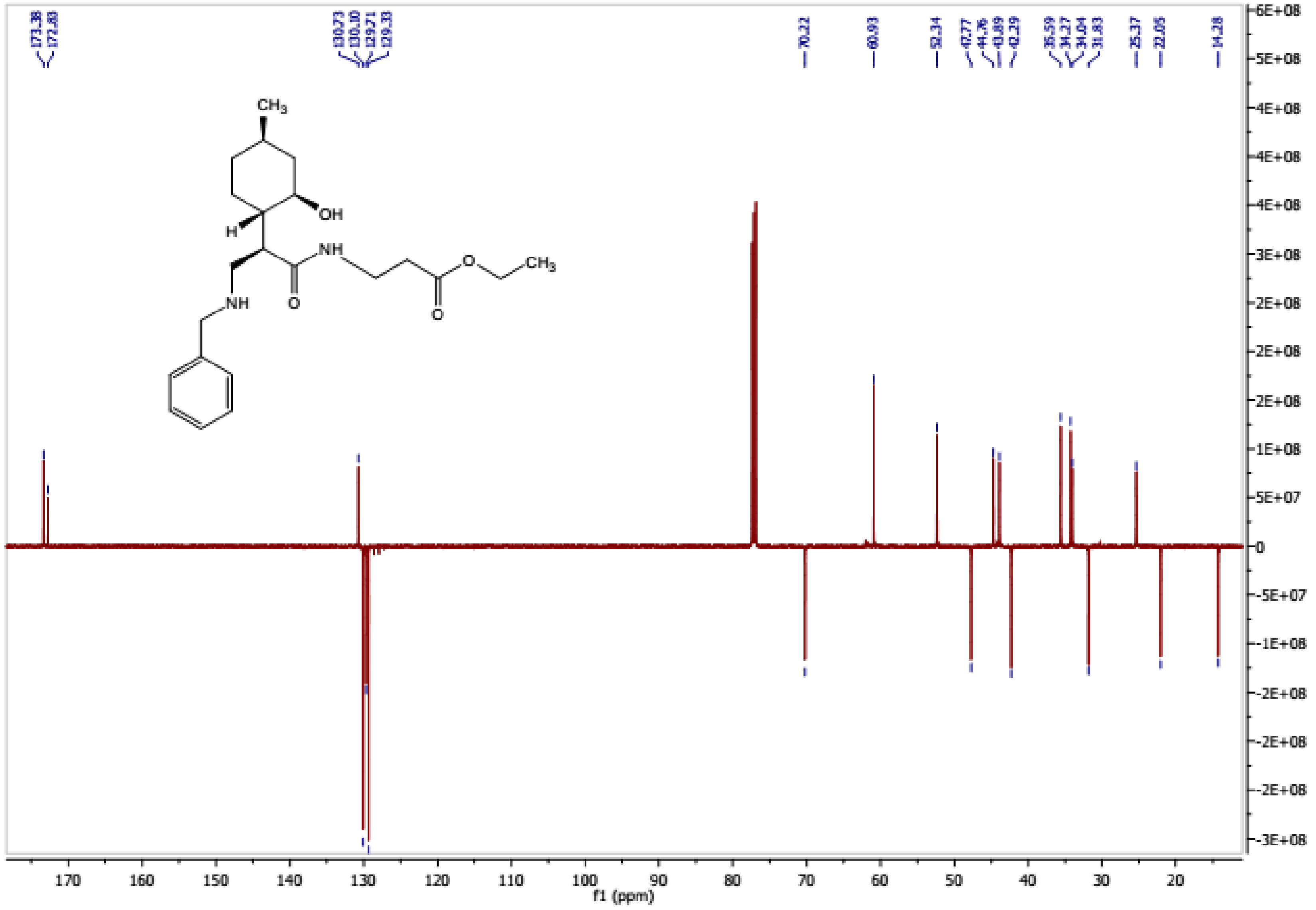

^{1}H-NMR of compound **29**

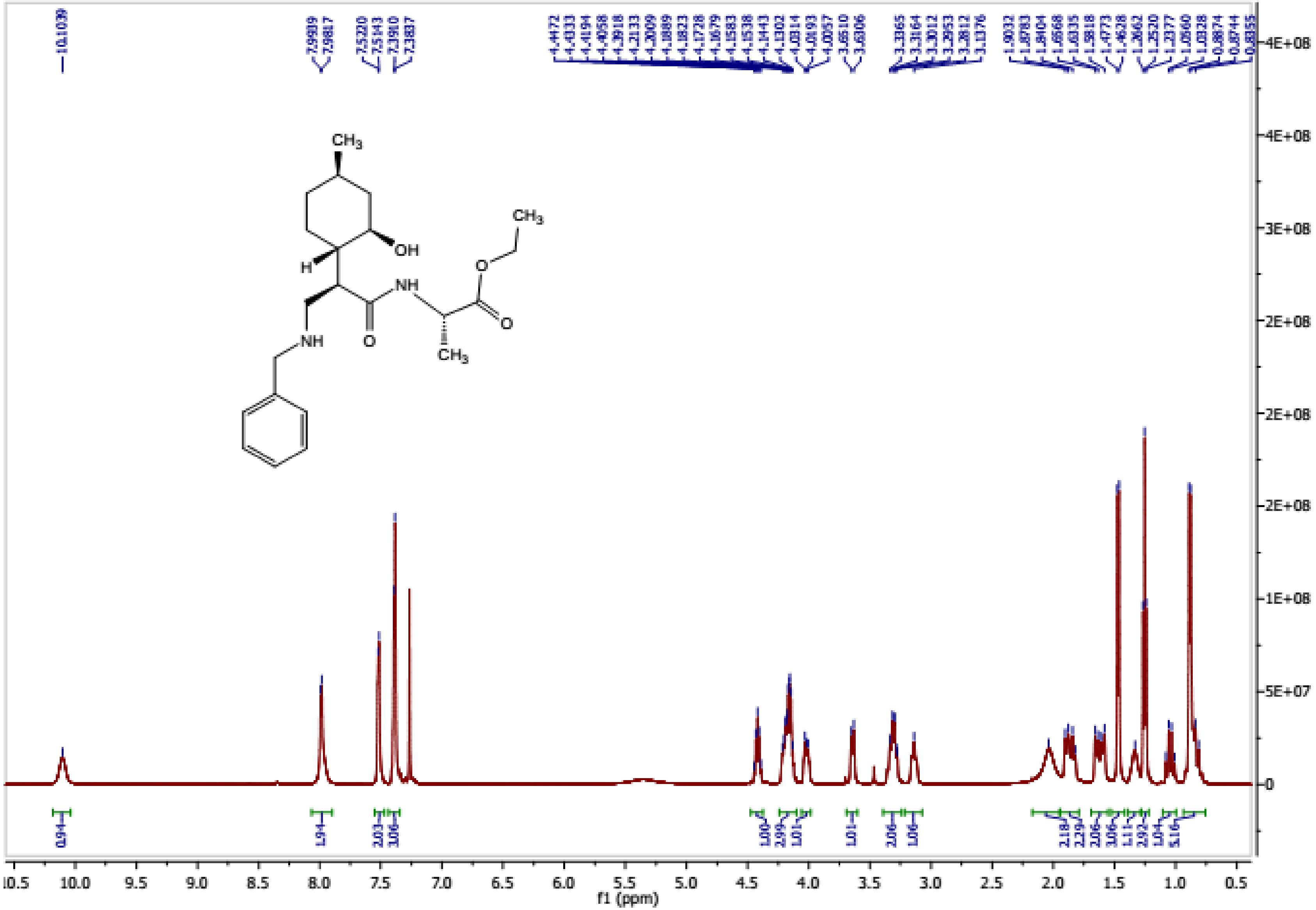

^{13}C-NMR of compound **29**

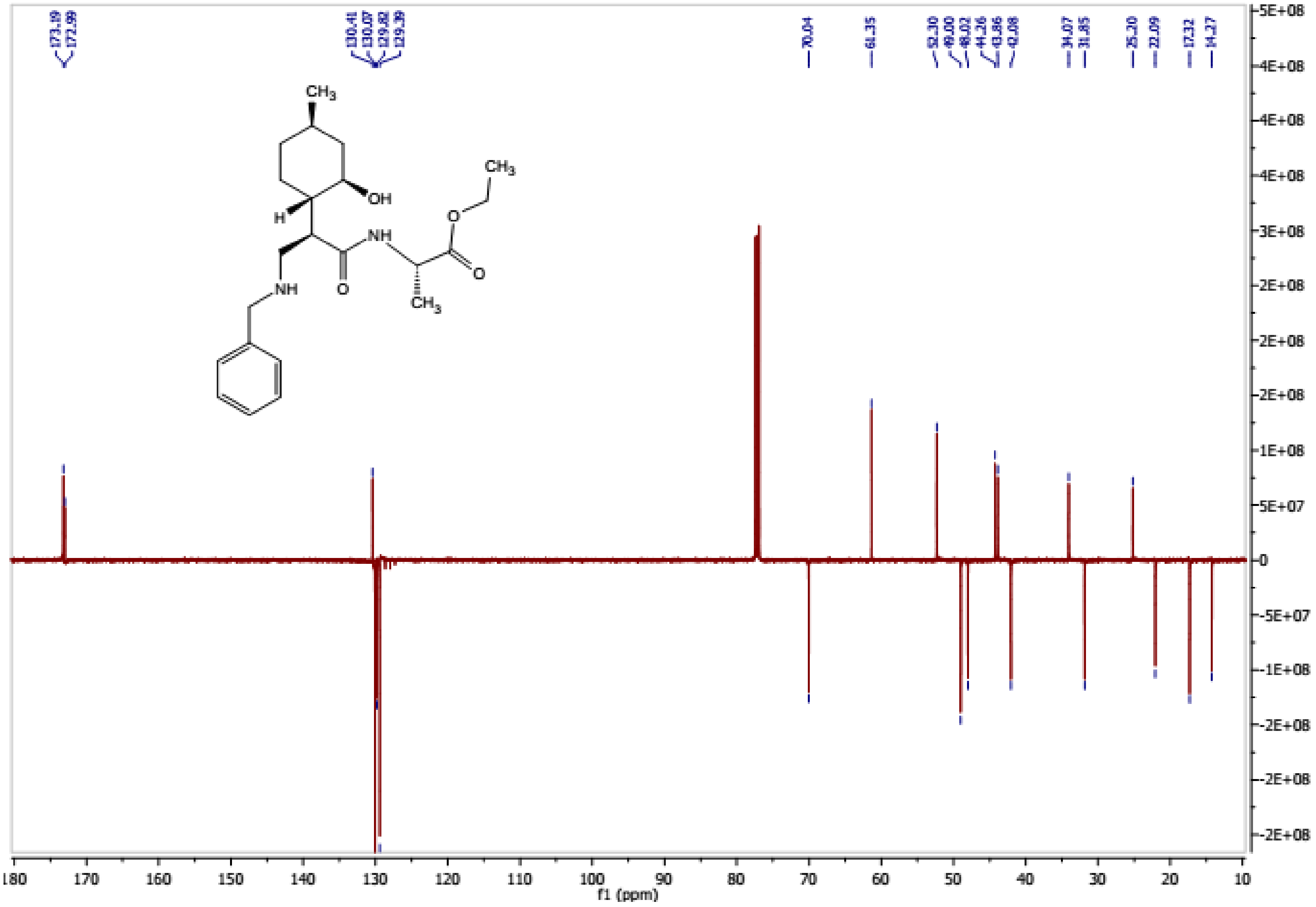

^{1}H-NMR of compound **30**

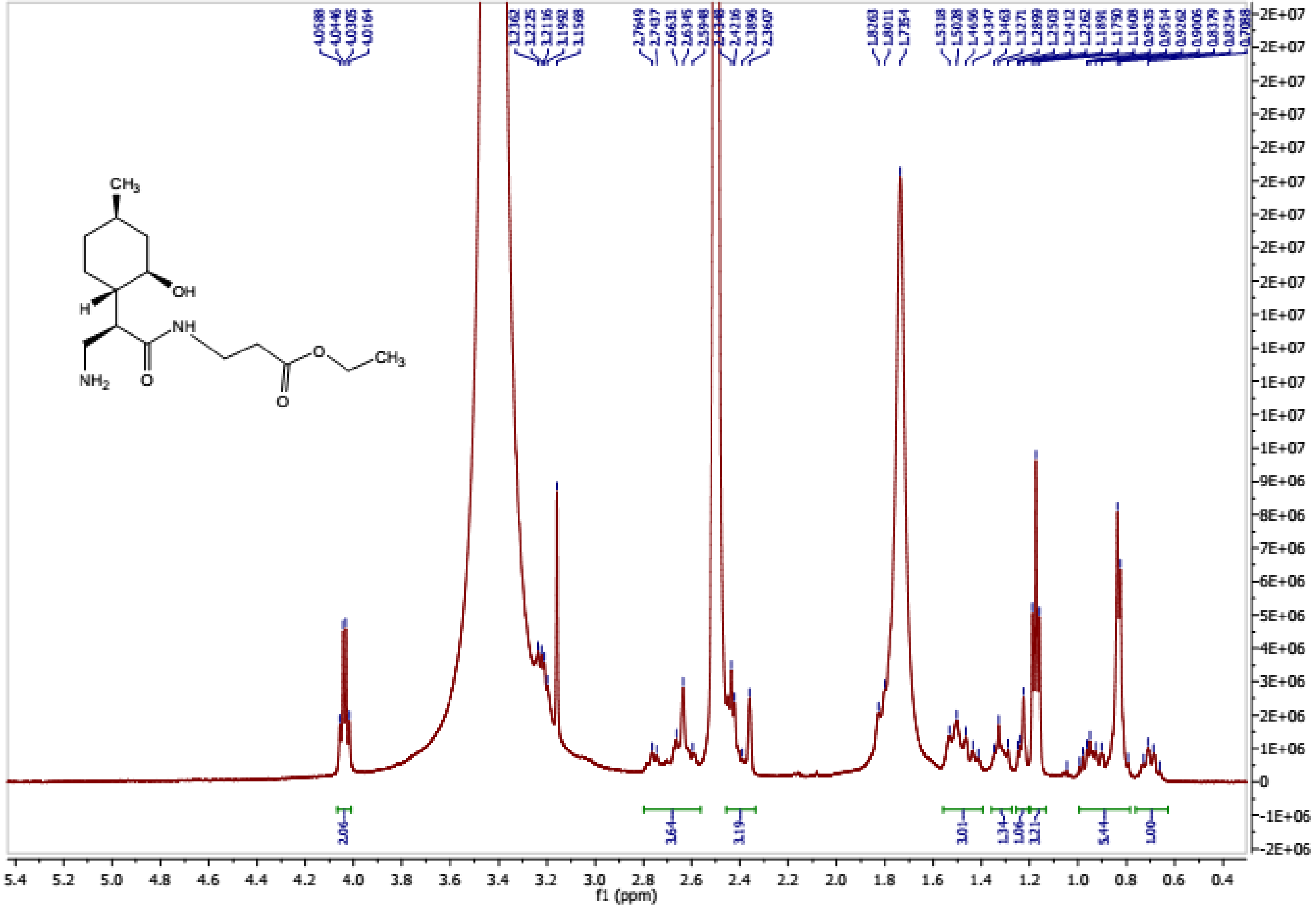

^{13}C-NMR of compound **30**

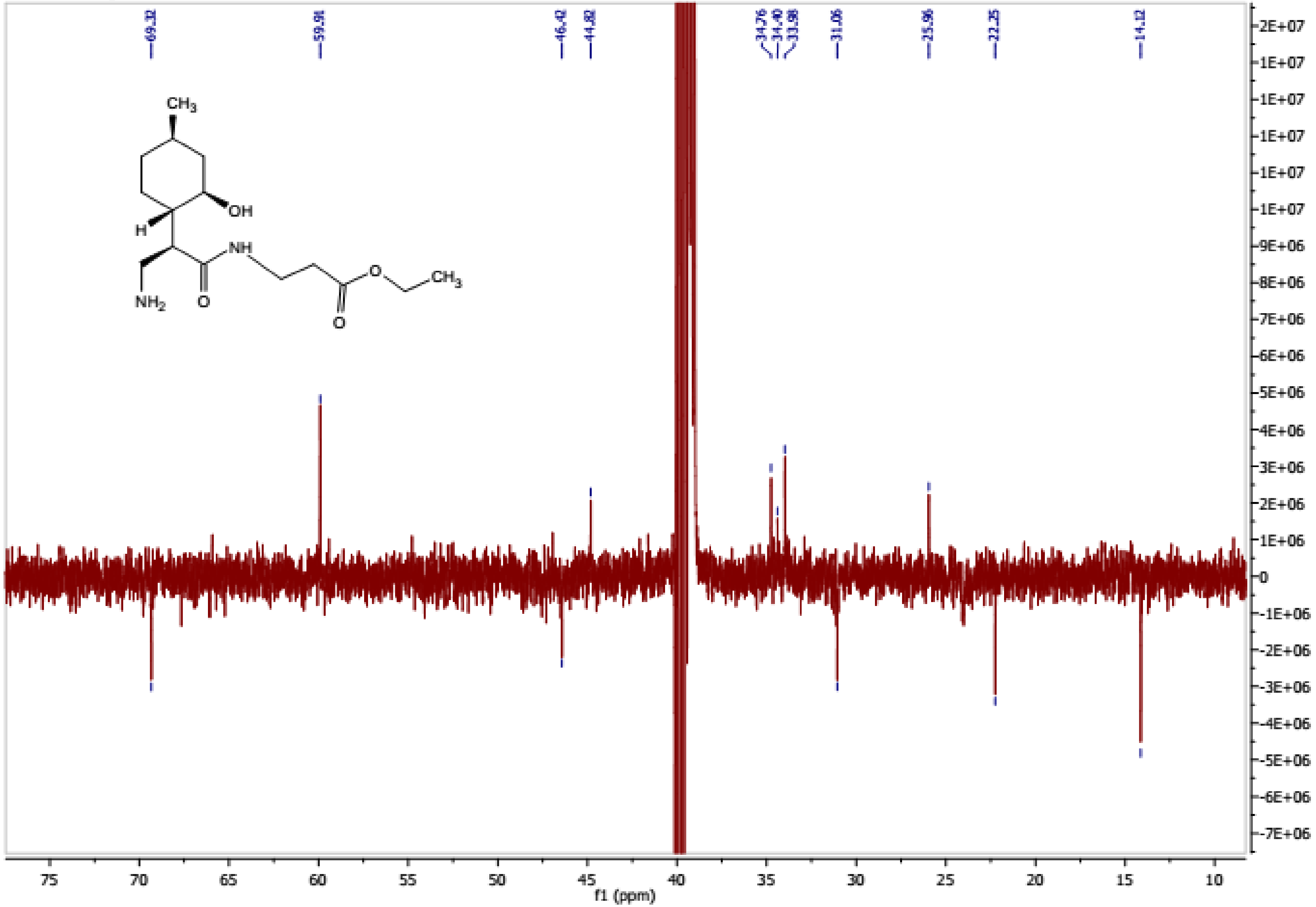

^{1}H-NMR of compound **31**

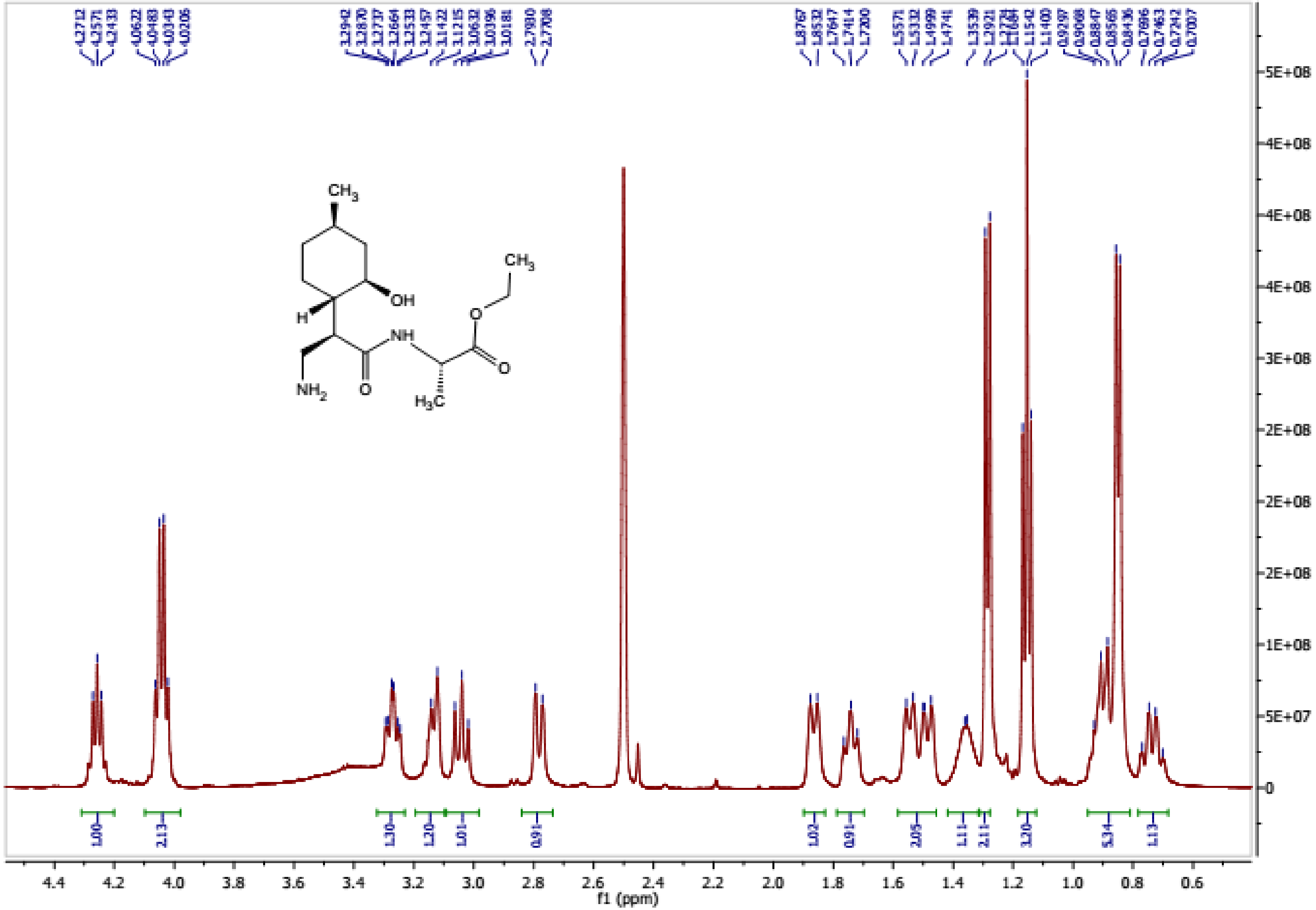

^{13}C-NMR of compound **31**

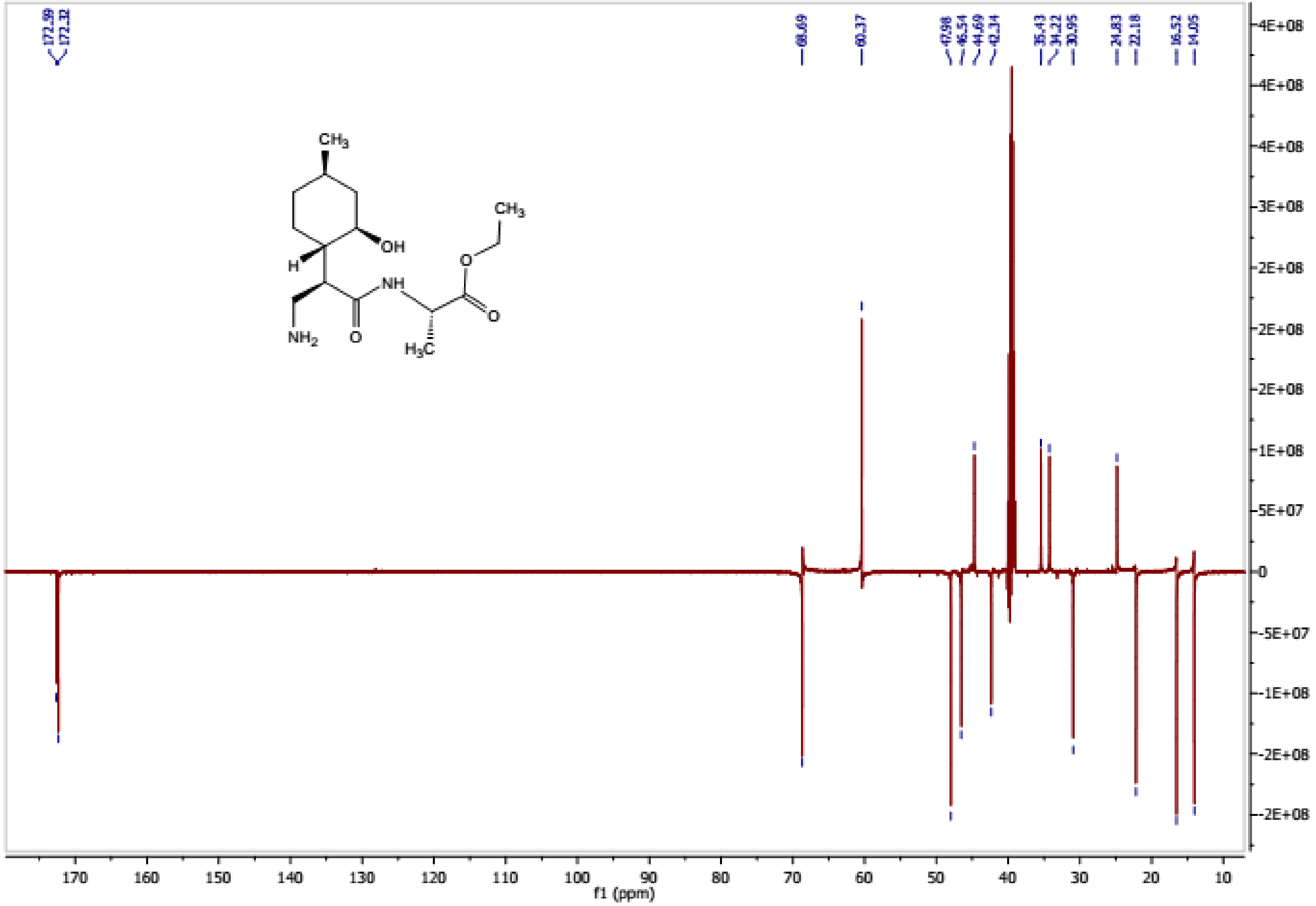

^{1}H-NMR of compound **32**

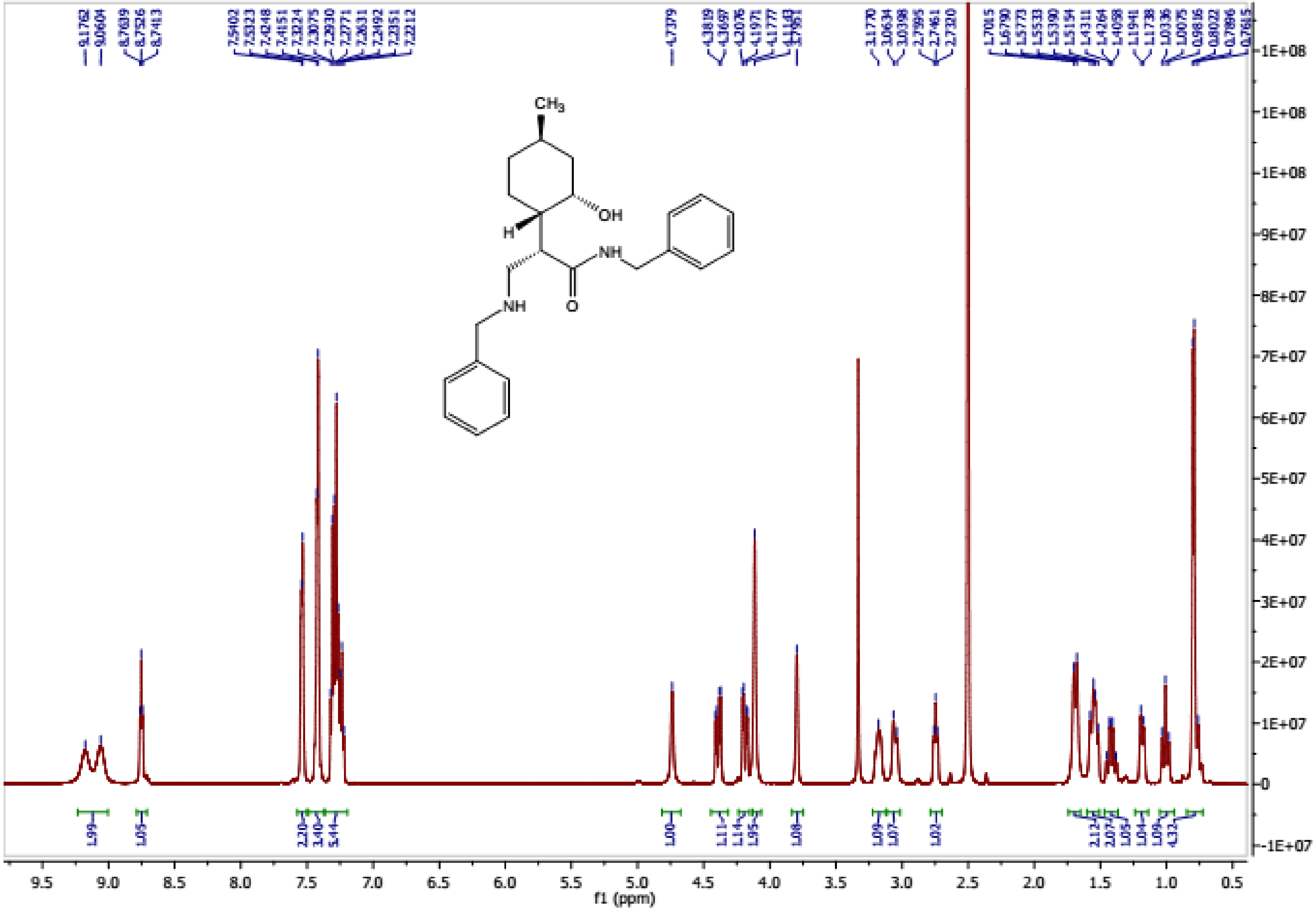

^{13}C-NMR of compound **32**

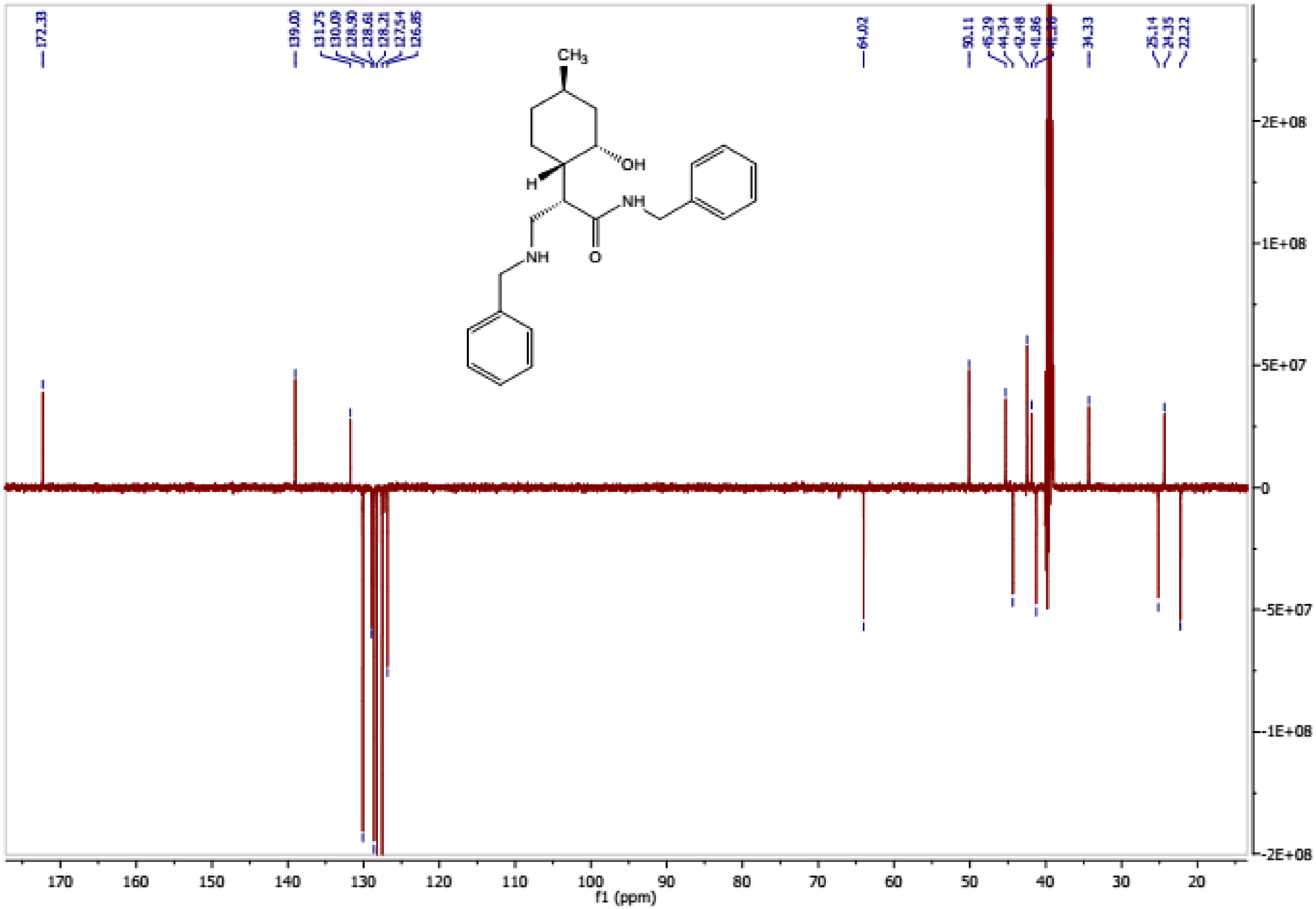

^{1}H-NMR of compound **33**

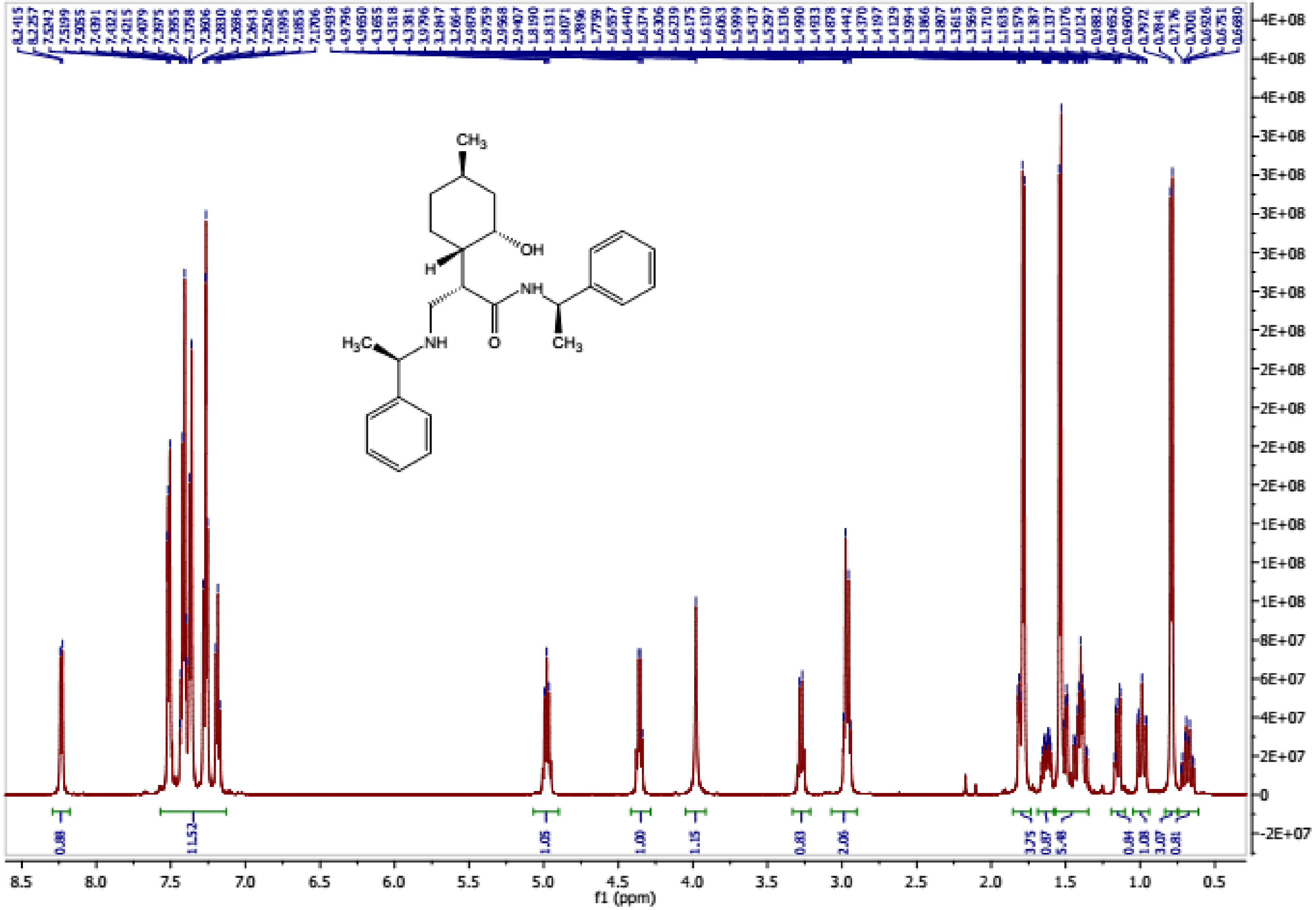

^{13}C-NMR of compound **33**

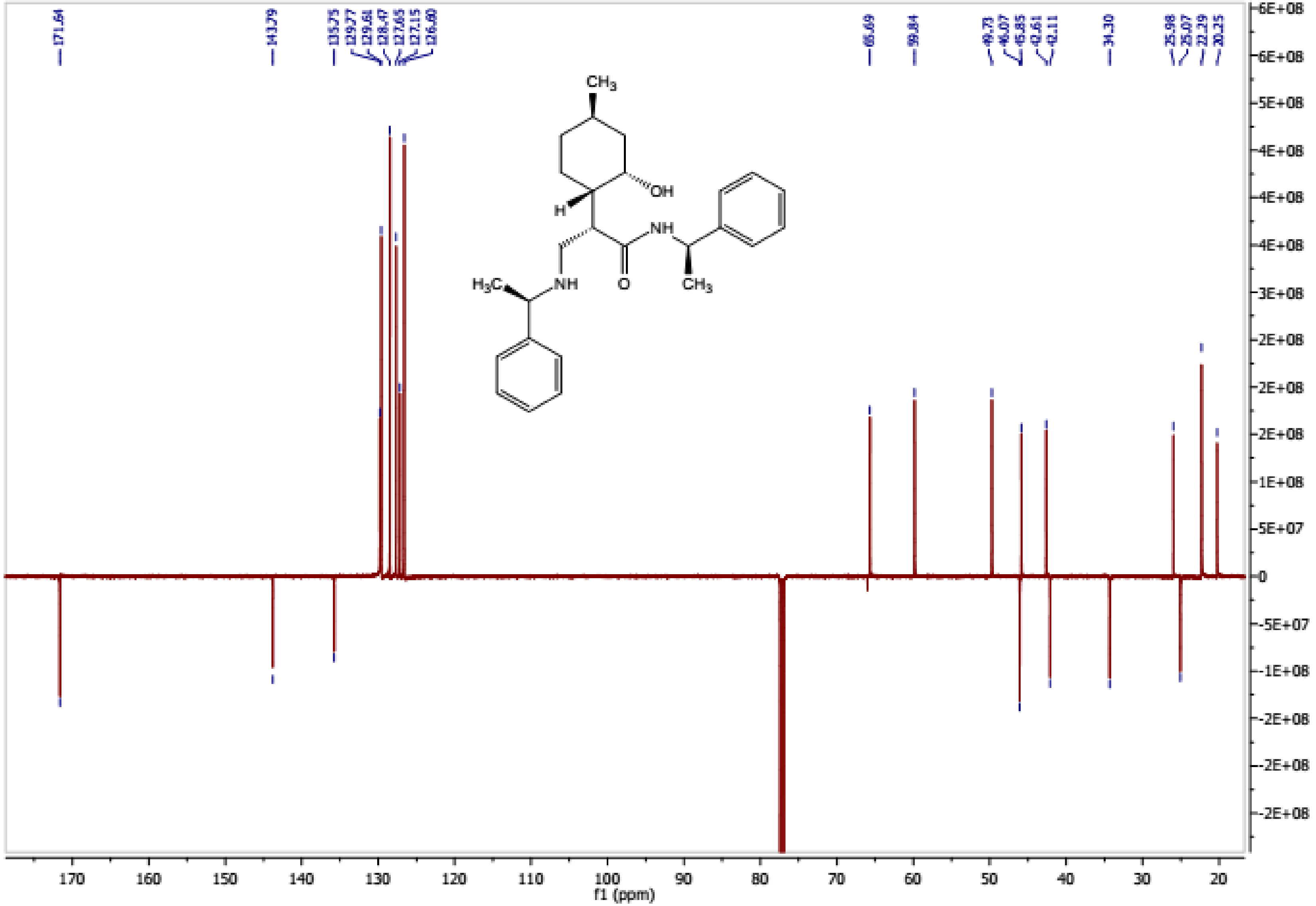

^{1}H-NMR of compound **34**

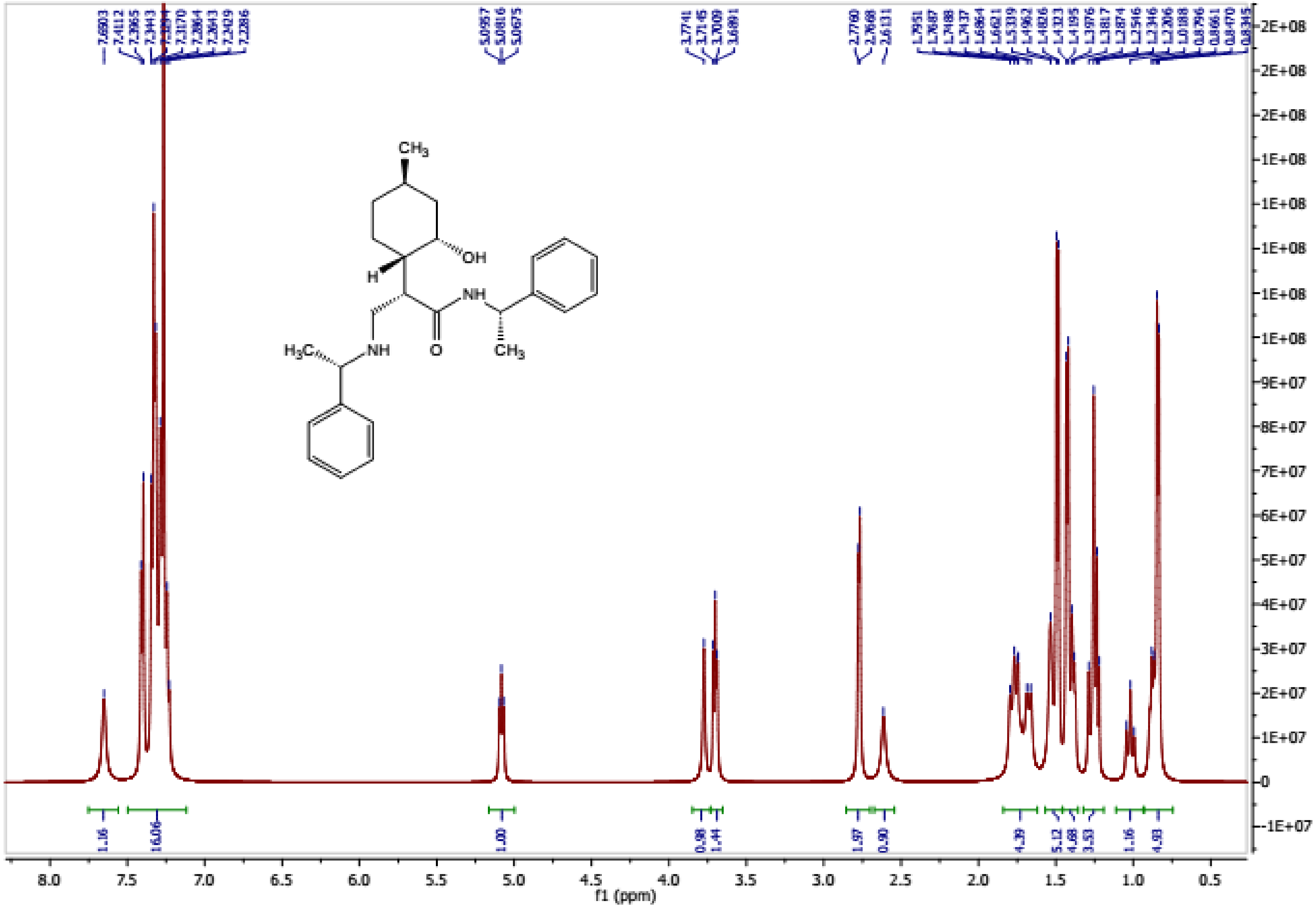

^{13}C-NMR of compound **34**

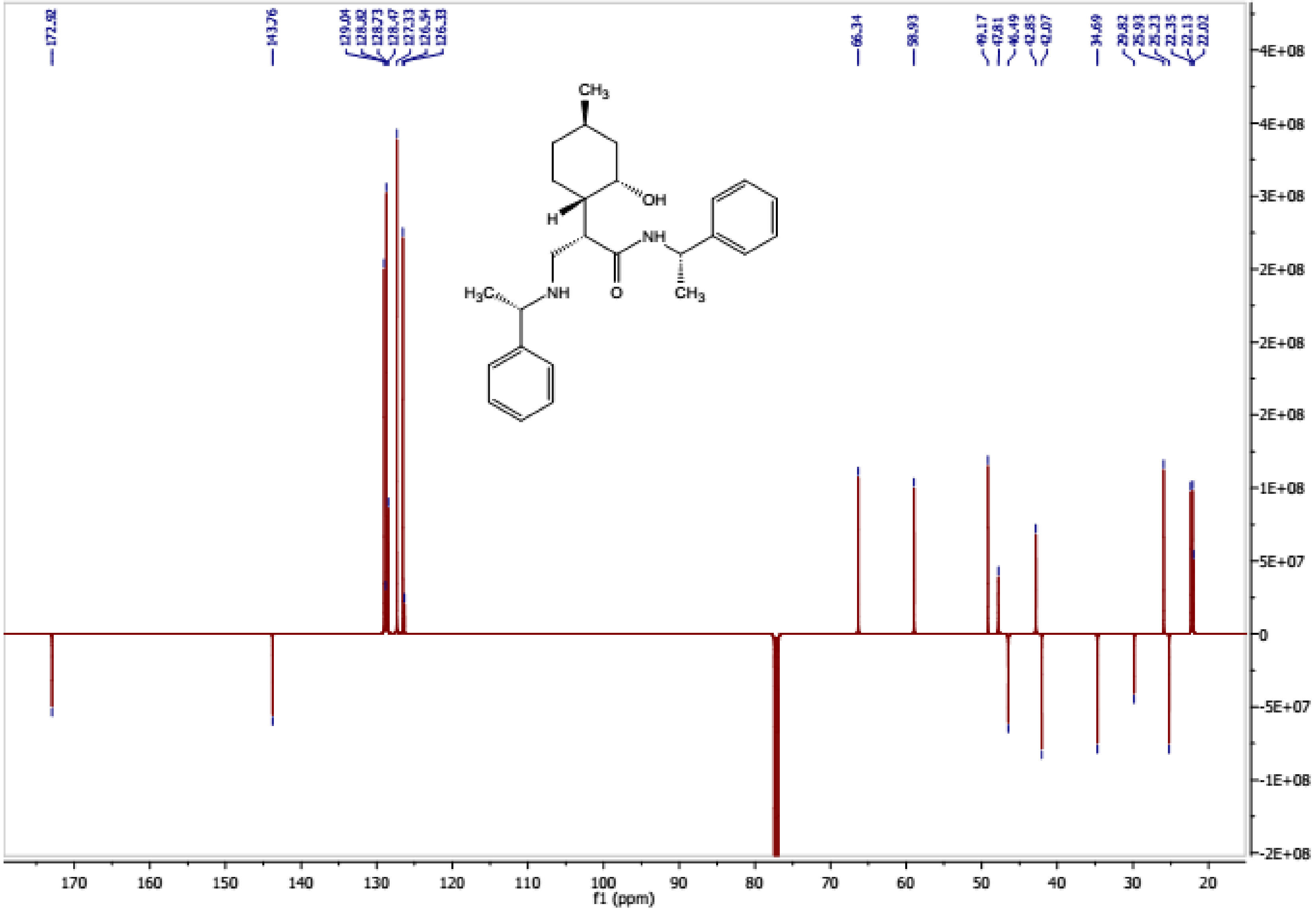

^{1}H-NMR of compound **35**

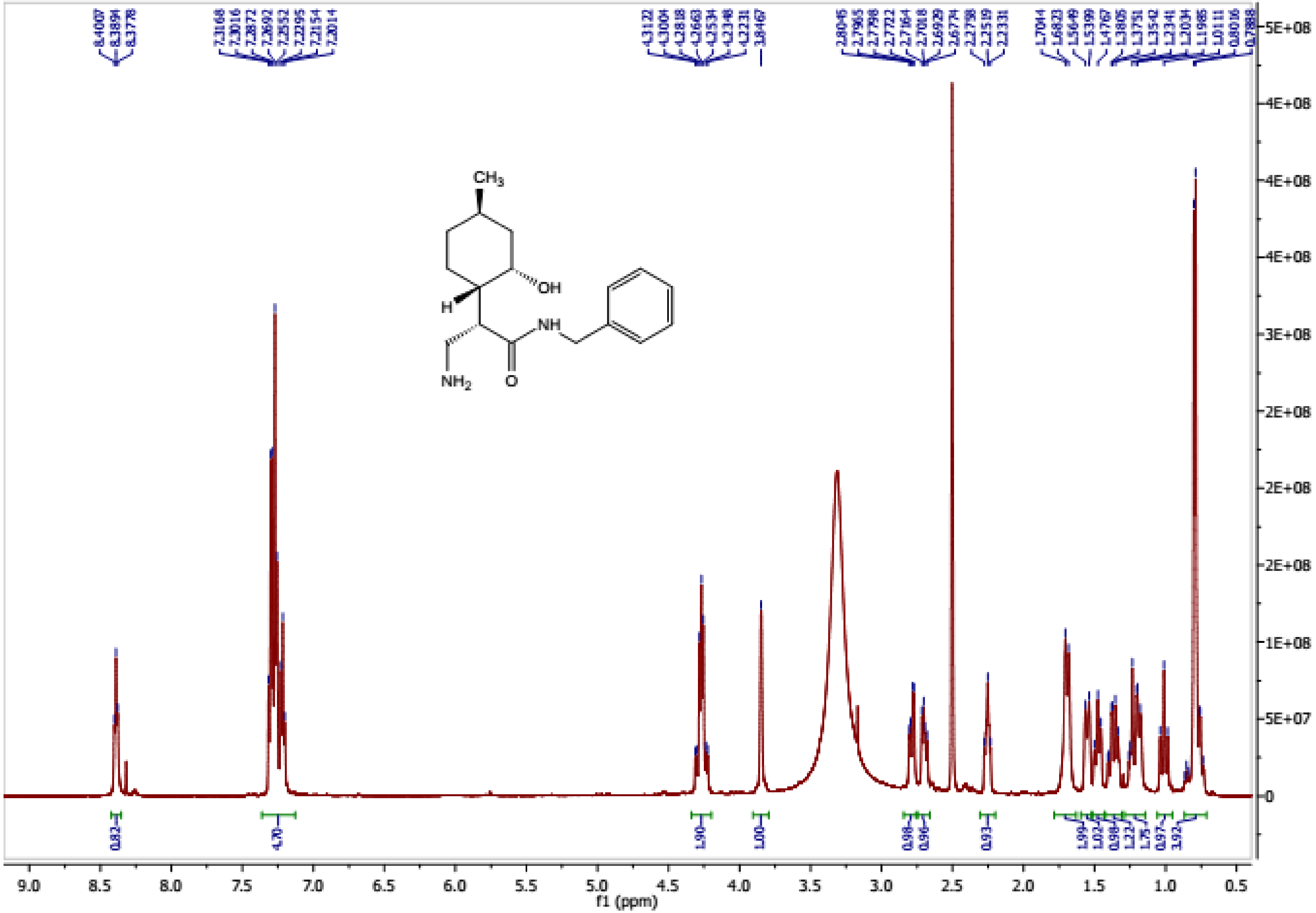

^{13}C-NMR of compound **35**

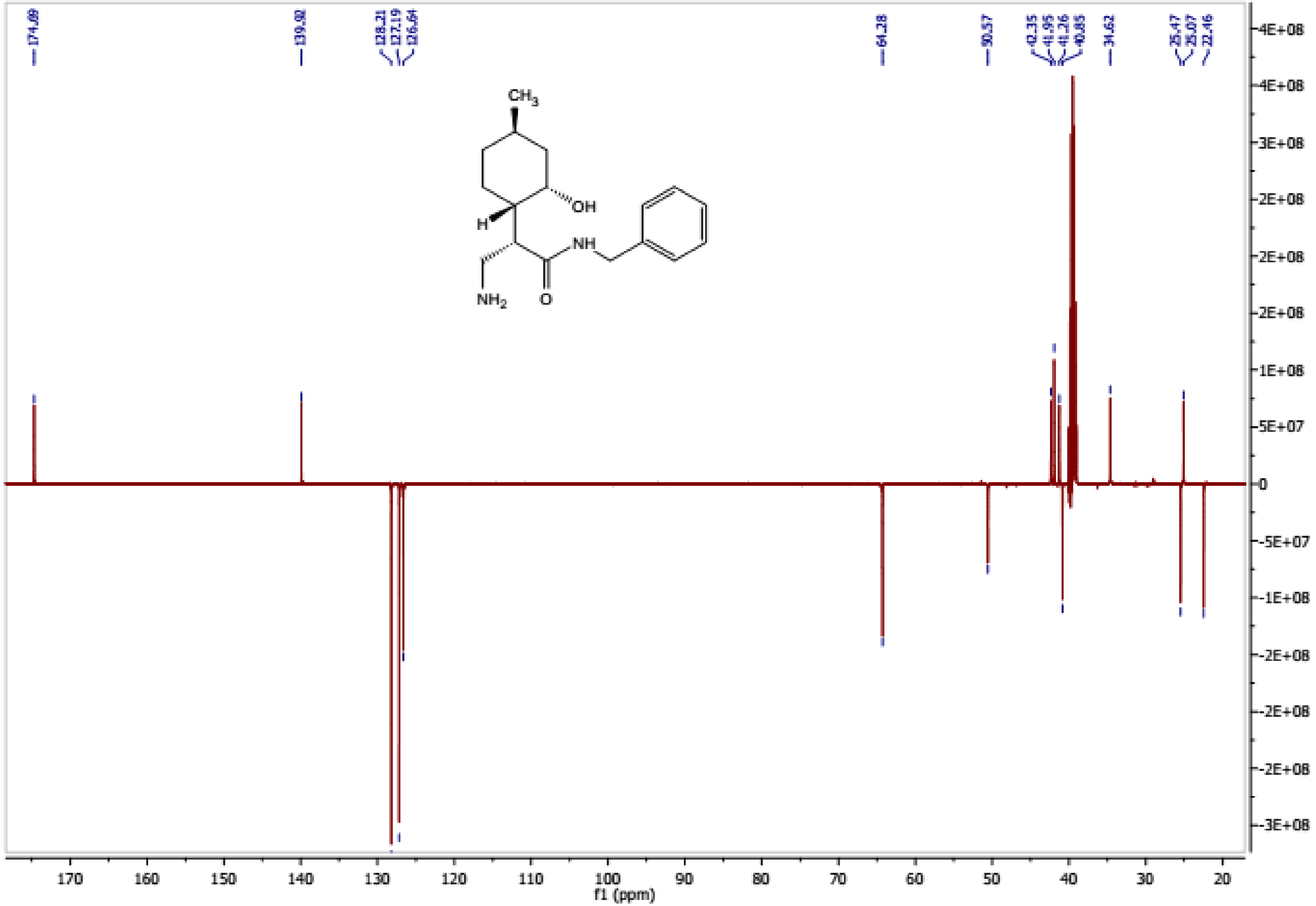

^{1}H-NMR of compound **36**

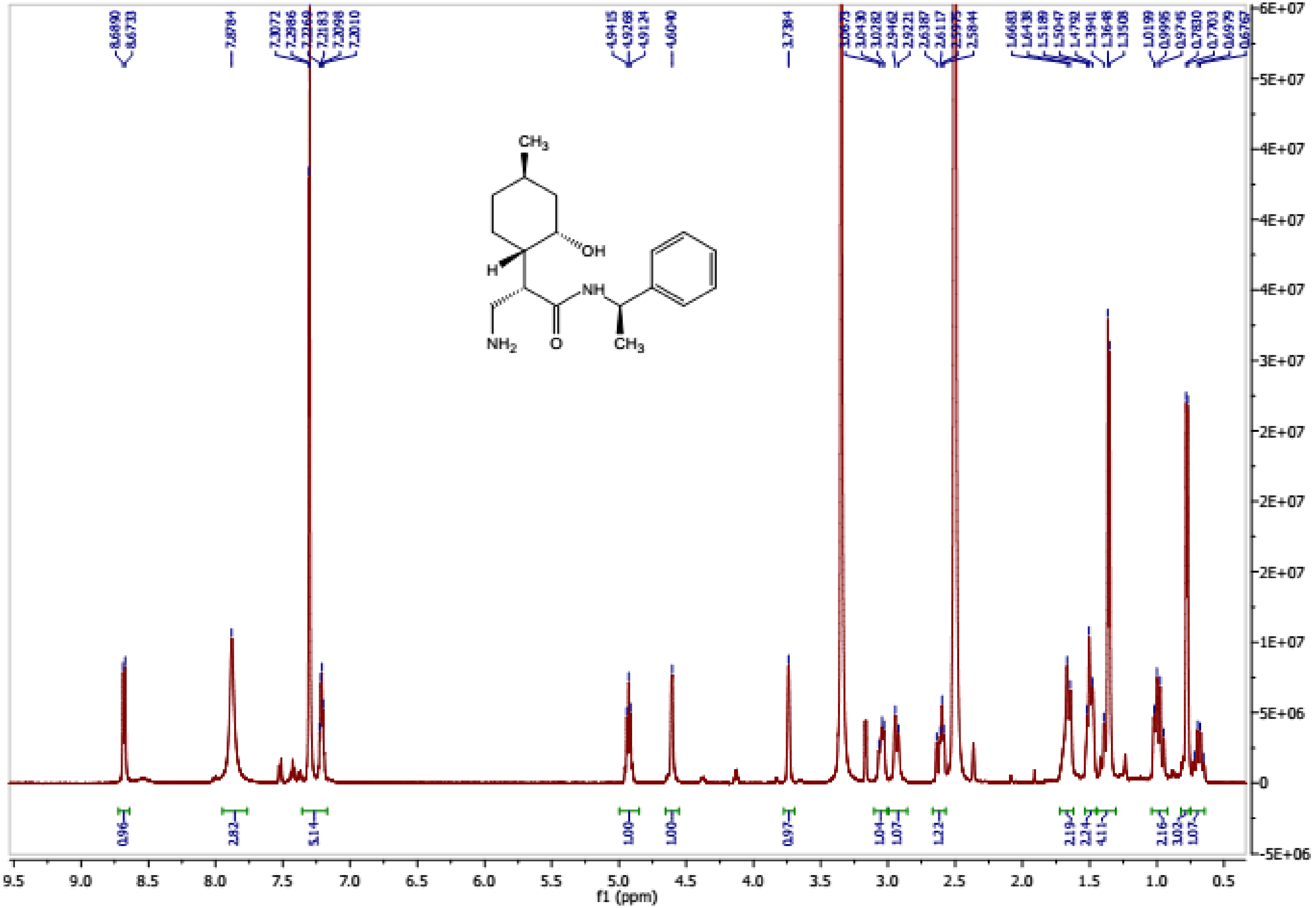

^{13}C-NMR of compound **36**

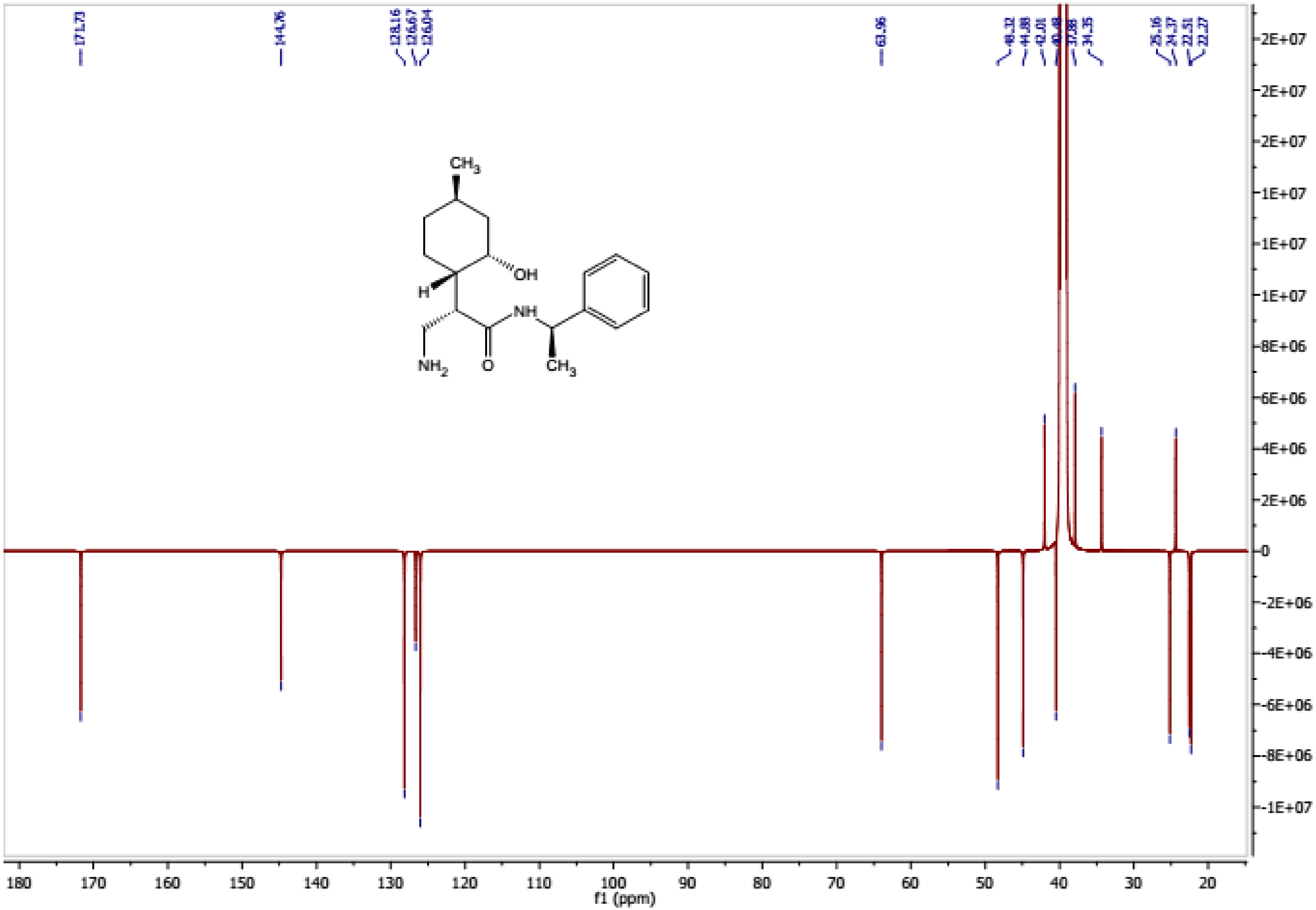

^{1}H-NMR of compound **37**

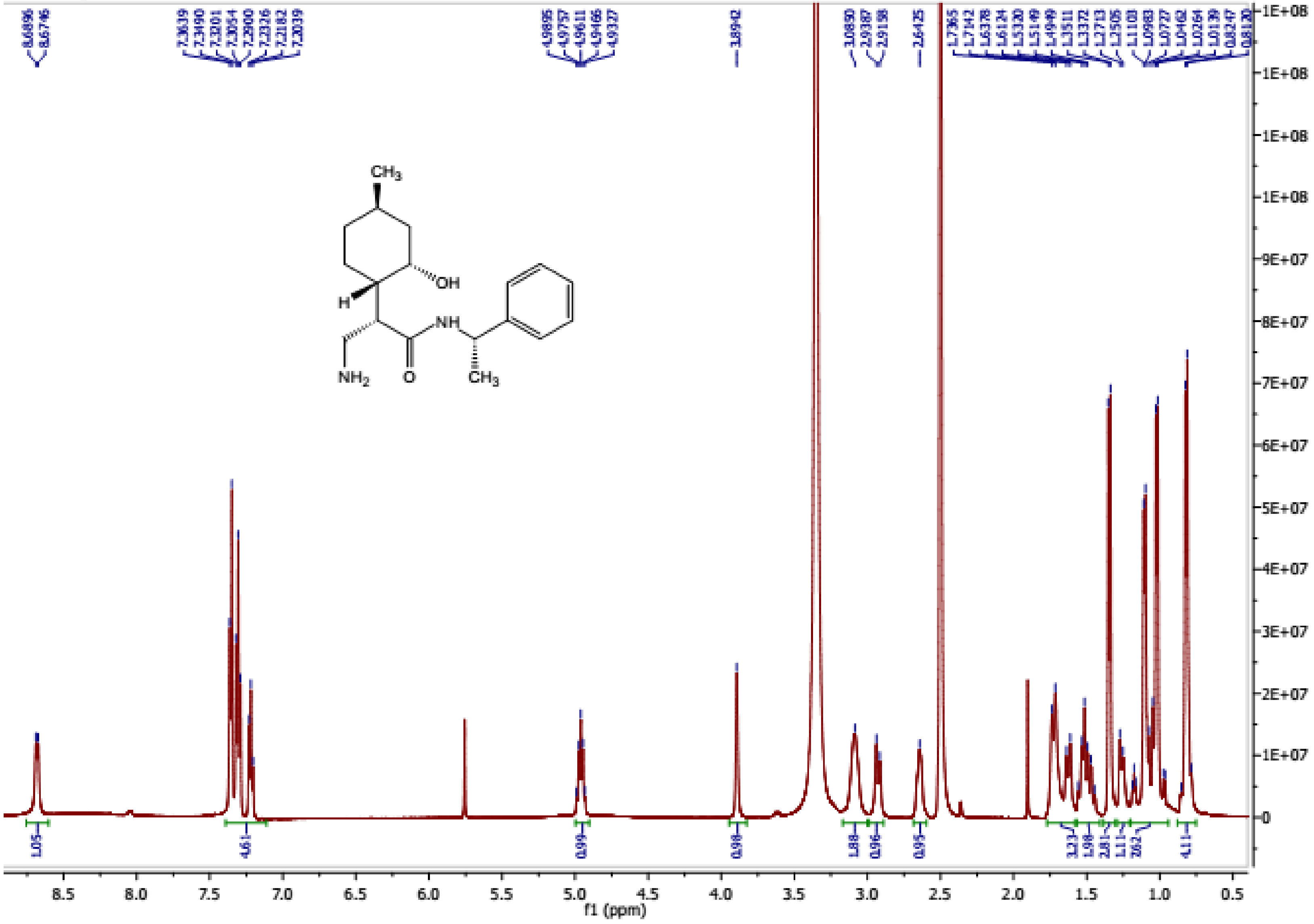

^{13}C-NMR of compound **37**

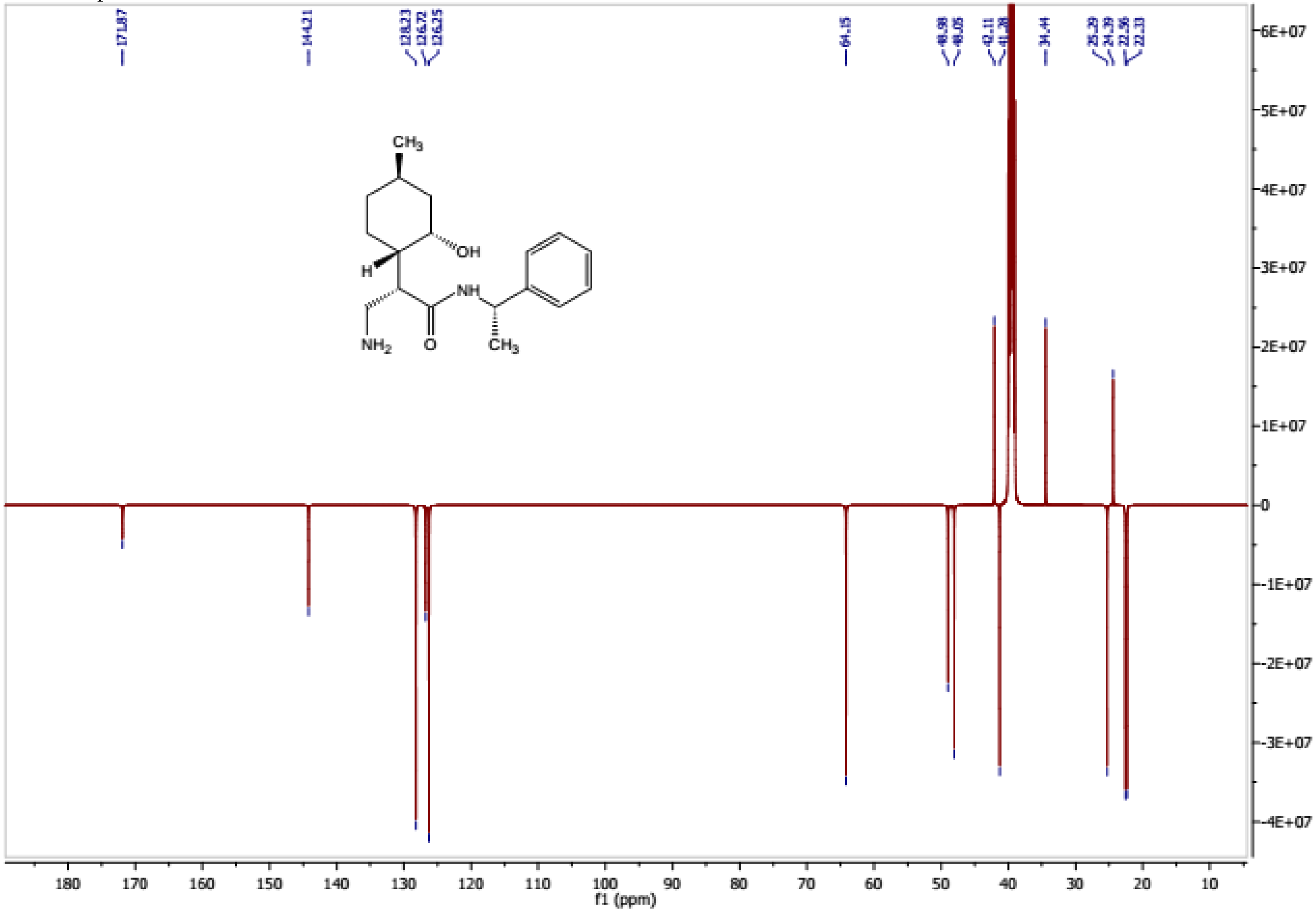

Supplement: Supplementary file 1 [file ijms-19-03522-s001.pdf]
